# Activation of HIF-1α C-terminal transactivation domain protects against hypoxia-induced kidney injury through hexokinase 2-mediated mitophagy

**DOI:** 10.1038/s41419-023-05854-5

**Published:** 2023-05-24

**Authors:** Zuo-Lin Li, Lin Ding, Rui-Xia Ma, Yue Zhang, Yi-Lin Zhang, Wei-Jie Ni, Tao-Tao Tang, Gui-Hua Wang, Bin Wang, Lin-Li Lv, Qiu-Li Wu, Yi Wen, Bi-Cheng Liu

**Affiliations:** 1 Institute of Nephrology, Zhong Da Hospital, Southeast University School of Medicine, Nanjing, Jiangsu China; 2 Department of Nephrology, The Affiliated Hospital of Qingdao University, Qingdao, Shandong China

**Keywords:** Acute kidney injury, Preclinical research

## Abstract

The transcription factor hypoxia-inducible factor-1α (HIF-1α), as a master regulator of adaptive responses to hypoxia, possesses two transcriptional activation domains [TAD, N-terminal (NTAD), and C-terminal (CTAD)]. Although the roles of HIF-1α NTAD in kidney diseases have been recognized, the exact effects of HIF-1α CTAD in kidney diseases are poorly understood. Here, two independent mouse models of hypoxia-induced kidney injury were established using HIF-1α CTAD knockout (HIF-1α CTAD^−/−^) mice. Furthermore, hexokinase 2 (HK2) and mitophagy pathway are modulated using genetic and pharmacological methods, respectively. We demonstrated that HIF-1α CTAD^−/−^ aggravated kidney injury in two independent mouse models of hypoxia-induced kidney injury, including ischemia/reperfusion-induced kidney injury and unilateral ureteral obstruction-induced nephropathy. Mechanistically, we found that HIF-1α CTAD could transcriptionally regulate HK2 and subsequently ameliorate hypoxia-induced tubule injury. Furthermore, it was found that HK2 deficiency contributed to severe renal injury through mitophagy inhibition, while mitophagy activation using urolithin A could significantly protect against hypoxia-induced kidney injury in HIF-1α C-TAD^−/−^ mice. Our findings suggested that the HIF-1α CTAD-HK2 pathway represents a novel mechanism of kidney response to hypoxia, which provides a promising therapeutic strategy for hypoxia-induced kidney injury.

## Introduction

Hypoxia, one of the most prevalent pathophysiological conditions, is a crucial instigator of a variety of kidney diseases [1, 2]. Although convincing evidence has indicated that the severity and duration of hypoxia determine the renal prognosis [2, 3], the exact molecular responses of the kidney to hypoxia remain largely enigmatic.

It is well known that the transcription factor hypoxia-inducible factor-1 (HIF-1) is essential for cellular adaptations to hypoxia [4], which is tightly regulated by post-translational modifications by at least two separate oxygen-dependent mechanisms: hydroxylation of proline residues by prolyl hydroxylase domain (PHD) and asparagine hydroxylation by factor-inhibiting HIF-1 (FIH-1) [5]. Structural biology studies revealed that HIF-1, as a master transcription factor, possesses two transcriptional activation domains [TAD; N-terminal (NTAD) and C-terminal (CTAD)], which are regulated by PHD and FIH-1, respectively [6]. Furthermore, convincing studies have demonstrated that NTAD and CTAD could transcribe distinct genes, performing their own function [6, 7]. Although the roles of HIF-1 have been studied in a number of kidney diseases, as evidenced by genetic and pharmacological modulation, separate functions of CTAD and NTAD in hypoxic kidney diseases remain obscure.

Currently, individuals with renal anemia have been treated with a specific PHD inhibitor, which stimulates the function of HIF-1α NTAD [8]. Interestingly, pre-clinical studies have demonstrated that activation of HIF-1α NTAD induced by the PHD inhibitor protects against acute kidney injury (AKI) and chronic kidney disease. For instance, Wu et al. [9] demonstrated that activating HIF-1α NTAD protects the kidneys from ischemia/hypoxia. Meanwhile, it was confirmed that activating HIF-1 NTAD has renoprotective effects on diabetic nephropathy [10]. These data strongly suggested that HIF-1α NTAD might provide a protective effect against kidney diseases. However, the precise role of HIF-1α CTAD in hypoxic kidney diseases is not well understood.

Here, we established the HIF-1α CTAD knockout (HIF-1α CTAD^−/−^) mice to assess the potential pathophysiological function of HIF-1α CTAD in hypoxic kidney disease. Interestingly, we demonstrated that HIF-1α CTAD protects against hypoxia-induced kidney injury through hexokinase 2 (HK2)-mediated mitophagy. Our findings represent a novel perspective on the hypoxia response signaling in the kidney, which provides a promising therapeutic strategy for hypoxic kidney injury.

## Methods and materials

### Animals

All animal experiments were performed in accordance with the standard guidelines for the care and use of laboratory animals and were approved by the Ethics Committee of Southeast University. HIF-1α CTAD^−/−^ mice were generated using a CRISPR/Cas9-based approach. The *HIF-1α* gene was edited by homologous recombination. Briefly, Cas9 mRNA and gRNA are obtained by in vitro transcription. The homologous recombination vector (donor vector) was constructed using In-Fusion cloning, which contains a 3.0 kb 5′ homologous arm and a 3.0 kb 3′ homologous arm. Then, the Cas9 mRNA, gRNA, and donor vector were microinjected into the fertilized eggs of C57BL/6J mice to obtain F0 generation mice. Finally, the F0 generation mice, which were identified by polymerase chain reaction (PCR) amplification and sequencing, were mated with normal C57BL/6J mice to obtain the F1 generation mice. By crossing heterozygous parental lines, homozygous and wild-type (WT) littermates were obtained. Male mice (6–8 weeks old, weighing 20–22 g) were utilized for experiments, and the mouse models of renal ischemia/reperfusion (I/R) injury and unilateral ureteral obstruction (UUO) nephropathy were established as previously reported [11, 12]. For the bilateral I/R injury, both renal pedicles were clamped using microaneurysm clamps for 35 min. Sham operations underwent the same surgical procedures without clamping the renal pedicles. The body temperature was controlled between 36.5 °C and 37.0 °C using a sensitive rectal probe throughout the procedure. For UUO, the left ureter of the mouse was ligated just below the renal pelvis. Sham mice underwent identical surgical procedures, except that the ureter was not ligated. Mice were euthanized on day 1 following I/R injury or day 3 following UUO under general anesthesia, and their kidneys were harvested.

### Treatment with urolithin A and in vivo lentiviral transduction

Before establishing the I/R injury model, the mice were subjected to daily oral administration of urolithin A (MedChemExpress, Monmouth Junction, NJ, USA) at a dose of 50 mg/kg body weight for two consecutive days to induce mitophagy. For lentiviral gene transfer, lentiviruses expressing HK2 (Lv-HK2) or negative control (Lv-NC) were obtained from GenePharma (Shanghai, China). On day 3, prior to I/R or UUO surgery, Lv-mediated gene transfer in the kidneys of I/RI mice was performed by tail vein injection (5 × 10^8^ TU/mouse). The mice were euthanized on day 1 following I/RI or on day 3 following the clamping of renal pedicles. Subsequently, serum and kidney tissues were harvested.

### The RNA-sequencing and bioinformatics analysis

We characterized the expression patterns of mRNA genes in the kidney cortex following I/R injury using RNA sequencing. The pathway analyses based on Gene Ontology (GO) and Kyoto Encyclopedia of Genes and Genomes (KEGG; https://www.genome.jp/kegg/kegg1.html) databases were employed to predict the function of differentially expressed genes.

### Culture and treatment of primary tubular epithelial cells

Kidneys were harvested from 6 weeks old mice, and their tubular epithelial cells (TECs) were isolated for primary culture using established methods [13]. The TECs were cultured in a humidified atmosphere of 5% CO_2_ and 95% O_2_ at 37 °C. In order to induce hypoxic injury, the cells were cultured for 12 h under hypoxic conditions (1% O_2_, 94% N_2_, and 5% CO_2_) in the glucose-free medium. Following hypoxic treatment, the cells were returned to a regular culture medium with oxygen for 2 h to reoxygenate (hypoxia/reoxygenation, H/R). The control cells were incubated with a regular medium in a regular incubator (5% CO_2_ and 21% O_2_). For adenoviral (Ad) transfection, cells grown to 70% confluency were transduced with Ad-NC or Ad-CTAD containing the bHLH-PAS domain for 24 h according to the manufacturer’s instructions and thereafter exposed to H/R conditions for 24 h. Ad-NC was used as a negative control.

### Chromatin immunoprecipitation assay

Chromatin immunoprecipitation (ChIP) assay was performed using the Simple ChIP Plus Enzymatic Chromatin IP Kit (Magnetic Beads, #9003, Cell Signaling Technology) according to previous descriptions [12]. IP was performed using an antibody against HIF-1α (ab2185, Abcam) or IgG as the negative control. PCR with specific primers of the promoter region of HK2 was utilized to detect the precipitated DNA fragments.

### Luciferase reporter assay

The plasmids, such as pGL3-HRE-HK2-Luc luciferase reporter plasmid, HIF-1α (HIF-1α TAD, containing NTAD and CTAD), HIF-1α NTAD, and HIF-1α CTAD in the pSD11 vector were constructed by GenePharma (Shanghai, China). HIF-1α NTAD-Gal4 and HIF-1α CTAD-Gal4 were constructed by inserting the sequences of Addgene plasmid #18955 into the backbone of Addgene plasmid #24887 as described previously [14]. The corresponding plasmids were transfected into target cells using Lipofectamine 3000 in accordance with the manufacturer’s instructions (Invitrogen Corp., Carlsbad, CA, USA). Detailed methods for the luciferase reporter assay have been established, as previously reported [12].

### Transmission electron microscopy (TEM)

The renal cortex was immersed in a fixative that contained 2.5% glutaraldehyde and 4% paraformaldehyde. Sample handling and detection were conducted by the electron microscopic core lab at Southeast University (H-7650, Hitachi, Japan). The mitophagy in TECs was quantified.

### Mitochondria isolation

Mitochondria were isolated using the mitochondrial isolation kit (BioVision, Inc. CA, USA) according to the manufacturer’s protocol. In brief, after washing with ice-cold PBS, cells were centrifuged at 600*g* for 5 min and resuspended in 1× Cytosol Extraction Buffer. Then, homogenization was centrifuged at 1200*g* for 10 min at 4 °C to remove nuclei and intact cells. The collected supernatant was centrifuged at 10,000 g for 30 min at 4 °C. Finally, the pellets were resuspended in 1× Cytosol Extraction Buffer and centrifuged at 10,000*g* for 30 min at 4 °C to obtain mitochondria.

### Extraction of RNA for quantitative real-time PCR

The total RNA was extracted using TRIzol (Takara, Japan), following which cDNA was synthesized using a PrimeScript RT reagent kit (Takara) in accordance with the manufacturer’s instructions. Using an ABI PRISM 7300 real-time PCR System (Applied Biosystems, USA), real-time PCR (RT-PCR) was performed. The relative mRNA expression was normalized to β-actin. All the primers for RT-PCR are listed in Supplementary Table 1.

### Immunohistochemistry staining

For immunohistochemistry staining, after antigen retrieval using the antigen retrieval buffer EDTA at pH 9.0 and heating for 15 min at 100 °C, the formalin-fixed and paraffin-embedded tissue sections of kidney were incubated with primary antibodies against kidney injury molecular-1 (KIM-1, MA5–28211, Invitrogen), F4/80 (ab6640, Abcam, Cambridge, MA), HK2 (ab209847, Abcam), PTEN-induced putative kinase 1 (PINK1, ab23707, Abcam), Bcl-2 19-kDa interacting protein 3 (BNIP3, ab109362, Abcam), or cytochrome C oxidase subunit I (ab14705, Abcam), and thereafter analyzed using a streptavidin peroxidase detection system (Maixin Biotech, Fuzhou, China) in accordance with the manufacturer’s protocol. Diaminobenzidine (Maixin) was used as horseradish peroxidase (HRP)-specific substrate.

### Western blotting

For immunoblot analysis, the renal cortex, primary TECs, or mitochondria were prepared as previously described [11]. Anti-HIF-1α (ab1, Abcam), anti-HIF-1α-C-terminal (ab228649, Abcam), anti-LC3 (ab192890, Abcam), anti-p62 (ab109012, Abcam), anti-HK2 (ab209847, Abcam), PINK1 (ab23707, Abcam), BNIP3 (ab109362, Abcam), anti-COX IV (ab16056, Abcam), and anti-β-actin (AY0573, Abways, China) were employed as the primary antibodies. Horseradish peroxidase-conjugated anti-rabbit immunoglobulin G (IgG) or anti-mouse IgG (Abcam) antibodies were employed as secondary antibodies. An ECL Plus reagent was used to detect the signals (Thermo Fisher Scientific). The intensity values expressed as the relative expression of protein were normalized to β-actin or COX IV.

### Statistical analyses

Data are presented as mean ± SEM. The data were analyzed using a Student’s *t*-test or Mann–Whitney *U* test by SPSS version 20.0 (IBM Corp., Armonk, NY, USA). A two-sided *p*-value of <0.05 was considered significant.

## Results

### HIF-1α CTAD^−/−^ aggravates renal injury in I/R-induced kidney injury

**Fig. 1:**
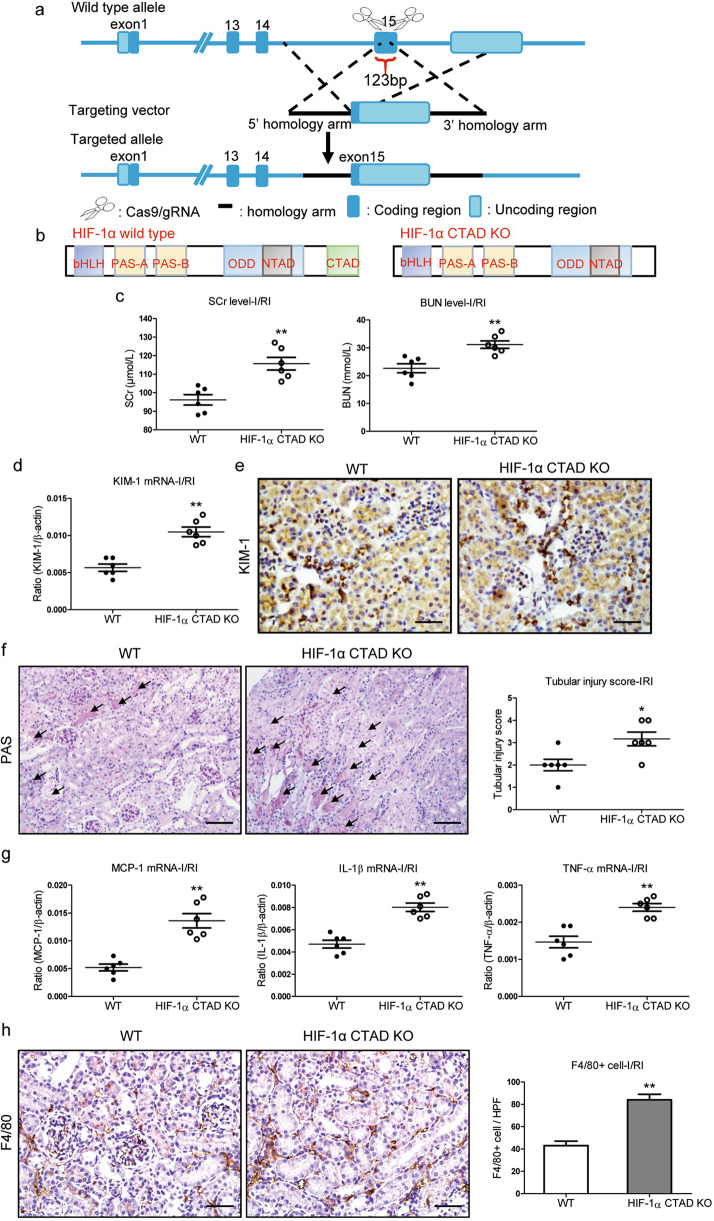
HIF-1α CTAD KO results in severe renal injury in I/R-induced AKI. **a** Construction strategy for HIF-1α CTAD^−/−^ mouse. **b** Schematic representation of the proposed HIF-1α CTAD KO. **c** SCr and BUN levels on day 1 following 35 min of ischemia/reperfusion-induced renal injury (I/RI) (*n* = 6). **d** Quantitative real-time polymerase chain reaction (qRT-PCR) analysis of KIM-1 expression. The relative levels were normalized to β-actin (*n* = 6). **e** Representative images of KIM-1 staining (*n* = 6). Scale bars, 50 µm. **f** Representative histological changes (periodic acid-Schiff [PAS] staining) on day 1 following 35 min of I/RI (left, *n* = 6). Scale bar, 100 µm. The quantification of tubular injury based on PAS staining (right, *n* = 6). **g** mRNA expression of inflammatory factors (monocyte chemoattractant protein-1 [MCP-1], tumor necrosis factor-α [TNF-α], and interleukin-1β [IL-1β]) in the I/R injured kidney assessed by qRT-PCR. The relative levels were normalized to β-actin (*n* = 6). h. Representative F4/80 stained kidney sections from I/R injured mice (left). Scale bars, 50 µm. The semiquantitative population of F4/80+ macrophages infiltrating the I/R injured kidney (right). Data are shown as mean ± SEM of 6 mice. **p-value < 0.01 versus the WT mice (*t-*test).

HIF-1α C-TAD^−/−^ mice were generated to explore the pathophysiological function of HIF-1α CTAD (Fig. 1a), and a schematic illustration of HIF-1α CTAD knockout is provided in Fig. 1b. Moreover, a specific anti-HIF-1α CTAD antibody was utilized to corroborate the results of HIF-1α CTAD knockout (Supplementary Fig. S1). We noticed that the levels of serum creatinine (SCr) and blood urea nitrogen (BUN) were significantly increased in HIF-1α CTAD^−/−^ mice (Fig. 1c). In kidney of HIF-1α CTAD^−/−^ mice, the expression of KIM-1, a marker of tubules injury, was also increased (Fig. 1d, e). Meanwhile, as illustrated in Fig. 1f, bilateral renal injury in the WT group resulted in an increase of necrosis and detachment of tubules, accumulation of cellular debris, and formation of casts; whereas these injury parameters were significantly aggravated in HIF-1α CTAD^−/−^ group. In addition, the mRNA expressions for tumor necrosis factor-α (TNF-α), interleukin-1β (IL-1β), and monocyte chemoattractant protein-1 (MCP-1) were upregulated in those mice (Fig. 1g). Concomitantly, more macrophage infiltration was observed in injured kidneys of HIF-1α CTAD^−/−^ mice (Fig. 1h). These data demonstrated that HIF-1α CTAD knockout aggravates renal injury in ischemic AKI.

### HIF-1α CTAD^−/−^ enhances renal injury in UUO mice

**Fig. 2:**
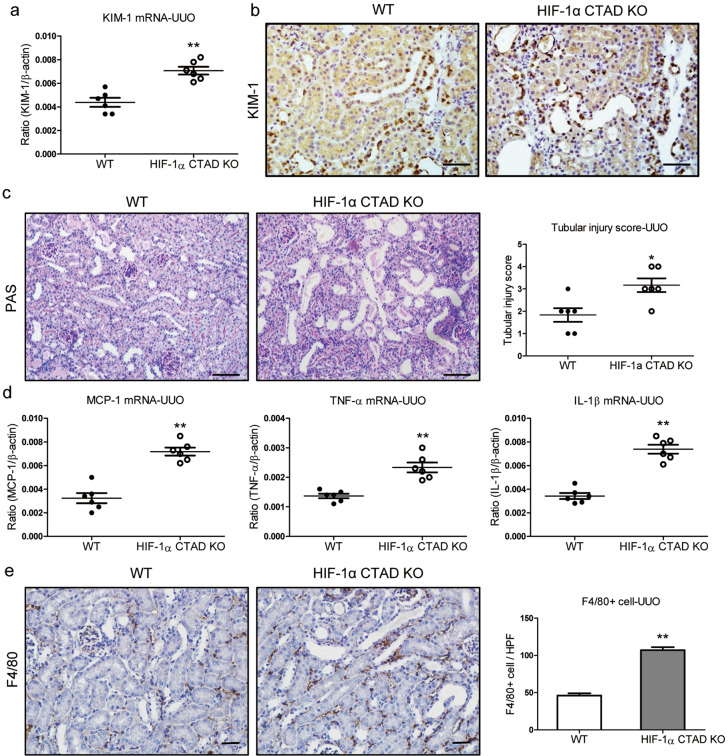
Severe renal injury in HIF-1α CTAD^−/−^ mice with UUO. **a** qRT-PCR analysis of KIM-1 expression. The relative levels were normalized to β-actin (*n* = 6). **b** Representative images of KIM-1 expression in kidney tissues from UUO mice were assessed by immunohistochemistry (*n* = 6). Scale bars, 50 µm. **c** Histological changes (PAS staining) on day 3 after UUO (left, *n* = 6). Scale bar, 100 µm. The quantification of tubular injury based on PAS staining (right, *n* = 6). **d** mRNA expression of inflammatory factors (MCP-1, TNF-α, and IL-1β) in the UUO kidney assessed by qRT-PCR. The relative levels were normalized to β-actin (*n* = 6). **e** Representative F4/80 stained kidney sections from UUO mice (left). Scale bars, 50 µm. The semiquantitative population of F4/80+ macrophages infiltrating the UUO kidney (right). Data are shown as mean ± SEM of 6 mice. **p-value < 0.01 versus the WT mice (*t*-test).

The pathophysiological function of HIF-1α CTAD was further evaluated in UUO, a nonreversible hypoxic renal injury model. Similar to the I/RI model, we observed that the expression of KIM-1 was significantly increased in UUO-induced kidney injury of HIF-1α CTAD^−/−^ mice (Fig. 2a, b). Histologically, severe necrosis and detachment of tubules, accumulation of cellular debris, and formation of casts were observed in HIF-1α CTAD^−/−^ kidneys with UUO (Fig. 2c). Meanwhile, the expression of inflammatory cytokine mRNAs (MCP-1, TNF-α, and IL-1β mRNA) was significantly increased in the obstructed kidneys of HIF-1α CTAD^−/−^ mice (Fig. 2d). In parallel, a significant increase in macrophage accumulation was also observed in that group (Fig. 2e). These results thus demonstrated that HIF-1α CTAD knockout enhances renal injury in hypoxic kidney models.

### Reduced HK2 expression are found in hypoxic kidneys of HIF-1α CTAD^−/−^ mice

**Fig. 3:**
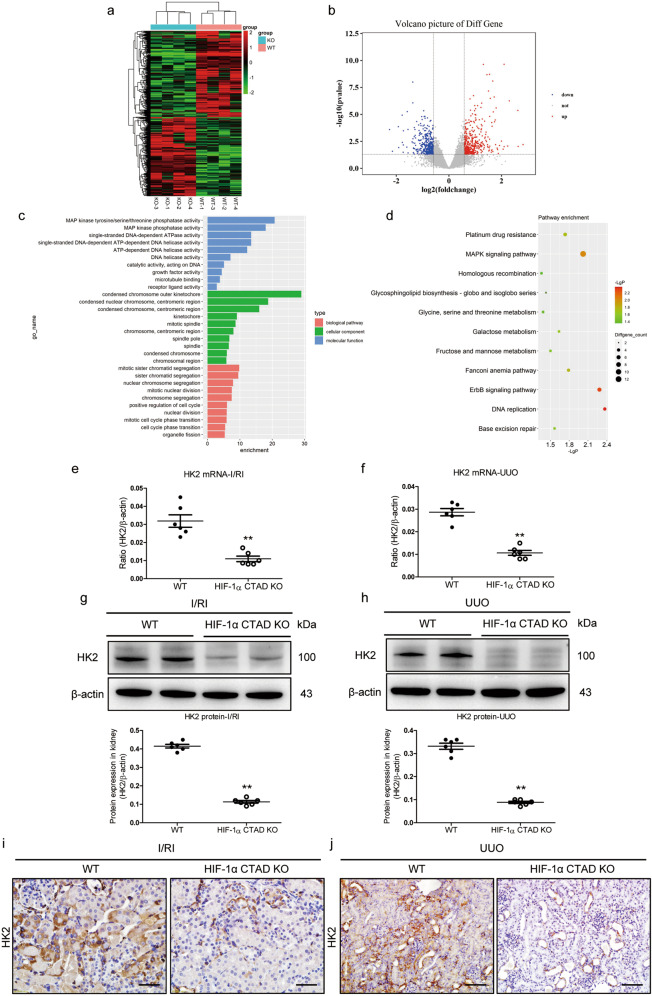
Reduced HK2 expression in HIF-1α CTAD^−/−^ mice is associated with hypoxia-induced kidney injury. **a** Hierarchical clustering shows distinct mRNA expression profiling between the HIF-1α CTAD KO and WT kidneys with I/RI. The red and green shades indicate the expression above and below the relative expression, respectively, across all samples. **b** Volcano plot showing differentially expressed genes. The up‐regulated genes (red), and the down‐regulated genes (green) with a fold change <0.6, and with *p* < 0.05. **c** The gene ontology category analysis of differentially expressed genes. **d** The pathway enrichment analysis of differentially expressed genes. **e**, **f** HK2 mRNA expression in the kidney with I/RI (**e**) or UUO (**f**) detected by qRT-PCR. The relative levels were normalized to β-actin (*n* = 6). **g**, **h** Western blotting analysis of HK2 expression in the kidney with I/RI (**g**) or UUO (**h**) (*n* = 6). **i**, **j** Immunohistochemical analysis of HK2 expression in the kidney with I/RI (**i**) or UUO (**j**) (*n* = 6). Scale bars, 50 µm for I/RI; 100 µm for UUO. Data are shown as mean ± SEM of 6 mice. **p-value < 0.01 versus the WT mice (*t*-test).

To investigate the exact mechanisms of potential detrimental effects induced by HIF-1α CTAD knockout, a genome-wide gene expression analysis was performed (Fig. 3a). The up-regulated and down-regulated genes displayed by volcano plot (Fig. 3b). To identify the inherent transcriptome features and predict the function of differentially expressed genes, we applied GO (Fig. 3c) and KEGG analyses (Fig. 3d) for feature selection. Interestingly, the differentially expressed genes were found to be associated with MAPK signaling, metabolism, and DNA replication. Considering the transcriptional function of HIF-1, we focused on the differentially expressed genes that were specifically down-regulated.

Intriguingly, we found that the expression of HK2, an essential glycolysis rate-limiting enzyme, appeared to be significantly down-regulated in HIF-1α CTAD^−/−^ mice (Fig. 3a). Therefore, the results of genome-wide sequencing analysis were validated both in vivo and in vitro. We found that the expression of HK2 at the transcriptional level was significantly decreased in the kidneys of HIF-1α CTAD^−/−^ mice with I/R (Fig. 3e) and UUO (Fig. 3f). Meanwhile, its protein expression was also confirmed by Western blotting (Fig. 3g, h). Additionally, immunohistochemistry analysis revealed that HK2 was expressed predominantly in tubules, however, HK2 expression was considerably decreased in HIF-1α CTAD^−/−^ mice with I/R (Fig. 3i) and UUO (Fig. 3j). These data indicated that HK2 is involved in renoprotective effects mediated by HIF-1α CTAD.

### HIF-1α CTAD transcriptionally regulates HK2 and attenuates hypoxia-induced tubule injury

**Fig. 4:**
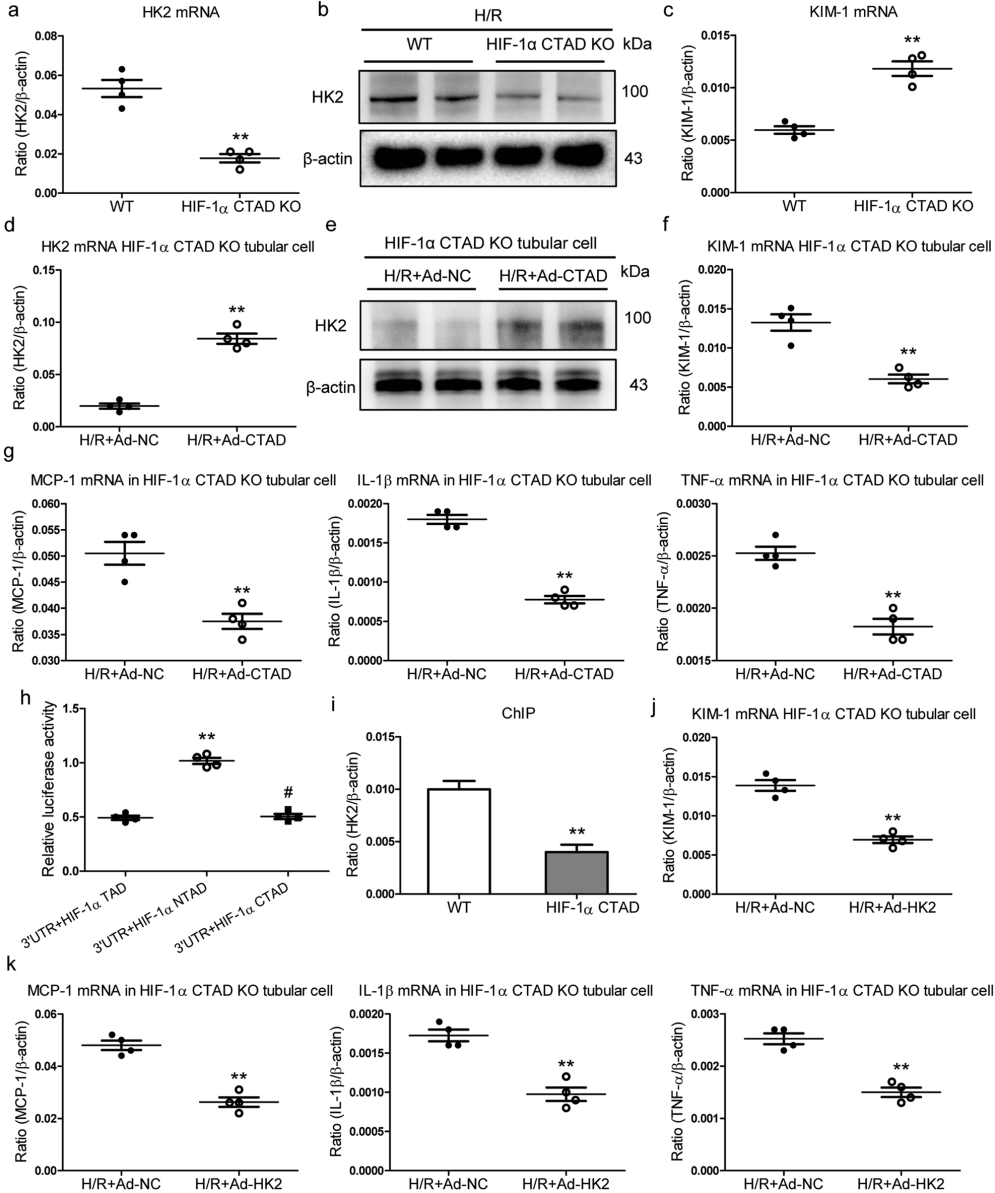
HK2 transcriptionally regulated by the HIF-1α C-TAD protects against hypoxia-induced tubule injury. **a**, **c** qRT-PCR analysis of HK2 and KIM-1 expression in the tubular cells with H/R. The relative level was normalized to β-actin (*n* = 4). **b** Western blotting analysis of HK2 expression in the tubular cells with H/R (*n* = 4). **d**, **f** HK2 and KIM-1 mRNA expression were analyzed in primary TECs with H/R before HIF-1α CTAD was overexpressed with adenovirus (Ad-CTAD). The relative level was normalized to β-actin (*n* = 4). **e** Western blotting analysis of HK2 expression in the primary TECs with H/R before HIF-1α CTAD was overexpressed with adenovirus (Ad-CTAD) (*n* = 4). g mRNA expression of inflammatory factors (MCP-1, TNF-α, and IL-1β) in the primary TECs with H/R after HIF-1α CTAD was overexpressed with adenovirus (Ad-CTAD). The relative levels were normalized to β-actin (*n* = 4). **h** Luciferase assay results corrected for transfection efficiency using β-galactosidase are shown as an average of 4 separate experiments ± SEM. **i** ChIP assay shows binding of HIF-1 to the HK2 promoter (*n* = 4). **j** KIM-1 mRNA expression was analyzed in primary TECs with H/R after HK2 was overexpressed with adenovirus (Ad-HK2). The relative level was normalized to β-actin (*n* = 4). **k** mRNA expression of inflammatory factors (MCP-1, TNF-α, and IL-1β) in the primary TECs with H/R after HK2 was overexpressed with adenovirus (Ad-HK2). The relative levels were normalized to β-actin (*n* = 4). **p-value < 0.01 versus the NC group (*t*-test or Mann–Whitney *U* test).

Considering HK2 was expressed predominantly in tubules, we subsequently explored its regulatory mechanism and function in TECs. We observed that the expression of HK2 mRNA (Fig. 4a) and protein (Fig. 4b and Supplementary Fig. S2a) was significantly reduced in hypoxia-treated TECs from HIF-1α CTAD^−/−^ mice. Meanwhile, a marked increase in the expression of KIM-1 (a sensitive and specific marker for tubule injury) was also observed (Fig. 4c). More intriguingly, we discovered that overexpression of HIF-1α CTAD significantly induced the expression of HK2 mRNA (Fig. 4d) and protein (Fig. 4e and Supplementary Fig. S2b) in TECs. As expected, KIM-1 significantly decreased (Fig. 4f). Concomitantly, decreased expression of inflammatory cytokine mRNAs (MCP-1, TNF-α, and IL-1β mRNA) was found in this group (Fig. 4g). These results suggested that HIF-1α CTAD knockout-induced HK2 inhibition might be an important mechanism for severe tubule injury.

Subsequently, the underlying mechanism of down-regulated HK2 induced by HIF-1α CTAD knockout was investigated. The in silico analysis was utilized to determine whether HK2 was directly stimulated by the HIF-1. Interestingly, the existence of a putative HIF-1 binding motif (5′-RCGTG-3′) located within the promoter region of the HK2 locus was found (Supplementary File 1), indicating that HK2 might be transcriptionally regulated by HIF-1α. According to the luciferase reporter assay, HIF-1α CTAD significantly induced HK2 activity in comparison to controls (Fig. 4h), indicating transcriptional regulation of HK2 by the HIF-1α CTAD. To establish the presence of HIF-1α CTAD at the promoter region of HK2 in vivo, ChIP assays were performed. As expected, we found that HIF-1α CTAD binding could be enriched on this promoter in the kidneys of WT mice but not in the kidneys of HIF-1α CTAD^−/−^ mice (Fig. 4i), confirming the direct interaction between HIF-1α and HK2 in response to hypoxic injury.

Thereafter, the potential function of HK2 in hypoxic TECs was also determined. The HK2 was overexpressed using adenovirus vectors. Interestingly, the significant decreasing expression of KIM-1 mRNA (Fig. 4j) and protein (Supplementary Fig. S3) was observed. Concomitantly, the expression of inflammatory cytokine mRNAs (MCP-1, TNF-α, and IL-1β mRNA) was also significantly attenuated when the HK2 was overexpressed (Fig. 4k). Taken together, HIF-1α CTAD-HK2 pathway plays a crucial role in protecting against hypoxia-induced tubule injury.

### Overexpression of HK2 protects against I/R-induced kidney injury in HIF-1α CTAD^−/−^ mice

**Fig. 5:**
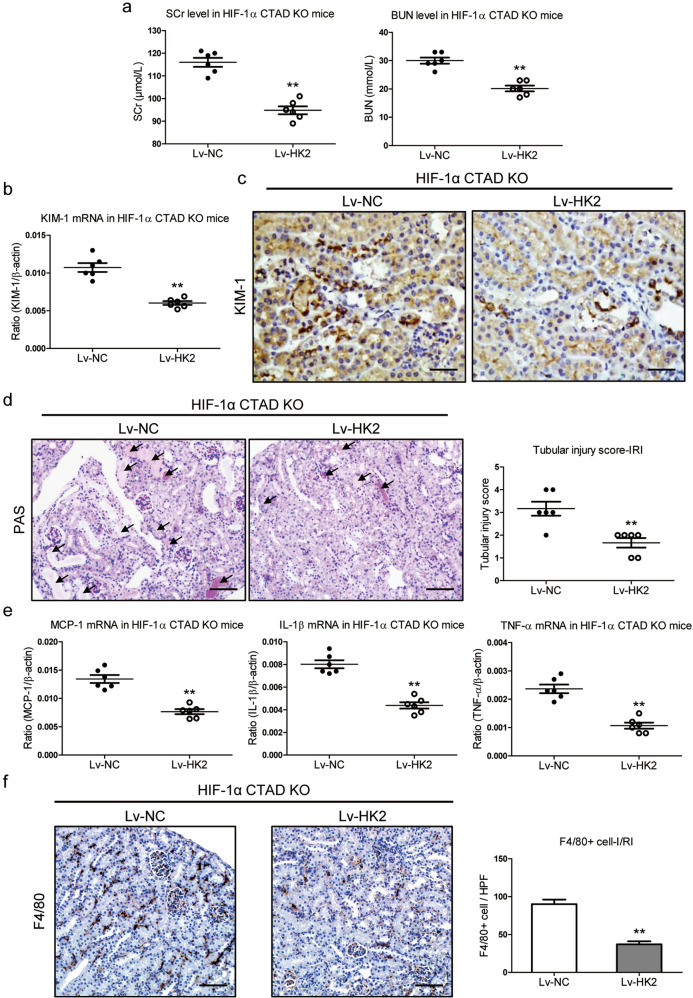
Overexpression of HK2 hinders I/R-induced kidney injury in HIF-1α C-TAD^−/−^ mice. **a** Scr and BUN level in HIF-1α CTAD^−/−^ mice subjected to 35 min of I/RI following Lv-HK2 administration (*n* = 6). **b** qRT-PCR analysis of KIM-1 expression in HIF-1α CTAD^−/−^ mice subjected to 35 min of I/RI following Lv-HK2 administration. The relative levels were normalized to β-actin (*n* = 6). **c** Representative images of KIM-1 expression in kidney tissues were assessed by immunohistochemistry (*n* = 6). Scale bars, 50 µm. **d** Representative histological changes (PAS staining) (left, *n* = 6). Scale bars, 100 µm. The quantification of tubular injury based on PAS staining (right, *n* = 6). **e** mRNA expression of inflammatory factors (MCP-1, TNF-α, and IL-1β) in kidney tissues from HIF-1α CTAD^−/−^ mice injected with Lv-HK2 (n = 6). **f**. Immunohistochemistry and semiquantitative population of F4/80+ macrophages infiltrating the kidneys (*n* = 6). Scale bars, 100 µm. **p-value < 0.01 versus Lv-NC. Data are presented as mean ± SEM of 6 mice. Lv-NC served as the control (*t*-test).

To understand the role of the HK2 in I/R-induced kidney injury under the conditions of HIF-1α C-TAD knockout, HK2 overexpression in HIF-1α C-TAD^−/−^ mice with I/RI was performed using Lv-HK2 injection. Strikingly, we found that the mice that received the Lv-HK2 injection had considerably reduced SCr and BUN levels (Fig. 5a). The expression of KIM-1 was reduced in the kidneys of that group (Figs. 5b and 5c). Meanwhile, periodic acid-Schiff (PAS) staining also demonstrated ameliorated kidney injury (Fig. 5d). Moreover, overexpression of HK2 reduced the expression of MCP-1, TNF-α, and IL-1β mRNAs in renal cortical tissues, as indicated by Western blotting (Fig. 5e). Furthermore, as illustrated in Fig. 5f, there was a notable decrease in the number of macrophage infiltration. These data clearly revealed that overexpression of HK2 could protect against I/R-induced kidney injury under conditions of HIF-1α CTAD knockout.

### HK2 overexpression prevents UUO-induced kidney injury in HIF-1α CTAD^−/−^ mice

**Fig. 6:**
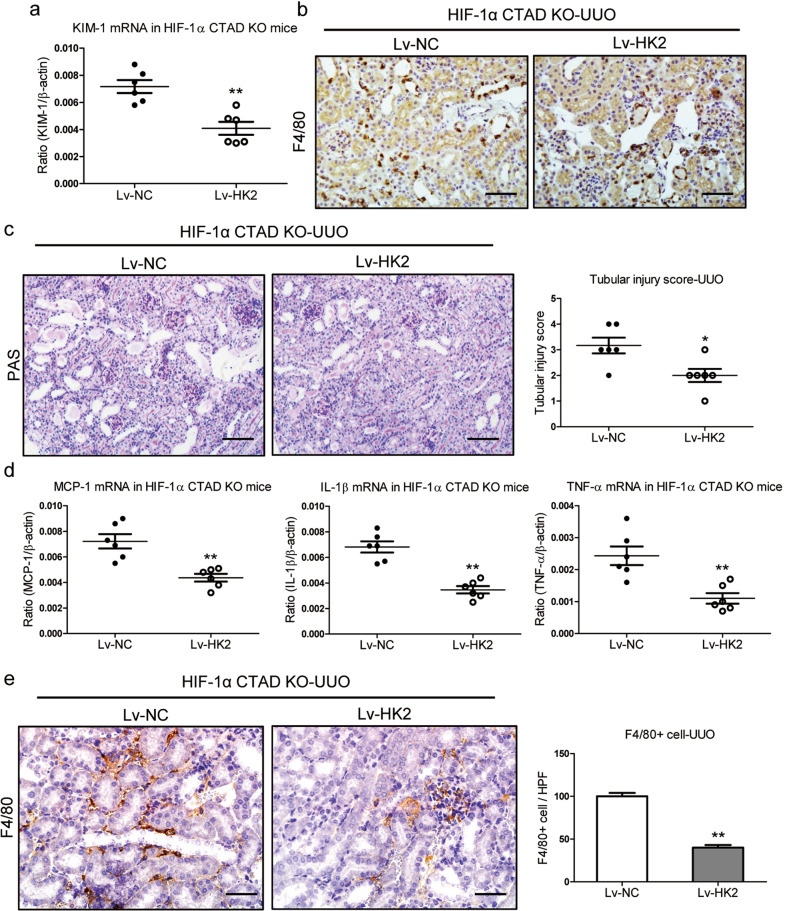
HK2 overexpression attenuates UUO-induced kidney injury in HIF-1α CTAD^−/−^ mice. **a** qRT-PCR analysis of KIM-1 expression in the UUO kidney of HIF-1α CTAD^−/−^ mice injected with Lv-HK2. The relative levels were normalized to β-actin (*n* = 6). b. Representative images of KIM-1 expression in kidney tissues were assessed by immunohistochemistry (*n* = 6). Scale bars, 50 µm. **c** Representative histological changes (PAS staining) (left, *n* = 6). Scale bars, 100 µm. The quantification of tubular injury based on PAS staining (right, *n* = 6). **d** mRNA expression of inflammatory factors (MCP-1, TNF-α, and IL-1β) in UUO kidney tissues from HIF-1α CTAD^−/−^ mice with Lv-HK2 administration (*n* = 6). **e** Immunohistochemistry and semiquantitative population of F4/80+ macrophages infiltrating the kidneys (*n* = 6). Scale bars, 50 µm. **p-value < 0.01 versus Lv-NC. Data are presented as mean ± SEM of 6 mice. Lv-NC served as the control (*t*-test).

The function of HK2 was also studied in UUO-induced kidney injury. As expected, KIM-1 expression was decreased in the kidneys of UUO mice with HK2 overexpression (Fig. 6a, b). PAS staining revealed ameliorated kidney injury (Fig. 6c). Meanwhile, the MCP-1, TNF-α, and IL-1β mRNA expressions were also down-regulated in renal cortical tissues of mice with HK2 overexpression (Fig. 6d). Furthermore, as illustrated in Fig. 6e, there was a considerable reduction in the number of infiltrating macrophages. Therefore, these data revealed that overexpression of HK2 could prevent I/R-induced kidney injury under conditions of HIF-1α CTAD knockout.

### HK2 deficiency-induced mitophagy inhibition promotes hypoxia-induced renal injury in HIF-1α CTAD^−/−^ mice

**Fig. 7:**
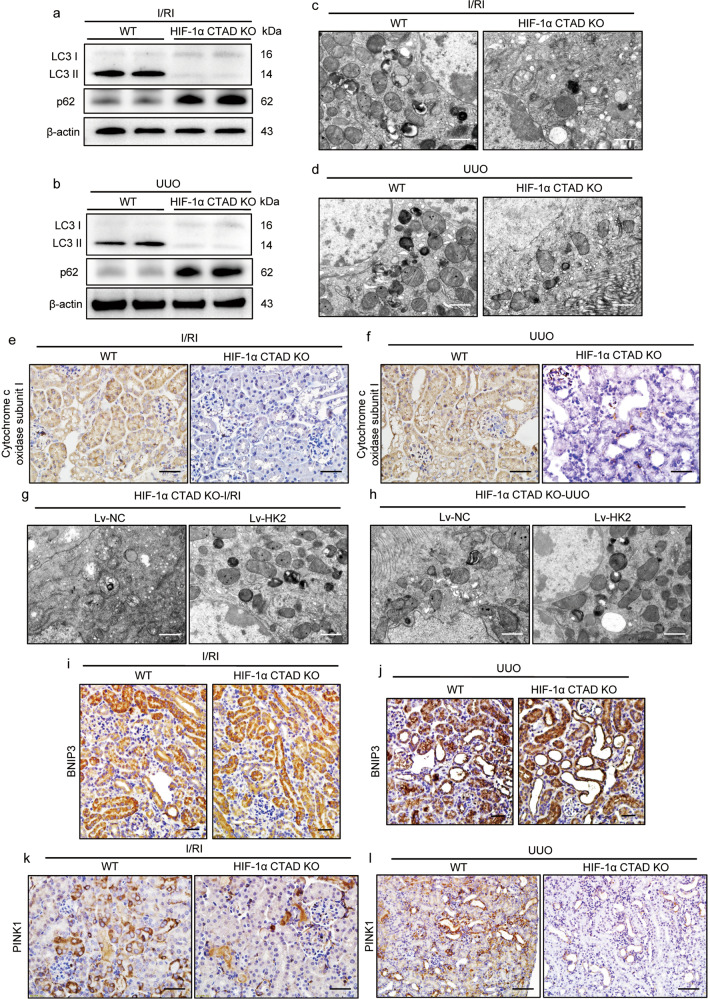
Mitophagy deficiency contributes to severe renal injury in HIF-1α CTAD^−/−^ mice. **a**, **b** Western blotting analysis of LC3 and p62 expression in the kidney with I/RI or UUO (*n* = 6). **c**, **d** Representative TEM images of mitochondria in renal tubules from mice with I/RI or UUO (*n* = 6). Scale bars, 1 µm. **e**, **f** Immunohistochemical analysis of cytochrome c oxidase subunit I (MT-CO1) expression in kidney sections (*n* = 6). Scale bars, 50 µm. **g**, **h** Representative TEM images of mitochondria in renal tubules from HIF-1α CTAD KO mice with I/RI or UUO following Lv-HK2 administration (*n* = 6). Scale bars, 1 µm. **i**, **j** Representative BNIP3 images of mitochondria in renal tubules from mice with I/RI or UUO (*n* = 6). Scale bars, 50 µm. **k**, **l** Representative PINK1 images of mitochondria in renal tubules from mice with I/RI or UUO (*n* = 6). Scale bars, 50 µm for I/RI; 100 µm for UUO. WT or Lv-NC group served as the control.

Next, the mechanism of HK2 deficiency-induced hypoxic kidney injury was further investigated under conditions of HIF-1α CTAD knockout. As HK2 is a crucial glycolytic enzyme in energy metabolism, we first detected the important glycolytic metabolites. Interestingly, we found that there was no difference in lactate and pyruvate levels between the two groups (Supplementary Fig. S4). A recent study has found that HK2 functions as a key regulator in linking metabolism and autophagy. Therefore, we proposed that HK2 deficiency-induced severe hypoxic kidney injury might be associated with defective mitophagy. Interestingly, a significant decrease in LC3-II expression was observed in the kidney cortex of HIF-1α CTAD^−/−^ mice with I/RI (Fig. 7a and Supplementary Fig. S5a) and UUO (Fig. 7b and Supplementary Fig. S5b), which was consistent with the increased expression of p62, which is a marker for impaired mitophagy. Meanwhile, HIF-1α CTAD knockout induced reduced levels of autophagosomes and autolysosomes in TECs in comparison to the WT group, as well as the number of autophagosomes/autolysosomes enclosing mitochondria, according to TEM analysis of kidney cortex of mice with I/RI (Fig. 7c) and UUO (Fig. 7d). In parallel, the reduction in cytochrome C oxidase subunit I expression (Fig. 7e, f) and mitochondrial respiratory chain complex I enzymatic activity was observed (Supplementary Fig. S6). Then, using lentivirus vectors, the HK2 expression was stimulated to determine the potential role of HK2 in mitophagy regulation. Interestingly, a remarkable decrease in LC3-II expression and an increase in p62 expression was attenuated in the kidney cortex subjected to the Lv-HK2 (Supplementary Fig. S7). As anticipated, the mitophagy changes were also confirmed by TEM, as shown in Fig. 7g, h.

Following that, the exact mechanism of HK2-mediated mitophagy was explored. It is well known that mitophagy is primarily achieved through the ubiquitin (Ub)-dependent (PINK1/parkin) or Ub-independent (HIF-1/BNIP3) initiating pathways. Therefore, the two pathways were detected in our study. Interestingly, we found that the level of BNIP3, a direct downstream target of HIF-1α, was unaffected when the HIF-1α CTAD was knockout (Fig. 7i, j). However, the expression of PINK1 was significantly decreased in the kidneys of HIF-1α CTAD^−/−^ mice with I/RI (Fig. 7k) and UUO (Fig. 7l). Meanwhile, the levels of PINK1 and BNIP3 in mitochondria were also examined. Consistently, the level of PINK1 decreased significantly in mitochondria isolated from the kidneys of HIF-1α CTAD^−/−^ mice with I/RI and UUO, whereas the level of BNIP3 was unaffected (Supplementary Fig. S8). Furthermore, to determine that PINK1 was regulated by HK2, PINK1 was detected when HK2 was overexpressed. Interestingly, a significant increase in PINK1 expression was observed (Supplementary Fig. S9). Collectively, these data indicated that HK2 deficiency-induced mitophagy inhibition promotes hypoxia-induced injury in TECs.

### Mitophagy activation protects against hypoxia-induced kidney injury in HIF-1α C-TAD^−/−^ mice

**Fig. 8:**
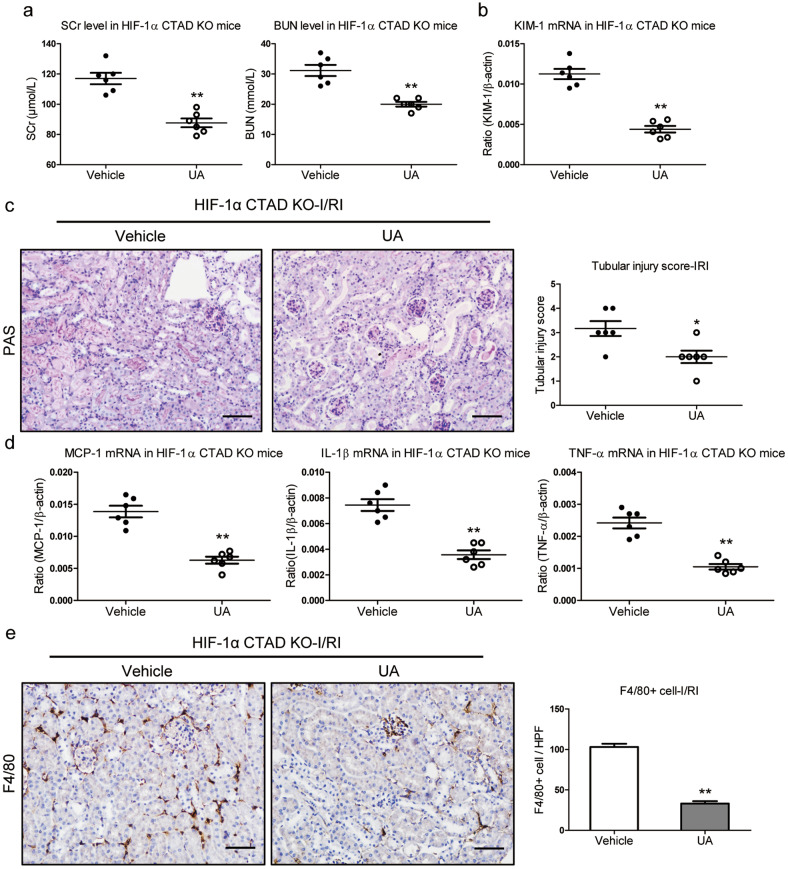
Mitophagy activation hinders hypoxia-induced kidney injury in HIF-1α C-TAD^−/−^ mice. **a** SCr and BUN levels in HIF-1α CTAD^−/−^ mice subjected to 35 min of I/RI with UA administration (*n* = 6). **b** qRT-PCR analysis of KIM-1 expression. The relative levels were normalized to β-actin (*n* = 6). **c** Representative histological changes (PAS staining) (left, *n* = 6). Scale bars, 100 µm. The quantification of tubular injury based on PAS staining (right, *n* = 6). **d** mRNA expression of inflammatory factors (MCP-1, TNF-α, and IL-1β) in kidney tissues from HIF-1α CTAD^−/−^ mice treated with UA (*n* = 6). **e** Immunohistochemistry and semiquantitative population of F4/80+ macrophages infiltrating the kidneys (*n* = 6). Scale bars, 100 µm. **p-value < 0.01 versus vehicle. Data are presented as mean ± SEM of 6 mice. The vehicle served as the control (*t*-test).

Urolithin A (UA), a specific mitophagy activator that has been proven to induce the improvement of mitochondrial health by stimulating mitophagy in humans [15], was used to activate mitophagy in HIF-1α CTAD^−/−^ mice with I/RI to confirm the functional effects of mitophagy on hypoxia-induced kidney injury in vivo. Firstly, the successful activation of mitophagy in tubular cells was confirmed (Supplementary Fig. S10). We found that the mice administered with UA had decreased levels of SCr and BUN (Fig. 8a). In consistent with the renal protective effect, the decreased expression of KIM-1 was also observed (Fig. 8b). Meanwhile, as illustrated in Fig. 8c, the necrosis and detachment of tubules, accumulation of cellular debris, and formation of casts were markedly attenuated in I/R-induced renal injury with UA treatment. Moreover, the expression of inflammatory cytokine mRNAs (TNF-α, IL-1β, and MCP-1 mRNA) was also down-regulated in that mice (Fig. 8d). Concomitantly, reduced macrophage infiltration was observed in injured kidneys treated with UA (Fig. 8e). These data indicated that mitophagy activation prevents hypoxia-induced kidney injury under the conditions of HIF-1α CTAD knockout.

## Discussion

Although HIF-1, a master transcription factor for cellular adaptations to hypoxia, plays a critical role in protection against hypoxia-induced kidney diseases [16], the precise effects of HIF-1α CTAD on hypoxic kidney diseases remain unclear. In this study, we first demonstrated that HIF-1α CTAD could exert a protective role against hypoxia-induced kidney diseases through transcriptional regulation of HK2. Moreover, we discovered that the HIF-1α CTAD-HK2 pathway prevents hypoxia-induced kidney injury by maintaining PINK1-mediated mitophagy, a previously unrecognized mechanism for responses of the kidney to hypoxia.

Hypoxia is one of the most important instigators in the development of kidney diseases and is closely associated with subsequent renal inflammation and fibrosis [1, 17]. HIF-1 was activated rapidly in response to hypoxia and determined the outcomes of hypoxia-induced kidney injury [18]. More interestingly, it has been demonstrated that the activation of tubular HIF-1 signaling through a genetic approach or pharmacological strategies could provide renoprotective [19–21]. However, increasing studies found that HIF-1 activation exhibits pleiotropic effects on kidney injury and repairs by regulating target genes directly [22]. Meanwhile, our earlier study also revealed the biphasic effects of HIF-1 on kidney diseases [23]. Recently, it has been recognized that there is physiological heterogeneity in the kidney-to-hypoxia signaling pathway, which might provide a novel understanding of kidney diseases [24]. Therefore, elucidating the exact pathogenesis of HIF in hypoxic kidney diseases is paramountly important.

In an earlier study, Chowdhury et al. [25] suggested that activating different domains of HIF-1α might finely regulate the functions of HIF-1. Structural biology studies have demonstrated that HIF-1α possesses two transcriptional activation domains (NTAD and CTAD), which could transcribe different genes, performing their own functions [6, 7, 26]. Recently, Ito et al. [27] indicated that activation of HIF-1α NTAD induced by PDH inhibitor protects the kidneys from ischemia through upregulation of glycogen storage. However, the functions of HIF-1α CTAD in kidney diseases remain uncertain. In the present study, the functions of HIF-1α CTAD in hypoxia-induced kidney diseases were investigated employing the HIF-1α CTAD^−/−^ mice. Interestingly, HIF-1α CTAD^−/−^ was found to exacerbate renal injury in mice models of I/R- and UUO-induced kidney injury. Furthermore, hypoxia-induced kidney injury was significantly attenuated by overexpression of HIF-1α CTAD. Therefore, activation of HIF-1α CTAD is one of the crucial responses of the kidney to hypoxia, and tubular HIF-1α CTAD plays a pivotal role in preventing hypoxia-induced kidney injury.

It was previously reported by Baumann et al. [14] that HIF-1α NTAD could transcriptionally regulate *COL1A2* by binding to a functional hypoxia-responsive element in its promoter to promote glomerulosclerosis. Meanwhile, convincing evidence has revealed that HIF CTAD knockout has no impact on the HIF-dependent transcriptional activation function [28]. Therefore, we hypothesize that HIF-1 CTAD plays a renoprotective role in kidney diseases by transcribing specific target genes. Interestingly, it was observed that HK2 was significantly decreased in the HIF-1α CTAD^−/−^ mice by mRNA sequencing. Furthermore, we found that HIF-1α CTAD regulates the transcriptional response of HK2, which plays a vital role in preventing hypoxia-induced kidney injury. Relevant to our findings, Chan et al. [7] also identified that HK2 is principally regulated by HIF-1α CTAD in hypoxic tumor cells. Consequently, the HIF-1α CTAD-HK2 pathway could be the critical molecular response of the kidney to hypoxic injury. To our knowledge, this is the first study to characterize the role of HIF-1α CTAD in the context of hypoxia-induced kidney injury, enriching the insight into the responses of the kidney to hypoxia.

Then, what is the exact mechanism underlying the aggravation of renal injury caused by HK2 deficiency? Considering that HK2 is the rate-limiting essential enzyme in the glycolytic pathway, which plays an extremely important role in energy metabolism [29], we hypothesized that HK2-mediated energy metabolism reprogramming might be the potential precise mechanism underlying HIF-1α CTAD-mediated renoprotection. Surprisingly, we found that HK2 deficiency resulting from HIF-1α CTAD knockout caused no changes in energy metabolism. Previous studies suggested that HK2 integrated glycolysis and autophagy to confer cellular protection in cardiomyocytes [30], and it is required for the recruitment of parkin to depolarized mitochondria [31]. Therefore, we hypothesized that HIF-1α CTAD-induced renoprotection might be mediated by mitophagy, which is crucial for the maintenance of kidney homeostasis [32]. Indeed, a significant reduction in mitophagy was observed in HIF-1α CTAD^−/−^ mice. Meanwhile, the finding was confirmed by administering a specific mitophagy activator. These were consistent with the results of earlier studies that activation of mitophagy prevents renal ischemic/hypoxic injury [33, 34]. Interestingly, Tan et al. [35] also found that HK2 could function as a sensor to trigger mitophagy in an acute myocardial I/R model. Thus, it is demonstrated that HK2 deficiency-mediated mitophagy inhibition is the critical mechanism in HIF-1α CTAD knockout-induced kidney injury.

In particular, HK2 is increasingly recognized as a component of a survival signaling nexus in addition to its fundamental role in energy metabolism, specifically glycolysis [36]. Here, we found that HK2-mediated mitophagy alleviates hypoxia-induced kidney injury, implying that HK2 confers cellular protection during the pathophysiology of hypoxic kidney diseases by integrating energy metabolism and mitophagy. However, it remains unclear what triggers the switch between the glycolytic and autophagic effects of HK2. Some evidence revealed that kinase-dead HK2 in endogenous HK2-depleted cells stimulates autophagy in the presence of glucose [37], indicating that the autophagic effect of HK2 is suppressed by its catalytic activity. However, further research is required to determine its exact mechanism.

**Fig. 9:**
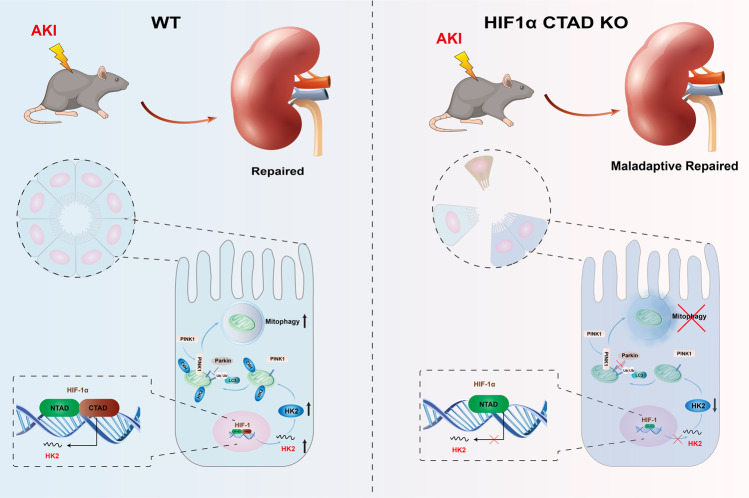
Schematic diagram showing the proposed molecular responses of HIF-1α CTAD signaling in hypoxic kidney injury. During hypoxia-induced kidney injury, tubular HIF-1α CTAD prevents kidney injury by activating HK2-mediated mitophagy.

Collectively, despite the considerable efforts of many dedicated researchers and physicians, the precise molecular responses of HIF-1 to hypoxia in kidney diseases remain to be elucidated. In this study, we demonstrated that tubular HIF-1α CTAD prevents hypoxia-induced kidney injury by transcriptionally upregulating HK2, which enhances mitophagy and subsequently plays a crucial role in renoprotection (Fig. 9). These findings suggest a new insight into the molecular mechanisms of the kidney to hypoxia, enriching the hypoxia response signaling and could provide an exciting intervention target for hypoxic kidney injury.

## Supplementary information

Supplementary table 1

Supplementary materials

Supplementary File 1

Original Data File

the Reproducibility checklist

## Data Availability

The authors confirm that the data supporting the conclusions in the paper are presented in the article and its Supplementary Material. Additional data related to this paper are available from the corresponding author.
