# Supplementary material for: Activation of HIF-1α C-terminal transactivation domain protects against hypoxia-induced kidney injury through hexokinase 2-mediated mitophagy
Source: Cell Death Dis. 2023 May 24;14(5):339. doi: 10.1038/s41419-023-05854-5 (PMC10209155; doi:10.1038/s41419-023-05854-5)
Supplement: Supplementary file 1 — Supplementary table 1 [file 41419_2023_5854_MOESM1_ESM.docx]

Table 1 Primers for quantitative Real Time-PCR

|  | Forward (5′–3′) | Reverse (5′–3′) |
| --- | --- | --- |
| Mouse IL-1β | TGCCACCTTTTGACAGTGATG | AAGGTCCACGGGAAAGACAC |
| Mouse TNF-α | TCTTCTCATTCCTGCTTGTGG | GGTCTGGGCCATAGAACTGA |
| Mouse MCP-1 | CATCCACGTGTTGGCTCA | GATCATCTTGCTGGTGAATGAGT |
| Mouse HK2 | TGATCGCCTGCTTATTCACGG | AACCGCCTAGAAATCTCCAGA |
| Mouse KIM-1 | CGGTACAACTTAAAGGGGCA | GACGTGTGGGAATCTCTGGT |
| Mouse β-actin | GAGACCTTCAACACCCCAGC | ATGTCACGCACGATTTCCC |
