# Supplementary material for: Activation of HIF-1α C-terminal transactivation domain protects against hypoxia-induced kidney injury through hexokinase 2-mediated mitophagy
Source: Cell Death Dis. 2023 May 24;14(5):339. doi: 10.1038/s41419-023-05854-5 (PMC10209155; doi:10.1038/s41419-023-05854-5)
Supplement: Supplementary file 2 — Supplementary materials [file 41419_2023_5854_MOESM2_ESM.pdf]

Supplementary figure 1

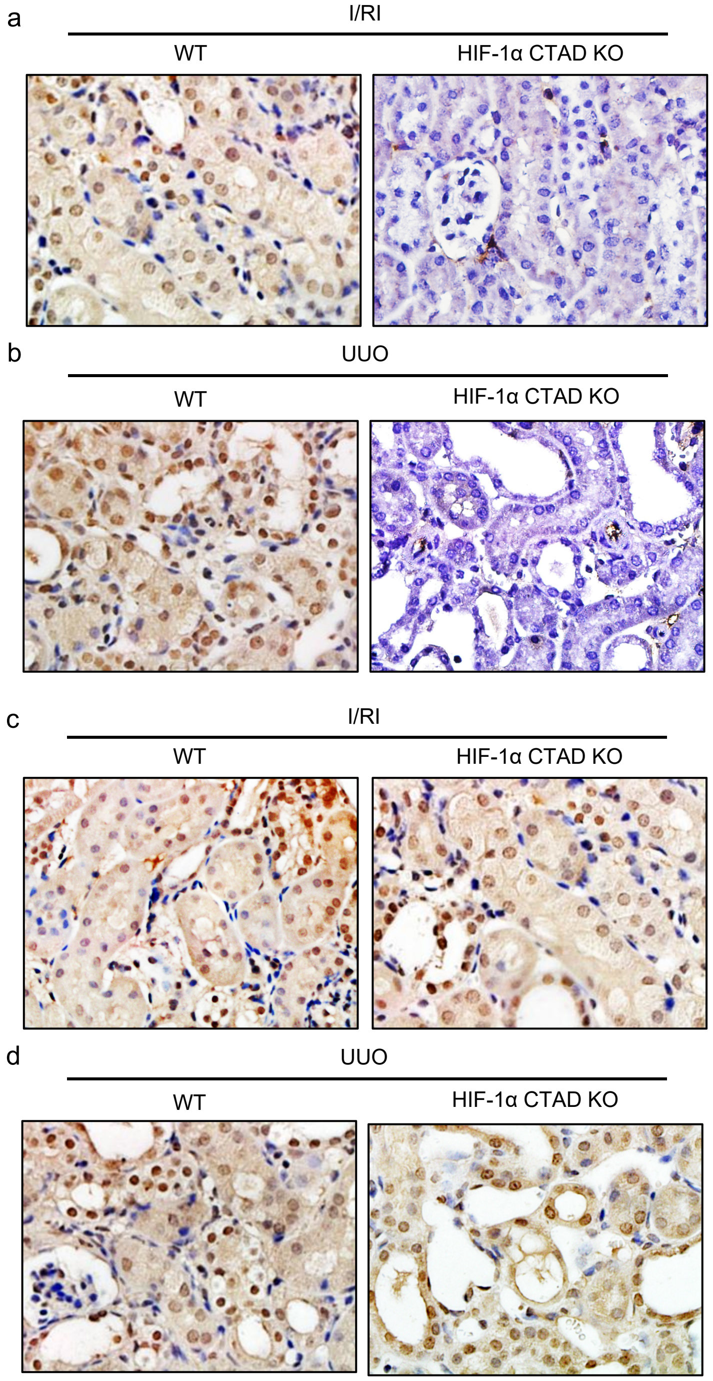

Figure 1. HIF-1α CTAD deficiency results from HIF-1α CTAD knockout. a, b. Immunohistochemical analysis of HIF-1α CTAD expression in the kidney with I/RI or UUO (n = 6). Scale bars, 50 µm. c, d. Immunohistochemical analysis of HIF-1α expression in the kidney with I/RI or UUO (n = 6).

Supplementary figure 2

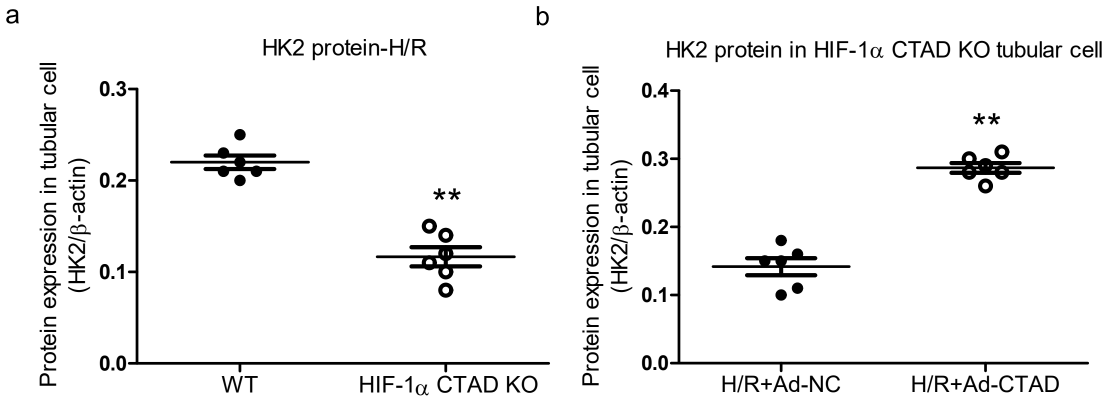

Figure 2. The quantification of target proteins expression are shown. a. The quantification of HK2 proteins expression in hypoxia-treated TECs (n = 6). b. The quantification of HK2 proteins expression in TECs with overexpression of HIF-1α CTAD (n = 6). **p-value < 0.01. Data are presented as mean ± SEM (t-test).

Supplementary figure 3

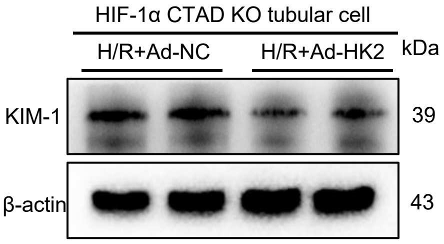

Figure 3. HK2 protects against hypoxia-induced TECs injury. KIM-1 protein expression was analyzed in primary TECs with H/R after HK2 was overexpressed with Ad-HK2 (n = 4).

Supplementary figure 4

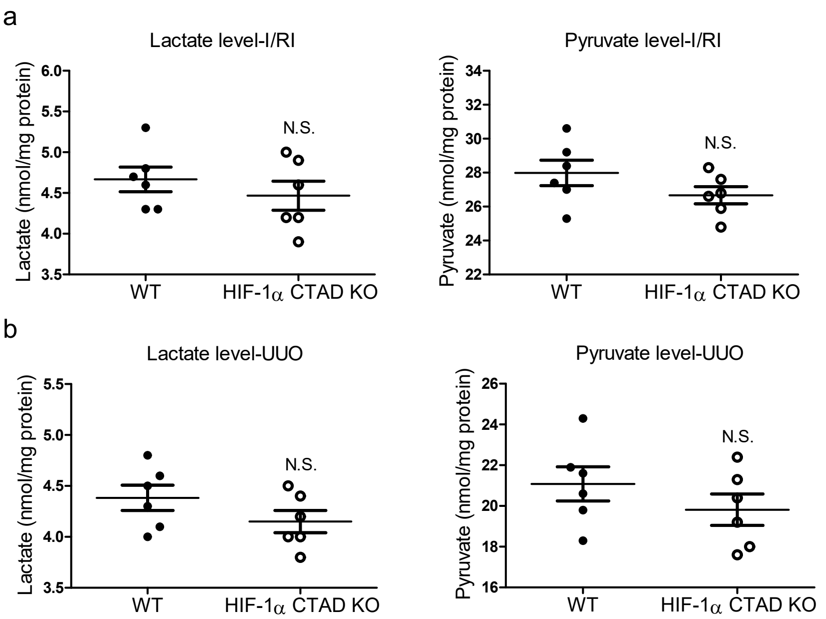

Figure 4. Glycolytic metabolism was not affected when the HIF-1α CTAD was knocked out. a-d. Lactate and pyruvate levels in the kidney with I/RI or in the UUO kidney (n = 6). **p-value < 0.01 versus WT. Data are presented as mean ± SEM of 6 mice. WT served as the control (*t*-test).

Supplementary figure 5

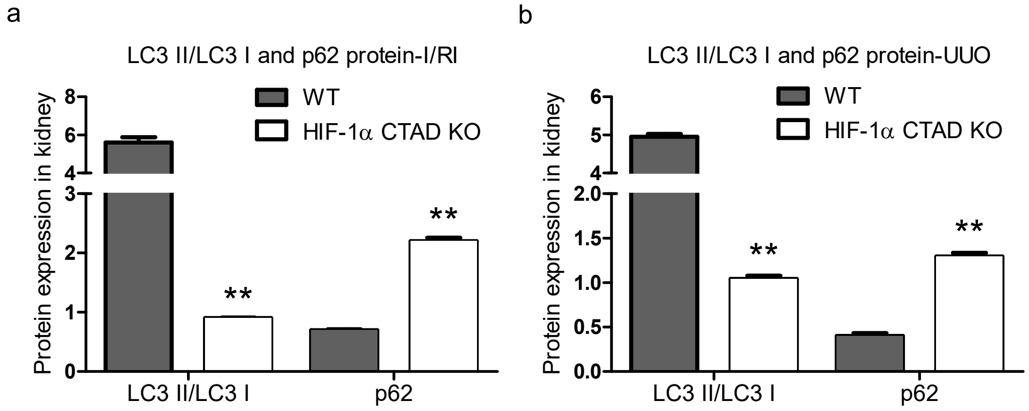

Figure 5. The quantification of target proteins expression are shown. a. The quantification of LC3 and p62 proteins expression in kidney with I/RI (n = 6). b. The quantification of LC3 and p62 proteins expression in kidney with UUO (n = 6). WT served as the control (*t*-test).

Supplementary figure 6

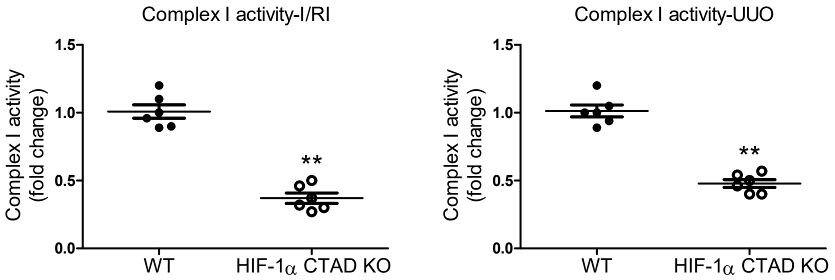

Figure 6. HIF-1α CTAD knockout results in the reduction in mitochondrial respiratory chain complex I enzymatic activity. Mitochondrial respiratory chain complex I enzymatic activity (n = 6). **p-value < 0.01 versus WT. Data are presented as mean ± SEM of 6 mice. WT served as the control (*t*-test).

Supplementary figure 7

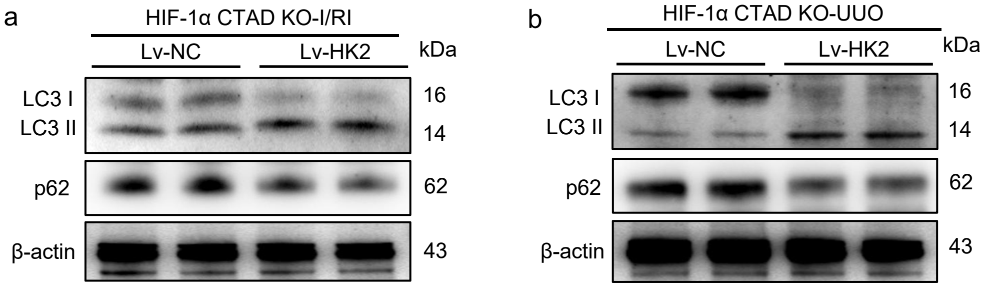

Figure 7. HK2 mediated mitophagy. Western blotting analysis of LC3 and p62 expression in the kidney with I/RI (a) or UUO (b) from HIF-1α CTAD$^{-/-}$ mice injected with Lv-HK2 (n = 6).

Supplementary figure 8

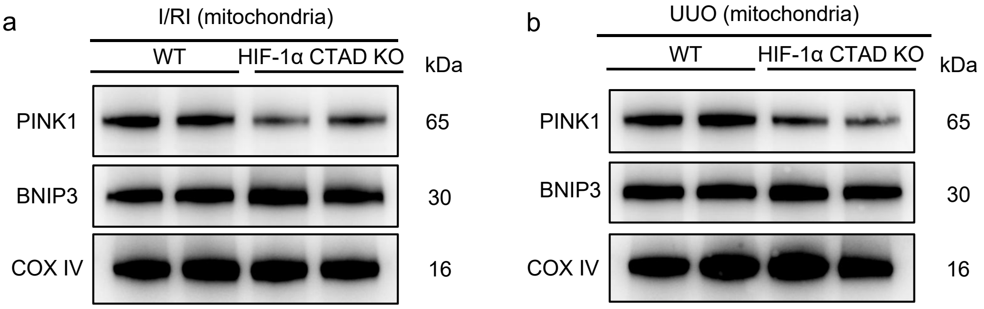

Figure 8. Mitochondrial PINK1, not BNIP3, was associated with kidney injury. Western blotting analysis of PINK1 and BNIP3 in mitochondrial isolated from the kidneys of HIF-1α $CTAD^{-/-}$ mice with I/RI (a) or UUO (b) (n = 6). The relative level was normalized to COX IV.

Supplementary figure 9

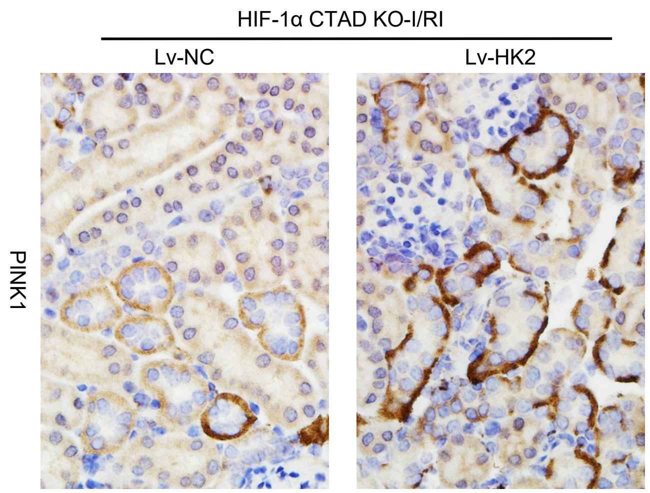

Figure 9. HK2-mediated mitophagy was associated with PINK1. Immunohistochemical analysis of PINK1 expression in the I/RI kidney from HIF-1α $CTAD^{-/-}$ mice injected with Lv-HK2 (n = 6).

Supplementary figure 10

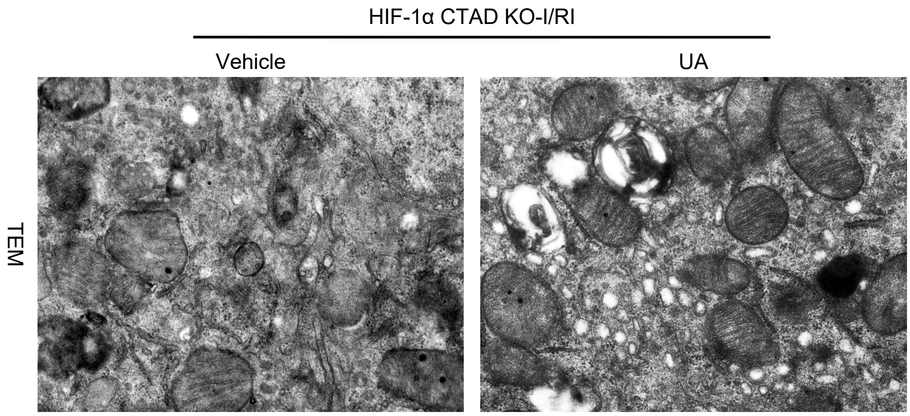

Figure 10. Successful activation of mitophagy by UA administration in tubular cells was confirmed. Representative TEM images of mitochondria/mitophagy in renal tubules from HIF-1α CTAD KO mice with I/RI following UA administration (n = 6).
