# Supplementary material for: Activation of HIF-1α C-terminal transactivation domain protects against hypoxia-induced kidney injury through hexokinase 2-mediated mitophagy
Source: Cell Death Dis. 2023 May 24;14(5):339. doi: 10.1038/s41419-023-05854-5 (PMC10209155; doi:10.1038/s41419-023-05854-5)
Supplement: Supplementary file 3 — Supplementary File 1 [file 41419_2023_5854_MOESM3_ESM.docx]

Mouse (mm10) chr6:82725027-82774454

ttgaaaaaaaaaagttaaaatttattaataataGTTAACATCACATGGTTAATTCAACTAGTTCTCTACTGTACATGATGACAAAACGCTCACTAGACCGAGTGCCCAGGGATATGAGATGAGTCAAAGGTTCACTGAAGATCACACAAAGCAGCATTAGCAGAGAGAGCTGTCAACCTAGTGCTGGCAGTAGCCAGCCTAGTACTAGGAACACAGCACACTGTCTCATGCTCTGGATTGAAAGCCAACTTCCTTAAAAGTTAGacacacacacacacacacacacatacacacacacCCCTTTTTAAAAAAAAGTCCTTTCCTAGATGGTGCTTGATTTTAAAGTTTAAAATTCAAGATTCGAAAATTTAGTCAGAATCCATTTGTACTCCGTGGCTACCCTCTAGTGCTGCACACATCTATAGGTGGCTACCCCCTAGTGCTGCACACATCTATAGGTGGCTTCGGCTTTCAGTGGTCTGCTTCGGTAACTGCAGGTTGAGGGAGCTACACCAGACAGGCAGTATCAGGCAGGAATCACTCGACCTTCCCTCTGGGCATCCCGGGAGGACCTGGGCAGCCTTCATTTCACTGTACACCAGCTTGTTCGGTTGTAACTAGGCTCTGCTCAGCACAAGTCTGAAAGATGTTCAAAACATGGCTGCCACGATGCTGAGCTTTCTCTTGGTCTTTCTCTTTTGACCCCCGTATCTTGTTTATTAGTGTTACAAGGCTGTTTCCTCTTTATTAAACCTATCCAGGTTTAACATTGGTGGTCAATTTAATATTTTGAAGGGAGAAGTGAACTTTTGAATTCAGAGCGAACCGTGTTGGTAGAGCAGAGAACAGGACATTGGTGTCTTCCCGTTCTTCAAACAGTCTGGAATAAATAACTCGTATAAAAAGGCTAGTCGGCAGCTCGATGCAGCTCAGTCCAAGGAAGCAGACAGACAGCGTGCATCTCGTGTAAAACACGAGACACACCGGAGGTGGGATCTGCTAGGAGGGCAAATAAATGTACAAACACCCCGAGACGCCCCTGAGGTTTGGTCGAGTTCTCTCGATGCTTCATGTGGTCTGCTCCAACGATCTGATGTGACTTTAAAAACATTTTCGCTGGCTTCCTGTAACTTGTCAAACCATTCTGAAACGCCGACTGACCTAAAAATCACATCTTCTATGCAGTGAGAACTTGAAACAAATCCTTTGGTTTTCTTTGCCTAACATCCCGGGTGAGACCTCACTCAGGAAAGCTTCTGGACGGAGCAGCCAGGTGTTGTGGCTTCTTTCCCTCAGCGAACCACCCTGTCTCCATCCCTGCCTCGCATACACCTCACGGAGCAGAGGAAGGTGAAGCCCGGAGGCTGGTGGCGCTCTGCCCATCACTCACCAAGAGATTCCGAGTCTCTCTTCTTCCCGCGGGTGAATGAGGTATTTCTAGAGCCAGTCAGTGTCTCCACCTCATATCCTGAACTACAATGATCTATCTACTCACAAGGCCGAGTCAGAGGAGACCCTGGCCAGCTATATACAGAACAAAGGAACATATAAATACATTACTTACATAATTAACCATTACACAATGAAGGAGAGGACCTGCTTCAAACtaaataaatttcaaacataaattaaaaaaaaaaaaaaaaaaGAAATTGAAAAGCTACCAAAGCCACCCCTAGCCCCAAGGTCTGCTGCTCATAGATGACCCGTTCAGCCGTGTGAGGTAAAGCCACAGACACTTGAAGGGGGTGTCGAGTCTAGCTTTGGTTTGGGTTCCTCTATTCCAGGACAGCAAATGTTTAGAACCAGCAGGGTGATGCCTACAGGTTGAGCTGAAGAGGGACAGCCATGTAGGCCAGGGGACGTTCCATACTGctcctcctcctcctcctcttcctcctcctcctccCTCCCTCCCAATGCCTGATCTGATGACAGAAGTATCAACATTTCAAGATGTTCTCCACCTATAAATGTCGCATTCAGCTCCAAGATGAGGCAACCATATTCGCAAGACTGTCCCCATCCCATAGGACTGTACAGGAAGTAGAACAGGGGAGGGAACATGCCAGGAAGTGACCAGCAGGAACAACTTGTGAACAATTCCTTACTTCTGATTCTCAGGAAGGCCAAGGATTGGGTATCAAACTCTACAGTGAATATCGGGACAAAAGTCCCAGTCTCTCGGGGTCCTCTCTGCCAAGGAAATGCCAAGAGGTCCATCTCCTCATAATTTAAAACAGGGAAGTGAGGAAGCAGGGAAGTCCCGTCTGCTCCAAGGTTCTATCTCTGCCCGGCCTCCCGGATGCGACAGGCCACAGCAGTGATGAGAGCCGCTCCCTTCCCACTGCCGTCCTCGGATTCCAGGAAGGACACGTCACATTTCGGAGCCAGATCTCTCACCGTCTCATGCATGACTTTGGCAAAGCTGGAAAGATAGAAAAAGGGGATGTGAGTGGCTGGTGTCCTCAAGCAAAGCTTGGAATGAGGAATGAGACGTGGAGCACGGCACCTACAAACTAACAAACAGACAGGCTTGCAGGGGCTAGGACCACACCCAAGCCTTATACCACACCACAGTGGCCAATCGCAAGAGTAGAAACCTCAAGTTTTGGCACAAATGTCTTGAGATGCTGGTGAATTTTCTTCAAAACAAATCAGTTggtctggggatgtacctcagttgataatacttgcctagcagcataagtgaggaccttgggttcaccccccgccctgccctcccgccccagcatctaccgagcaccgtagcccacccatgtctgtaatcccagcaaacaagaagcaggggaatcaaaagctcaaggttacccttggctacatagcaaggatgtgctatgttgttaaaggccagtgtgggatacaagagactctagtttaaaaaacaaaGCAAggggtgggatagatggctcaacaccggctcaacaccgagaagtacaaaaccccttccttgcaggggaccaagattcagttgccagcccctacatggtgttcacacaaacaatgttcacaataccctgcaactccagctccagaaaacccaatgccatcttctgccctccgtaggcacTGTACCCGTGTCCACGACTTCCCTACCCCCCCACACCTCTACCAAATAATTCAAGTAACCTTTAAAGGGAAACTTTgggctagagagatggctctgcggttaagagcatctattgttctttcaggagccaagtttgatcgccagtaacaggtcaggctgctcacaactgaccagtgccaggcaatcAGGCTGCAAGGGTACCATCACTCTTGTGTAATACCCACCACAGAGACGAAAACATATCAAAACGACTAGGCATCACATCATTACAGAAACATCAGTGATACGATCACTGAGAAATACGATCTCTGGTAGTAGTGAACTCCCGTGGTCCCACCCCAAGAGACAGATGATTTGGAATCAGCTACATAATGAATTCAAGGCTATCTTGGCTCAAAACCAAAAGAGTTGTGATCCAGCCTTGAAATTAGCTAACTACACCTGCACAGAAACTCTCAAGCCAGCAAAAGGGTCATCACCACTAATCTAGAAACTAAGCACTAAACCCTCTTTGGAGTGGCTGAGAGAAGGGCGAGAAGAGAAATATACAAGGAGAAGAAGGAAGACAGGACAGCCCCAAGGTCAGGCACTCACTGAGGATGAAGCTTGTACAGTGTCCCGTCCACGCCCACCGTCACTTTGAGGTTGTCCAGTCCACGGTTCTCTCTTATCTTGTCCACCACGGCGGCCATGCCTGCGCCACAGAGCTGGGCCGCGCGCCGGGCGACCACAGTGCACACCTCCTTCACAATGATGCTGTCGTCACACGTGCTCTCCAGCCCTAGGTGGCGCAAGATGGCTCGGACCTGTAGCAGGGCCAGGCAGTCACTGCGTGGGAGATGGACAGTGCACGGTGAATAATGCGCAGAGAGGCTTAGTGGCCTGCAATCCCTAAGGCCCCTCTGGTTCCACGGGGGTCAGCAgggcaggggtgggggtggggcgggagtggggttggggagggggTCTCATTCAGTTGCTCTTCCTACAAACTACTTCCTACTGAGCTACAAACAAGCGGCCCTTCATTCAATTTTACATCCGAGGTAGGGCATTGTTACTACAGGCTGAGGCTCAcaggcatggtgagtcagtcactctgtaatctcagctgtcaggagcctcaagcaggaagattgcaaccaattcaagggcaaccttagttagctacacagtgagacccgcctcaagaaagccaaCACCAAACTATACTGAAAACAAAAGACAATCCACATCCTACATGGAGACCACCCCCATCCTCAGAGTGGGTCTTTGGGGAGTGTCAGTTAGGAATGGGTCACTCACTAGGGACAGACCTGCATCTGCATCCTGGCTTCAGAGGAGCTGCAGAAACCCTTCAGCTTGCCTACTCATTATAGAAGAATAAAGGCCATGGCTCACCTCCCTCCCAAGCCTCTCATCCTTACCTCTCGATCTGAGACAGGAACTTAGTTTCGAAGATTCCCCTTGTCTTGAGGCGCTCTGAGATGCGGCCACGGAAGAGCAGCCCCCGCTTTGTGAAATCGATCAGGATGTTGCGCACAATCTCGCCCAAGTACATGCCGCTGATCATCTTCTCAAACCTGCAACGGGACGTTGTCCACGGAGGTTATGTCCAGGCTGGGGGCGGGGACTTTGCAGGGGTGGGGGGAGGGGCCGGGAGAGTCATGCAGGCAACATTCAAGAATCTTCTCAATCATTCAGCAAAACTCACCACCTGATATCTGAGGCCCACAAGGCTTAATGTTAGGAAAacacacacacacacagagacacacacacaACCAATTCTAGGAGCAGGGGAGGATAACTGAGTCTTCCTTAATTCCTTAACTCCACTGGGCAGCTTACGTTTTCTCTTGGACCTTAAGTAACAACAGCAGAAcccacccccaccccccacccccacccaccatcctcacTTCTGGAACCAACCATGACTATGACATAACCTGGAGACCTCTGGAATACGCACAATGCCTTTTTGCAAAGCCAAGGGTCCAAGGTGCACTGAAGACACTGTAATTGTCCTGCCTCTTTTTGTAGCCTGCTTCCTTCATTAATGCTGCCTGCCTGACTTGCAGAGGTATCTCAGTCTGGGAGATATTCTCTAATTAAGAATAATCATAGACTGCCAAGTGTATTCTGAAAATCCATGTCTTTGGCTACAAACTCAGTCTGGTAACACTAGTATAGCATCTTAAACCTTCCTACCAGGCAGATCTTAGGTCTCTGTTCAGCTCCAAGCCCCTCCGCTGTATGAGGCTCTGTAACACTCGGATGCTTGTCCCTTCTGACGATAGAGATGCTGGATGCTGAGTAACCAGAAAGGGTTCCCTCCTGTCAGAGCCACAACAGTGCTTCTTCACACTGGGAGGGgtgtgtgtgtgtgtgtgtgtgtgtgtgtgtgtgttgtgtgtgtgtgtgtgtgtgtgtgtTTCCAGTTCCCACCCGGTTTTCCTCAATATCAGACCTAGCTTCTAATACACTCTCCAACACCTTGAGGCATGTTTAAGAAGACCCTCTTTGAAGGAGAAGCCCATTAGGCTCCTAAGAGAACAGGCATTCTGAGGAAAGCCAAGGTGACGGAGAGCCCAGTCAACTGAAGTCCCGTTCTGGGACTTAGCTCCAAACACAGGTGATTGCTTACCTCTGTTTGCCAGGGTTGAGAGAGAGCTCGTCCACAGCGACGTCAAACACTGTGCGCAGGTCATCCAGGCAGCCGTTGTCCCCGAACGCTCCCCACTCCATGTTGACACACATCCGCCCCTCCTCTCCGTCCACCAGTTCCACATTACGCATCTCCTCCATGTAGCAGGCATTGCTTCCAGTGCCTGTCAATCGGAGGCCACCAATGCTCATTTAACACCAGCCTTCCACCCATCCAGAGCCATGCAAGCCCACCAGGGGAGGATCAATAGTCTCCTTACCAACAATGAGGCCAACTTCACAGTGAGGGTCTTCATAGCCACAAGTCATCATAGTCCCAACTGTGTCATTTACCACGGCCACCACATCCAGGTCAAACTCCTGATGGCAAGAGGAAGATGTCTACTCCACAAGTCCCAATCTTCAAGACACATACGCGCACATGCAGGTGCAGACACACACGCATCCCCAAATGGTACATGCCGTGGCCCGTTCCTCATTTCACCTGCCCCAGCTCAGGCTGAGACCTACACTTCCACTGGCCAGGACATTAAGATCACCACTTCCgagcagagaggacagatcagtcggcagagaacagtgtttgctttccttacaagcatgacaacccgagtttgatcttcagaacccataacaagagacagacatggtcatgcatgcttgcaatccctgcactggggaaatggagaccaagagactcctggggcttgctggctagccaacctagtctaaccattccatcccaggccaatgagagaatctgtctcaaaaaccaagataagtggggggctggagagatggctcagaagtgaagagcactgactgttctggtcctgagttcaattcccagcaaccacacatggtggcttacaaccatctgtaatgagatctgattccctcttctggtgggtctgaggatagctacagagtactcatataaataaaataaatcttcaaacaaacaaacaaacaaaACAAAAACagccaagcatggtggcatatacctttgattccagcctttcagaggctgaggaagTCCCTAAGGACTACCATCTAAGGCTGCCCTCTGGCTacacacacacacacactggctacacacacacacacacGCTGCCACTTATCTGCTCACCCTTCTTTGCTCTTCCCCATTCCTCAAAGCTCTTACAGCAGCACCTGCCGCTGGAGCATGGCCTTCTTCTAGCAGTTAACCAACCACCGCTGAGTGATAAAGTTATTTAACCCCAACAAAGCAGTAACACGTGGTAATCCTCCCAGATTCCAAGATACACCAAGCCTTGCACCACGCCTCCCACCTCTCGCCGGCGAATGGCTTCCTTCAGCAAGGTGACCACATCTTCACCCTCGCAGCCGGATGCCTTGAATCCCTTTGTCCACTTGAGGAGGATGCTCTGAAAATAAATAAAACAGAAAAAAATAAAAATCACACAAAGGAGGAACATCCACCCTGGGTCATCCCTGGAGCCTGGCTCTTCTGCGTCTAGCTCTCAGCCTCGCAGAGCAGATTACCCACAAATGATCCTGAGCCTGCATCTAAGGTACTACTTACTGACTTGGttacatcacttgatcctcaccaccacttgtgagagctttatcatgatcacagttacttggtgggaaatcaaggcactgaagggatttgcccaaggtcTAGATTACACCCTGGCCCAGTGACTCATGGCTCAAGCACCATGTAAATGCTCCAACAATTATTTCCTTCAGCGGGCCAGAGTGTATGCCTTAATTCCATAGTTCAATGAACAGTGGTCTCGGTGACAACCTTGAAAGGTCCTCGGAgctaaacacatgaaactcaaggagaatgaagactgaagtgtggacactatgcccctccttagatttgggaacaaaacacccatggaaggagttacagagacggagtttggagctgagatgaaaggatggaccatgtagagactgccatagccagggatccaccccataatcagcatccaaacgctgacaccattgcatacactagcaagattttattgaaaggacgcagatgtagctgtctcttgtgagactatgccggggcccagcaaacacagaagtggatgctcacagtcagctaatggatggatcatagggctcccaatggaggagctagagaaagtagccaaggagctaaagggatctgcaaccctataggtggaacaacattatgagctaaccagtaccccggagctcttgactctagctgcatatatatcaaaagatggcctagtcggccatcactggaaagagaggcccattggacttgcaaactttatatgccccagtacaggggaataccagggccaaaaagggggagtgggtgggcaggggagtgggggtgggtggatatgggggacttttggtatagcattggaaatgtaaatgagttaaatacctaataaaaaatggaaaaaaaaaAAAAAAAAAAAAAGAAAAGAAAGGTCCTCGGAGTCTTCTACAGACCCTgagagggggaagagaagggaaaggggttggaggggggggggggggcagggTTTCTGTTCACATCTGCGCACATGCACAAAACTGTGCACATGGCTtgtgtgtggaggccagaggctgatgtcacacaccttccccatttgctgtcctctgtattctttgagggaatctgccattatatctggaactcactgagttaagcctggctggcagggagcacccaggacctgcctgcctgactctccttccgcagtgctggtgttccaggcacagcaccgcacccagcttttatatgggtgctggagatctgagcccagatcctcattattacaaagcaatcaatttaccatctgagccagccccctgcgccccGCTCTAGGGCATCCTCCCCTATGTGGTGGCACATCTAGGCACAGGTGCCCATTTGCTTTGTACATGGGAGAAAGAGGGTTTGTTCCTTGTTGCTCTTGATACGATAGCAGCCAGGACAAACCAGTATCCTCAGCTCCCTTTCACAAAAGGCTGGAATCGAGACCACCCACATGCATGGTGTCTCCAAGTCTTGCTCATGGTATATGCCCATCACCTGGGTATCCCATAAACCTGGGTGTGTCCCACTGTGGTGAGAGATCCCGAGAGATTTCGAACAAAACACAGCAGCACGGAGGCCTGGCTAATTACACCCACACAGCGACAAAGGCAGGCTTGTCTCTTCTACTCCTCTCTCATCTACTGCTAAGCCCTGCGCCTCTGGCAAAAGTGTTTATAACCTCATTCCAGATGATCACTATAACAAGACAACACTGGGCTTCAACAACCAGAGACTTGCCCAAGGCCAGTGGTGAATAAATGTGGCCAAAGACCTCTGGGCCTCCAGGTGTCCTTAACACCCACTGAAACCTCATCAAAAAGGTGATGTCACCAGAGCCAGGGCCACTGCTGGCTCCTGCTTTGCTTACTCTTTATACCAAACTGCACATGTTGGCATGAAAAGAGAGGGTCTACTCATGTGCACAAGGACCTCCATTGTATATTCAGCACCCACATAAAAgttggggaggtagagacaagaggatctctagggtttggtggccagacaggtacataatgagtgagttccaggctaatgagagaccttgtctcaaaaacaaaattgataacttctACCTGAACATATATGCATGTGGCAGCGCACACACACAAATGAGTTAATATTCTTGATTGTTATCATTATTTGTGTACCCAAACTGGTACCCAATTTCTTCCAAGGTCTCCACCCAAGCTTACTCATACCCTCTTTCTCTCTTCTTCTTTTTTTTttttaggtttttgagacagggtttctctgtgtagtcctggaactcactctgttgaccaggctggcctcgaactcagaaattctcttgcctctgcctcccaagtgctgggattaaaggcatgtgccaccacacccggctCTCTCTCTCCCTCTTCTAAGAGCAACTCTCTTCCCTGTAAACTGGAGTGAGCTTAGGGTTGAGGAAGCCCATAGGAGAGCCCTCCCAGAAGGTGGCCATACCTGGTCCAGGCTGTTCTGCTGGCAAGGGAAGGAGAAGGTGAAACCCAAAGGTAGGGACACGCCCTTCATGCCCATGTACTCAAGGAAGTCCGCGATGCACTGGACAATGTGGTCGAAAAGCTGTGAAAACAAAGGGCATTGAGAGAGGTGCCCTCGACAGAGGGGGATGCCCCAAGCATTGAGAAATGCAAGCTCAGGCAGCACCAACCCAGAGCAACTCTGCCAGGGCCCTGAATCACTGCCCTCCTCAAGCCAGGGGTGAGCCGAGAAAGGCTGGGGTCTTTGAAATTCGGGAGGTCGCTGAATCATCCCAGAAGCACAACCCATCATGTCCTCCCCTCCTCCCTAATGTATCACCTCTGCCACCACCACCCCTTTTTCTCCGGCCCCTCGCCTCACCTCTTCTCCAGTGCCATGCATGACTTCCTGTGGGATGGAGTAGATCTTGTTATGCATCTCTACGCCCCTTCGCTTGCCATTACGCACGCGCACCAGCAGGACCCGGAAGTTTGTTCCTCCAAGATCCAAGGCCAGGAAGTCTCCTTTCTCTGGAAAATGAACCCCGAATAATGAGTTGGGGTGACAGAATATAGCTCCCTTCGTGGGTTGTGGAAGACACATTGTGAAACTCAAGTCTTGGGAGAAGATGGAGTGTGAGCGCCACCTGGCAGTTAAGAAAGCAACATCTTCATCACCAGATCCGAATGGGCCCATCTGGTGGAACGCCACAAGTCGGCCCTTCAGAACATGAGGAATGCCAATACAGTATACTTAAAACTCTCTAAATGTCACATCTGAGCCAAGACAAATGCCACCTAGGGTACAATTCTGGAAGAGAAGCAGAAGTAGAGGGACTCTTCAAAGAAAACTGAAGTCAGGGAAAGTGGAatgcctgcagtcccagaacctgggatgtggaacttgggaagaggatcaggagttcaaggccagctttagccacactgtgagtttaaacccaacctgggctatatataagaccctgAGGCAACCGAACACGTTCCTTTTCCCTCTTAAAGGTGATCTTTGAGGGGTGCTTGAATGGAGCTAGGCCCCATATTAACAGGCTACCTAACCCTAGCTTCTGCCTCGAGAGCCGGTGCCAACCATGAACCATCACGGACCAGAAATTTTTTATATGGAGCCAAACAGGAAATACATGTAGACCCAACtaatgtagtatttgagagctgcctctggaatgcagattcctaggtctgaatgtcagctgacactggttgtatgaacttgagcgaggctctcttggtttcagttctcaGCCAAAGAGCAAATTTTTAATAGACAAAAAAATTGGTTGGTCTGAATGTCTgcacacacgaacaatcctagcataggttgattcaaggtggtcctgtacagcatcctggactacatttgaggccagcctgagctacattagacccatttcataaagcaaaaCTAATggggctgaagagaaactttagcagtttagagctcacactgttcttgcagaggttccaagttaagctcctaataagcacatcaggggtttcccaactccccctaacttcagctccagggggactggatatcttttggctcacgacacacagcacacacacacacacacacacacacacacacacacacacacacacacacggggcgtcgatgccacgtacctgtaccgtctggagtggcacacacataagtgggcaacatcttcacaGGGGCGGCCTCATGCGTCTCCTTGCTCAGACCCTGCTCCATTTCCACCTTCATCCTTCTCTTAACCTCCAGCAGCTGTTCATGGCTCAGCTTCAGAGACTCCAGGGTCTTCTGGCGGGCCCGGTGTTGGTCAGCCAGACGGTAAGCCACCGCCGTCACCATAGCCGCCCCCTTGCCGCTGCCATCCTCAGAGCGGAGGAAGCGGACATCACAATCGGGCACCAGCCTCCTCACTGCCTTATGGAGACGCTTGGCAAAACTGTGACACACAGAGAAAGAGACGGGTGTCCATGGTTACAGTCTGTGGTACAAATGGCCGCAGAGCTGACCATGGCCCCTGCAGAAGCAAGTGGTGTCTGTATCTCCTCTCTCCATCCCTCCCCCACCCCCATCTTAGGGTGACTAATATAAAGGCCCAGCTATTCAATCATGGCTGCCCTAGCTGGCTTCCCCCGTGAACGCAGTCACACAGACCCACTCTTTCTCATGGAACAAGCCTCAGCTCCTCTGCTTAGCAGATGGAGCCAGCCCCTGCCAGGGCAGACGACGGGAATCAAATGTTTCTGATGCCCCTGGAGATCCAGGCTCACTTCATCTGATGGCCCCCAGGAAAACACTCAACAATGACAACAAAAGCCTACGAGTATCTTTCTCTGGACTCTGGCCTTTCTCTTTCCAGTTCCTGCCAATGGCCTCAGAAGCATCTGAACAACTGGAGACAGTGAGAATGTTTAATTAGGGAGAAGCTCTTGGGTGGCGGCCCCCTGCAGCTTCCACGGAGCGCAGACTGCTGCGGACAGCACAGCCCAGCCCAGCAATTCCAAGGCTCCTCTCCCAGACCACCCACACCCACACAGATAGCAAGCAGCCTCGCGGCAGGCCAGTCACTGTAAAAGTCAGCCTCACCCAGTCACTGAGCCAGAACCGGCCTCTTAACCACATTCCCAGGTTATCCATGTTCCTACAGCCCTGGGCTAGAATGCTGAAGATGGATCTCAGAGCCAGATGGCAGCCACCAGCTCAGTTAAAGGTGCCCTGACCTGGCACTCACACTGAGAAAATGACAGAAAAATACGGGAACACAAAAGACCTCTTCTGGATCTCAGGTGAGATATGAACAGTTTGGAATAGACGAACAAAGGGACATGACAGGGCGGGAACAATCCGACACTAAAATCAGGAGAGGCTGGCGTCTTCAAGGTTGGATGGCCACTAACAGAGGGCATGGTACCCACATGAACCAGTCAGAGTAATGGAAGAACAAGGAATGAATCAGTAACTCATAAAAAGAGGGAGGTggcccgggatgatgttgggttgtgaggacgtgcttgcctgccatgcacagagcattcaatggcaaactctgcagaaaggtggtacacacctgtgatcctagaacttgggaggcggggacagacaagaggatcagaatttaagttcgccatgggcacagattgagtctaaggccagcctgggagggactagaggacaagcctgtctcaaaacagaGCGGAAGAGGAGGCAGCAAGCCAAGGACTCAGCAACCAGGAGCTCTAAACAGAGGGCAAGCCATCAGCTGCCTTCAGAAAGCAATCCCACTCATACCAAGTGACTGGAGAACACTTATGCTGTCTGGAAAGCAATACAGAGCTGCCTCATGGAGTCTCCAGGGGACCCTTCTGTTCCCAGCCCCGGCTTGCTCACTCACTGGGGATGTTTCTTGTAGACAGAGCCATCCACGCCGATGGTGGAGCGGAGTCGCTCCTCGCCCTTGTTCTCTTTGATCCGCCACAGCACCGCGGCCAGGGTGGCTGCACACAGGCTGGCTGAGCGCGTGGACACAATCTGGCAGATTCGGTGCGTGGCCACACAGTCCTCCTGCAGCGGGCTCAGACCCAGGCGCACCAGGATCTGGTAGGCCTTCTGAATTCCGTCCTTATCGCTGGGAAACAGATAGGGTGTGAGCAGTGTTGAATTCACTGTGGCCAGCAGTTCAAGGCTCCTCTCCCAGACCACCCACACCCGAGTATacacacacacacacacacacacacacaGAGGGAGGTCTGCTTTCTGTCGGCACTCTTTTATTTTCCACAAGGAAAACCTCGAAGTTCCTTTGTGTGTATCTTATAAAGACAAGACCCTGGTTTCGCCGCTGCAAATGGGGAGCAAGCGAGCATGTGGGTTCCTGCTCTCTGTTCCCCTCAAAGTATATGAATGGCAACCTTTGAGTACTTGAAGCCAGTGTGGACCCTACCTGTTGGCTCTCTGCAACGGCAAAAGCCAGTCTATGTCTGAAGACACGGGAAAAGGCTGGAGGTAGGGCCTGAGCCTCTCCTTGAGGAGCCCCATGCCTCTATATCACGCGCCTTCATCTCCCTTTGGGCTTAACAAAGGGCAGGCAGAACTGCTCCGAGGGTCCTCGTTAGCAAAGTTCTTTGAACTATTTCATTGCCCTTCTTTGCCACTAAAACTACTTCCTCGCTCCATCACGTCCCTGAAACTATAAGACCCCCTTCAATCAAGTAGAAATCTCAAGCGAGAACAGCAGATCTAAATCTTTGGAACATTGTTTAGTGAAAGCCTTTGCTCCCATTCCCGAGCTCACTGGGAAAAGCAAACCCTTGGCAAACACAAGAGCCCGAGTTCAATCCCCACAATTTATGTCTAAAAgaggaggtggaggccggtgggcccttggaggctcagtagtcagttagcctagcttacttagtaaattccaggccagtgagaagtctgtcttaaaggtagacactttgcagaacacatacccaaggctTAacacacacacacacacacacacacacacacacacacacacacacacCTCCAAGCCATGCCTCTTATTTCCCTCCCAGTTCCTGCCCTACTCATGGAGACTTGGTAAGCAGAGAGGCAGTGGGCCCAGACACTTACTCTTCAATATCCGAGACATCTTTGGTCTCGAAGGAGCCAGTGGTAAGGAGCTCTGGGCTGAGTTTCCCTTGGAACAGCAGCTCGGCCTTGGCCATCTTCACCAGGATGAGTCTGACCAGCTCCCCCATGTACATCCCGCTGATCATCTTCTCAAACCTGCAGAGACAGACCAGTGACGTCCCACCATCAACAGATGTACACCCTCTCTCCTCCAGAGGAGGGCAAACACGCATAGCGAGACTCTGTCTCAAGAAAAAATGTATCAAAGCAACAACAACAAGGCTTACAGAGGTTCCCAATAGGAAACAAGGTGGCTATAAGTAGATCAAGGTACAGATCTTCCCACTCATCCACTGACACTGCCTTCCCCAATTGTGAATTCAAGAAAATGGGTGACAATAACCTGGCAGAGgctaggaggtggtcaggcacgtctttaatcagcatttgggaggcagaggcagttcaaggctagcctggtctttaaaAAAAAAAAAAAAAGCCAGTAAGTAACCTGGACAGAGATTGGGGTAAAGTTCCAGGGCAGGCTAAAAGAGGGCTCAAAAATCTTGTGGTGagccgggcggtggtggtgcatgcctttagtgccagcacttgggaggcagaggcaggtggatttctgagttcgaggccagcctggtctacagagtgagttccaggacagtcagggctacacagagaaaccctgtctcaaaaaacaaaaCAAAAAACAAAAAGAATCTAGTGGTGAACGGGTTGAAAGCCAAAGGAAGAAATCAGGGCCAGCACTTAAGGAAGTCCAGTAGTTTAATTTTCCACCACTAGGTGGCACTCTTCTTTAGCGATTGCTCTTTTTGCCTGGGCCCACGTGACAGAAGAAACCAAGCCCTTCGGAATATCTTCTGGGTAAAGAAAACCCCTTGATGTCTCATAAAGGATCAGAGTCTAACAGACAGGAAGCATAATGGGCTGTGTACTAACTAGGATGGGCCACACCACCCAGGAGGCCCAGCAGGGGTGTGGCACAGAACACCTAACAGAAACTGAAAACCGGGCCCTCAATCAGCAAGATGGGACTTGCTCATAGTAAAGGAGCACTGGCAACgtggttccagacaccagctatgccaacatttaggaggttgaggcaagaggatcaagtttcaggctagcctaggctacacggtgagaccctatctcaaaCATtagagaaatggttctacggttcagagtgaggaccactctggcagaacaaaatttgattcccagcactcaggactatgtgtaattctagtttcaggggccctcttctggcctccccaacatgcatacagcgcacttaaagacatgcaaaaaagtcactgatgctcataacgtgaatagataaaTGGGAGACAggggacgcatgcctttaatcccagcactaaggaggcaaaggcaggcagatctctgggaattgaggccagcctggtctacaaattgagttccagcacagccagggctacacagagaaaccctgtctcagaaggaaaaaaAAAAAAAGTCAAGGTCATCTCAGAAAGTGAGTCCAAAATCAGCTAGGGTCACAGGAGAAGCTGTCTCAAAAAGCAAAAACCCAAAGTTCCAACACAGCAACTATTTCTGATAAGTAGCAACCCGGCTTCAGGCTTCAGCACTGCAGAATTCCAACGTCTACATGACGCTGTCCTTCGTCAGTGGCTCTTTACAACAATGACTGCCCAGCAGGACTTCAGGGTCACCCCAGTGGATAGTTTGGAAGACTCCCTTCCAATCACTGTCAGTCATCTATGGCTAAGCCAGTGCGGTTGCTCATTTTATCTTTGGACTCTCCCAAGGACACGTGGCTCCTCACAGGGACTTGGGTTCATCCCACTGAGGGAAGAGGCACTGAGTGAATGGCTTCCCAGCTAATCTCTCAGCACCCACAGCAGACACAACCACAGGATCCTAAAGGAAGTGAAAGAGCAGAGACTCACATCTCAACACGGAGGGGAGAAGCCTGTGACTTAAGGGAAGGCTCCCCATCTCCTTTTCCAAATAGAGATCAGAACTAAGTTTTTCTAAAACACTCACGGCACCACGAGAACCACAGAGATAAATGGTTCATTGCTCAAAATGGTTTTGCAACTGCTGTTTAGCCCTTGCAGCAGCTGCCAATCCTGTTTCAAGATCTTCCACAGAGAAGTTTTGCAAATTCGAGAAAATAAGGTTGTGTAAGAGTCGAAAGGAgggccagtgagatggctcagcaggggctgctcttgaaaagaacccagatccaatcaatacccagcacacacatggtggttcacaaccatgtgtgactccagttccaggggagccaatgccctcttcaaaatctccagtcaccaagtacacatgaagaagaaaagaaatacaAAGCCTTAAAAAAAAGAGCCAAAAGTAACAAAAAATAAGACTGGTGCATATAAGATTCAGGGTACAAAATGCTAAGCTTTTGCCTCCAATTTTCCCTAGGTCATAACTGGCCCAGAGGCCTAGTGTGAATGAAGACACAAGGCCTGGGACTCTGAATAAGCGGTGATTTAAAAAAAAAAAAAAAGTCAACACAGGGTAgcacatctttaatcccagcacttaggaagcagaggcaggtgggtgtctgtgagtttgaatccagctttcgtctacctagtcagctccaggccaccagaaactaaacagtgagaccccgtctcaaacaaaacaaaaAGCCAAATCAAGGCCAGGCTTGTAACTCCCTTGGTAGTGTTTGCTCAGGGTTCAATTCCCATCATCAAATAATACCAGCTACTTGCTGCAATCTTGGCATCCAGGCAGTGATGGGCCAAAAGCCACAAAACAGTGCCTTTAAGAGCCTCATCTTgccgggcagtggtggcacatacctttaatcccagtacttgggaggcagaggcagcagaactctgagtttgagactagtttggtctacagagtgagttccagaacatccagggctacccagagGGCTGCTGACCAAGACTCTGTATTGAGAAGAACCCAAGCAAAAGCATGACCACCCGAAGGGCTTCTCTCTGCTTTCAGCCCCTTTGCAGTCTTATGATCATGACCATTCCCACAACTCGTGTGCCTCCACAACCCTTTGGGGATAATGCAAAGGAAGCGTGGAGACAAGCCCTTGCTGTGTGTGGCAGTCCCCCCTCAGCTCCACACCCTATCCACTCACCCACCCACTCACCCCAAAGGAAGATGCTACTTACAGCTGCTTCCCAGGGTTCAGTGAGCCCATGTCGATCTCTCTGTCAAACTCAGTTCGGATGTCATTGAGTGTACCGTCGTCCCCAAAGGCCCCCCACTCCATGTTGATGCACATCCGTCCCTCGTCGCCCTCCACCATGTCAATGTGACGCATCTCCTCCATGTAGCAGGCATTACTGCCAGTACCTGATCAAAGGAAGAAACTGTTCAGTGCCAAGTGGGCTATCTATCGTCTTAGCAGCCTCCCTCTTCACTGCTTGTAGAAGTCTGCCTAGGGGCTGAGATTCCTCACGGCAGCCTCCTGGGACAACACAACATGATGGCCAGATCTCTTGTACACTGCTTAACCCTGTAAGTCCCGGCATGGCCTAAAGTAGCTGCATCGCTATCTTCGCACATCAGGAGAAAACACCCAAGCTAATGATTAGTTCAAACAACTATGGCCCTCACACCAGGGTCTCACTCACCCACAATGAGACCAATCTCGCAGTTCTGATCATCATAACCACAAGTCATCATGGTCCCAACCGTGTCATTCACCACAGCCACAATGTCAATGTCAAAGTCCTGCAGGGGAATCAAAGGGCCTTCATCATCACCAGGGGAAGACGGGTATGAAGACAAGTGTGAAGATGGAGAAAGAGCAAGACACACCATGCAAGTATGCTACTAACCCAATCCATGGCCACGCATGGATAGCAGTGGGGATCACCCAGGTGCCCCATCCACCTATTCTCCATTTGTCTTCACCAATAAGGACCATTTTCCAGACAGCAGAATGGATTGAATTCATCTTATTGCTTTCTCCCCCTCACACACAACTGACAGACCTAGCCTACCATCCCCTGCTTCATTCCCCACTCATGTCAATCATGCCGACATCCCTACTTGACAAGGAGAATCCTGAGGCTAAGGACCAAAGTGTGTTTCTTGCACCAAAGGCCAAAGGCCAAAGTTACTGTCCATCAAGCAAATTTCAAATCCCCAAAGGTATCTGATCACATACAAAAGTCACAGGCCAACATGGCAGGGATACTGAATACATATCCTTAAGCTCTACAAAACCATGTCCTATAGAGTCCAGAGACAGACAGGGAAGGATCTCAGATAGCCTGGGGACCCTGAGCTCCCACCCACTACTCACCCCTCTGCGCTGGATGGCCTTCCGGATCAGATCCACCACATCTCTGCCTTCCACGCCACTGGACTTGAACCCCTTAGTCCATGAGACCAAGAAACTCTAGAAGAGGGCCAAAAAATAATGATTTCCCACAGCTGCGACAGCAACATTCTAACAAGTCCTCAGCAGAGCCCTCTACCTCAGGCCTAAAGTCATGATTAAATTTATACATTGTCTCCTAACTAGCCCAGGAAGGAGGAAACTCAGCACACACACACACCCCAACCCCGCCCAAACTCCAGGACAGAAGAGTGCATATATGAGTTTTACTGTGATTCCCTGCCCAAGAAGTAGAAAGAAGCAGTAAATAgtgtgtgtgtgtgtatgtgtgtgtgtatgtatagtgtgtgtgtgtgtgtgtgtgtgtgtgtgtgtgtgtgtgtgtatgtatagtgtgtgtgtgtgtgtgtgtgtgtgtgtgtgtgtgtgtATccttgtatttctgctcctcctgcctctacctcctgaggcaggggtttcagtaggtactactgtggccagtttatgttgtgctggggacagccccctcggctctgtgtgcgctaggcaagcactctaccaaatgaacacagccccagcttcTTGGGTATCTTCCTTTCATCCGTGGGTCTTTCTCATAGCACTTTAGATCAAAGAAGATCTTAATTCTGCCAACATGGACAGGATACCACAAAGTATGTGGGGGAGGGGCAGAACACCACAGCCTCTCCTACTCCATCACCACCCTCCACACCTACACATAGCACATACATATAGCTTCCCAGAAGGACGTAAGTCTCAGTACTGAAAGCTCACATGCAAAATCCCCTCATCGCCACTGCCCCCACAGCTGCAGGTGGCTCAGGAGTCCCCTTCCGAGAAAGCATCTCGACCCGCCATACCTCATCCAGTTTCGTCTGGTGGCAGGGGAACGAGAAGGTGAAACCCAGCGGGAGCTTCTTCTCTTTGATCTGTAGCTTGTCCATGAAGTTGGCCAGGCATTCGGCAATGTGGTCAAACAGCTACGGGCACAAAGAGGAAGGTGAGCAGGGCGGCAGGAGAGTGGGAGACAGACTTAGGgtgaagggttctatcgaccgccaacttaacacagtctaaacagtctaacatgacctagaagacaaacctctaagcatgcctgtgtgggagtgggaagatctccccatgtatcggtgacagccttccaaaggctggagccacggatcgattaaaaaggagttaactgagccgagcaccacatctctctgcttccggactgtggacacagtcatgacctcagacccctgctgccatgtctgtcctcttccttctatgacagactataccctccaagtgtgagccaaaataaatgccccctttctagtgttacttcagttaCAGAAACAAGTGACTTATAAAGATGAGCTGGTAGAACTTATGTCATCTTTTGCTCTCCCAATGAAAAGGAAACGACATCGGAACTGACACTCAAAATGACATCTGGACTCAGTCATGCAATGTGTACAAAGGCATAAATAATGCTAATTAGTGCTCATTAGAGGGCATCTTCCACTTAAGACTTTTAAATTAATCTTGCTCAAAAGAACCCCCTAAATCATTGGGCGTCGTGGTTATCACAAGGTTCGAGTACATCAGAGAACAGAGCATCCTCTGCTGGCAGTCATGTTAGCCCAGCTCATCCTCCTAAACAGAAGCCCATCTCACTCCCAAGAAGTTCTTTGGCAGCCCTGACTCTTGATGGAGACTGACAGGATTCCTTCCCTTGGCGCTCTATGAACCAACTCAAGCAAAATAAACAACCTTTTTATTTTTCCTCTGTAAAAGAAAAAAAATTCCCTGAAGGCAATTCAAGGAAATTATTTTTTGGTTTGTTGTTTATCTTATTCTATTTTTTTTTAAATAtgtgtgtgtgtgtgtgtgtgtgtgtgtgtgtgtgtgcgtgtgCACGCGCGCACAGGTGTATACATGCAGTCACACTTGCGGCCATGGAgaactgcagttactagttctccgttgtgttgtgagttgcttgatctgatccctggtatacaaactcttgtcccctaataaaacagcaattgcttttaactagcaagtcatctccagtctcTATTTTTGATGTCCAGTTTCAAAACAAAAGACAGGGCAATGGCTGCAGGACCCACATAGCCACCTCAACATGCAGAGAGGTGCACTCCTGATAACTCAGGCTTCCAGTCAAGCCAGAGCCCACCAACCGAGAGGTCCCCTGTCACCTACTCCATACCCTCAAAGAGAAGCTTAGCTCTCTAGATGTTGAGAGACAGAGGGCAGGGCACGAGATATAGCGAGTAAGCAGAGGCTGGAGAGGGTAAACAGAGTCCACGGAAAGAGGGCCAAGTGGCAAGATGGAAAAGCGTGGAAGAAGGAGTGGAAGAATGGAAGACAAACAAGTGTGTTCCTTCCCAGAAAGGAGCAAAgggccagcgacacggctcagagggttggcatttggtgccaagcatacaacttgaatttaattcctagAGAGAAAGCCGACTGCTCCATGCTCTCCTGAAGCCTTCACACACCCCTCAAGAGACCCAGCAAGCTCTCGAAAATCTCTCCAGATCTCAACAATAAGGGATGGGGCTAACAGGCCCACCGGCTCCCTGATGTCTGGGCGATGAGCAGCTGGTGGAGTCAGGAAGGAAGGCAGCCTAATCTAGGAGGAGGCAGTGCTGGCTGAGCCCCCATCCTGGGTCCTGCCCATCTGTCTCATCCGAAGCACGGTTACCCACAGTTGATGCTGAACTTTGAACCTCCAGTCCAAGGCAAGAGCGGTGTTTGTTAGTTTTTCTTTATTCTTTCCCttttttcccagcgctgaagactgaacccaaggccttacacatggtatgtgagctctttgtcacCTTACAGCCTTTAGGGTGAATATCTTTATCCAAAGGAAACAAGTCACAGAAACATCAAGCAGTCACGGGAATTGGGGGGACCTTAATCTAAGGCAAGGAACAAAATATATCTTCCTAGTCCCCAGCCCTTACCTCTGTTCCTTCCCCAGCCGCCTTCCCCACAGAAGGCAAGAAAAAAAAAGCccctacattcaggaggctgagacaggaaaatggggaatttggcagcctaggctacctcttgagagcctgatatcaagtcaaactaaaTTCAGCTGGCTGGTCACATTTTCAGTTTGCCAGGCTTCATGTGACTCGTGGTTTCCCACAGGGCAAGTCCACTGAGAAAAGGTCTGAGTAAGCGGCAAGATCAAAAGCCTACACGCTTCCTAGAAGGAAGAGAGGAGACAGCTGCTCCCCTGAGTTGGAGGACCAACCCTCTCTTCCTGCTATCTGAGGACGGGGCATGAGATGCTAGAGAACTCAACACCTTCCTGCCATAAAAAGGCTCAAAGGGCATCGGTCTGCCAGCTCTGTGCTCCACACATTTCCTTTTCCTTATTGACAGGAGGATGTCTCACTCCTCTTGTCCTGAGATAGACCTAAAAAACCTCAGCAGGGTGTGCGGGCGCCCGCCGGGGCCAGCATCAAGAAGCTGAGGAAGAAGGGGCACCAAGTTCTCTGAGATAGAAACCAGTGAGCAGAAGATAGAGTTCTCTCCCGACAGGTTCACAGCTCTTAAGGACTGTTGCAAGTCTGAGGCTTGATAATTTAGCTTTTGATCTAATAAAACTGCATTTTAAAAATCTACTTCTTCCCCCTTTTAAAAAACTGGGCTggtaagatgacacttaacaacaagcctgaccaccggtgtgtgaaccccagaatctacatagtagagagaattgattcccacaaattatcctctgaccttctctctctctctctctctctctctctctctctctctctctctcAATGTAGCTAAAAGAAAAAGAATAGAAATGATTTTCTTTAATTTTGGTGGTTTGAGATGTTTTCACTATTAGGTATTTTATTATCAGGTGTCACATTTTAATTATTTTCATAGGTTTGACTTTCAtttggaaacaaggtcacactatatatagcccaggctggccttgaactctcaatcctccaggctcagtctctcaaatgctaggattacagatgtgtaccTGGCCCCGACAGTCTGAGATATTTCAAGGTAAATTTGAACTTTTGGGATCATGAGTGTTAGAAACAGATTCTCTTTCTAGGTGGACAGACACACTGCAAATGTCAACTCAAGTGGCAAGCCCTGGCCAGGCTCTGCAGGGAAAGCTGCCAGGACCATGGGATGCCTGTGACCTTCCTATTATAGAAGAAAGAGGTCTACGGAGACGAGATTCGCCCAACACATTGCACACACATCGCAATGTGGTTTTTCACAACCACAGCCAAACTTCTGGTGTGTTTTTGGCATTGTCTTACTTGGTTCTCAAATATTTGTCATCTGGTAGCAGGGCCCAGAACGAGACAGAAGCAATACGGAGAAAAGGCTAGCACATTCTTCATGAGGGGGAACAACACCGATGAGGTACAATACAGAGCAGGAAGACAATCTGAAGACTGGGCACGCTCCTGTGTAGAGCAGCAGGCCCCAGCAAGCCTGCCAGGTGTTGAAGTCACCACAGCTCCCCTCTCAGCCCATGACCTAACTAGCACCCTACCACCCTTCTCTACAAACAGACGACTAGGGTACTCCTGGCTGGTACCCTTTAGCTCAGAATCATACAGCGAGATCATCCCAAAACAAGATCCAGTCTTTTATGCAAACACCCCACCATGGTGCCTCGACAGTGTTCCTCCTGACATCACACAACGACCATGAGACATCTTCAGGAGAACTAAAATGCCCACAATCCCAGATACCCAGCCTCCAAAGGAGGCAAGCTGACCCTCAGGGCAAGCAAAGGATCTGATGATATCTCCCTTGCTGGCTCAGCTCACAATCAACACTCCTTTTTCCAGGACTAACCTGGTTGGTGGGAGAAGGTCAATTAGGCTGTTGAGGTCCCACCTCTTTCAAAATGTGCACGTAAACTTATTTTATTGCCAAGTGCCCTCTGGGCACAAAATATGGCAGGAATAGTCAAGGCTCCCACCACGGGAAACCTTACAGCTGGAACAGTGTCCAGGATGTCCTAGTGACTGTCCTGTGGTGGGAGAAGATGCAGGGTGTCCCACAGGCATTGGTAGCTCAGTGGCTACAACAGCAAAGCTGTACCTGGGTTCCACTGCCCCGCATGATGTCCTCGGGAATGGCGTAGATCTGGTTCTCCATCTCCACCCTCTGGAGACCATTGTCTGTCACCCTTACTCGGAGCACACGGAAGTTGGTTCCTCCAAGGTCCAGAGCCAGGAACTCGCCATGTTCTGTAGACAGACAAAGGAGGGCCACTGTCAATTTGGAAAAAGACAAGATCAAACTGACTCATGAGAAGCAGTTAAGACTTCAGAACCAGTCACTCGTGTCACTCGTGGGTGAGGGTGGGCAACTGGCTCACTTTTTCTCGATACACTTGGtccttccttccttccttccattctcttctttctttctttctttctttctttctttctttctttctttctttctttctttctttccttccttccttccttcctttctttctgtctgtctgtctgtctgtctgtctgtcAGACAGTCTCCATGTAACCCAGGCTTGcctccagagtcctaggattatcggcatgtcttatcacacccagctttatgTCTGGCTTCCTCGATGATAAGATATTTTTATTACTTTGTAATTCACATGAATCATGAGATAGTTTCATCATTCAGCTGTTCCTTTTGGCTtgtcaatgactaaaactagagttatctgggaagaaaaaactccacagagaaaacaccaccaatcagattagcctgtaggcTTTTATCCTACCAGGAGGCTAGGCATGGGACGGGAAGCACCTACGGGAAGCACCCAGATGCGGGAAGCACCCAGATGCTCGTCCTACCACAGCTGGCTGTGGGAAGTCACAGAAAGAAAGTAAGTGCTGAAGAGCCCTGCTGCCCACAGGAAGGCCAGGATGGCAGGCTTCAGGAGCCAGAAGAGATAGCACTCACCCCATTGTGGCCTCTCCCAGCCCCCTTCTACCCTCTTCACCTTCTCTTAAGAGGAGAGGGTTCAggctgaagagatggctcagtggttaagagcactgactgctccttccagaggtcttgagttcaattcccagcaaccacataatggctcacaaccatctgtgatgtgattttggcgccctcttctggcatacaggcatatatgcaggctgaacactatacataataaatcttttttaaaaagttaaaaaaaaaAAAAGAGGATAGGGTTCATTTCTAGGTAGAGGAGAAGGGACTCACTAGTGTGCTGGCCAACACTGAGCCTCTTGTCTGGTTGGGAGGATCAAAGGTATGTTCTGACTAAAGGATTTCATCCGTGCCCAGGTCTCTTGGACTCTCCTCCCCCCACCCTACTCCCAGCACCTTGCCCCACTCCCACCCTCAAAGGCCAGTTCTTATGAGCTCCCTGAACAGTGTCCATGGTCTCTTCAATGCAAAGCAGAAACTTTGGGGTATGCATCAACCAATTGAGGTTCCTCAAGACTCACCCGTACACATTTAGTGAGGTGCAGTGCTGGTCTAAATAACGTTCATCTGAAACCCATGTTAAGTATGTGAGAATCCTTGCCTAGAGCCTCTTCTACCTCAGGCACATAAAATTTGGTTTGACCTCTGTTCTCTTTAAACAGGAGCATCATACTTCCCAGGATTGTCATGAGAGAATTGAGTAATGTTCTTGAATCACCCAGAATTAGAGCAAGAACTTAAAAGACACCGTAAGCACCAGGCCCGTGGCAAACAGTCTCACACCTATTCAGGCCAGCCTCCCAAATAGCAAGGGAAACAATAGATACCTTTCCAGGAAAGGCCTCTGCAATCAGGCCAGGTGACTTAGCATTCCTCCTGCAGATACAAATTTTCCACTCCCTACTGGGCTTCTGAAGGAGTCACCACTACTCACTCCAAATGCCTGTTCACTAAAGGCTTGCTACAGCAACACCAGCCTCGACTCAGGGACACTGTCCACCCCCTCTCCATGGCCTCAGATGACTTTTCTAGACACTCGAGGTCCAAACCTCCACGGGCCATGGGGCCTCAGCCAGATTCTAGGAAATGCTATGTGGTGGTTGTCAGGGGGATGAAAGAGAAAATCAAGGGGAAGCTGCCTCATCAGCACTCTGAGGCCCGGGACACACACGATCCGCTGAATCAGTGACCATGAGGAACGGAGGTGGCCGGCACTGCCTCTGGCTGGTTATCATCCAAGACCAGCCCAGCTGGACTCGGCCCAGCATGGTCGGTCCATCAGGAATGTGGTTCGGCTGTACTTTCTGGTGGACCAAAGGGGACTGTAAATAATGAGTGCTAGCAGGAACTGGGGGTGCGCTCCCCATTCCTTCAGAATGCACCTAACTCCTCAGCGCATATTCCACACATCCTCACGGCTCCAAGGACTGACCCTGAGACGGTGACTCCAGAAATCCTAAGATGAGCCCACAGGGCAAGGGAGGGATCCTTCTAGGCCCAATCTCTATGGCATAAGACCACTCCCCACAGAGCAGGGGTTAGGTGACAGACTTTACACATGGTGACCAGGACTCACGGATTCTGGCTCAGCAGTGGCTGGGCACTTCACCTGGCTCCCTAGCCGTGCTGCCTCACCTGCAGAGCGCACAGCTCATCCTGCTGACCTGCCACGGCACTTGCGAGGTTTCGATGGAATACAGACTAAGTTACACATGCATCGTCATCAGTAAAGTGCCACCGTCCTTGTCATCCTTCATTGTCACCGTTCCTTGGCCCGAAAGGTCTTCTAACTCTTGTTGGCCACTCTGAATCTACATGCTATCACTAGTCATAGCTCAAGCCCTACCTCCACTGGGCTTCTTCCAAGACATTCCAGAATCCCAGAATAGCTTTCATGGGAGCATGTGACAGACCATGACTGCGAAGGGCAAAAGCATCTGTGGGTAGGGCAGGAGAAAGGTTCTGATGCAAGAAAATACACCAAGAGCAGGCGTCTTAGCCCAGCTCCCAGGTGCTCCTGCAATCCCACTGGTTAGGAGGCTGAGACTGGAGTCAATCTCACCACGTAGTAAGTTTCAGTCTAATAGGGGAGAAGGAAGTTTCTTAAGAAATCATACAAGATGGGTTGAAGAGAGACCCTACTTCTGGGTTAAAAcagaaaatgaggtgggttcccagcacccaccccaccttagaactccagttctaagggagcagacaccgtcttctaggacccatgagcactatatttacatggtgcacatgcatacaagcaggcatacatacaaagaaataaaagtaCATTTTAagctgggcttagtggcacgtgccttaaatcggaagaggcaggcagattctctgtgagtttcaggacagcctggtctaaatggtgaattcagagactgtcagttacagagagaccctacttcaaCATTTAAaataataataataataataataataataataataacaataTAAATTTTAAAATAATACACAATAAAATACACTTTAAAAAAGATATATCAAACACTACAGGTAAAGAACCCAGTAGGTTCACAGAGACTGCTTCTCTAAGTAACTACAGTTGGAATGTGATGCAACTTACCACCTCGTGTTGAATCTGTTCTCTAATAGATCACTCAGTAAATAAGACGTCTGACTGGGTTTTATATGATCACGTCTCATGACTTTTTCTTACTGCTAACCGTGCGGTTATCACTGGATCCTAACTTTCGATCTTTAAAAAATAAAATATGAgagctgatgagttagctaactgggcaaaggtgactgacaccccgtctgacaacccgagtttgattcctggaacccacatgatagaaggaaagacactcctgcaagttttccccggatttccatatttgctctgacacacacacacacaccaaacaaacaATAAATGTAATAAAAACTTTAACATATGATGGGAGTGGTGTCAAGCGCCTTCGATCACAGGAAGACTCAGATGCAGGGCTGGCTAGCCAAAGGAGTTTTCTGTTGAGCAATGAGGACTGGAAAGCTGTAAGCCCTGAGAGGGGAAATTCTTCTCTTACAAGGCCAAGAGTGAAAAACCCGGAGGCGACAGCACCCCCGAGCTGGGCTGGGTGTCTGGAAGTGATCCCTCTgaagttgagtcatttggttagggtcacaaaggggtgagagcgctagggtttgacgccaaggctcctgactctaaagcccgAGGAACCACAGTTTCCTCTCCGTGGCTCCCATTCCTGGAAACCACGGAGCAATGAGAAAGAAAAGCAAAGACAACCTCATGTGTGCCGGGATCCCCAGCCCACGGCTTGGAAACTACATCTACCATATTGAGTGGAGGGATGGAGGCACGAGAGCGTGAAGAGTGGAAGCCCTCCGCACAGTAGAGTCTGCTGATGAAGCTTGCTGATGCGGGCTGTGTGCATAGGCCCAGAATAAACTGGCACTCTCCTGTAACTATATATATCAAGCCCCTGTACCCACCTTACAGGCCTCTCTAAGAAAGACACTCTTCATTTAAAAACACCCACACACATAAAATAATACATACAAAATAAATCAGAAAATGCTTCTGTCCACAGCTCAGATACTGGGGACAGAGGGACAGGGTGGTGGCACCCTTCAACCAATCTCCCGACAGGGAAGGGCGAAGCTGCTCCCTCCAGCGGTGTATTTGCAGACTCACTTCTCATTCCACACGACCAGCAGCACACTTTCAGTTTGCCTCTTATCAGATACATAAGGTCAGTGAGATAAAGCATTCCAACTTCTCCTGGGAACTCACCTGTCACAGGGAAAGACTGGGCACTGAGTGGCAGTGAGCCATCTAAACATGGTAGTCAGTGTCACCTGAGGTAACACGACACGGGGAATTCATCACTCCTGGGTTGTTTAAAAGCTTCTATGAGGTGATCTGGTAACTGGCGACAGCAGCTGGGTTGGTTTGATTTTTGGCtgtgtgtgtgtgtgtgtgtgtgtgtgtgtgtgtgtgtgtgtgtgtgtgtgCCCCCGCCCGCACGCATACATGCACACATACTTCGTTCTGTCTGGTTTTTAAGGTTGAGGGACAGGGGCCTGTTGAATGTCCTTGCAGGTTCTATTTTCAACCATTAGGTACTTGCCACTAATTAAAATAGATCACCACCTGCTCCTCCCATCAGGAGCTCAGCGCAGTAGGCCGGGCCAGCCCTCTTACAAATATCTTGCAATAAAAAGGTGATTGGCCTTGTATTTCGAAGCTACCTTGCCCATTCTTCTGAGGAAAGTATGTTGAGAAATGGAAGTGTCCTCCTGGGTTGGGGTTCGGGCTGGAGCATGGCTCTAAGCCCCCAACAGTAGATAATTATACAAGTGATTTACAAATGATGGGTTCTGGGAACCCCTTCTAAATAAGGGGCATCCCACAGAGCTCCCATGGTTCTAAGCAAGGTCTGTGTGGGGCAAGGGCTAGGGGAAGGCCAAATAACAAACAGCTCACCTCCTGTGCCCTTGGCTCTAGCTTTACTTCTTCTCATTCTGCGCCCTAAGATTTCATTCAGTTACAGTTAACACTGAGGTGCACCCATCAAAAACTGAGGTGGGGGTCACCCCAAGAGATGCTTGTTTTACATTTACTTATactgtgtggatatggtcttcagaggacagctgtgggagtgtctgtcttttccttccatggtgctgctctcaggaagcgaactcagtttgatgggctgggctgcccgtacggactgagccacctGCCTTGgttttagatttttgagatgaggcctcctcactctctaggccaagttggcctcaaactcacggcaatcctcctgcttcagcacctccccccaagcacctgagtacagAAATTACTCAAGAGTAATGGGTATGGTGTGAGCCTCACTACCTTAACAGTCAAAAAAGCTGGGGATCATCCAGGCCCCTAGCTTTGCCCTCTTACAATGCCTCTCAGCCTGTTTAGACTCTGCTCCGCTCGCTTTGTCAATGCTCTGGGGCAACCAACAAGCCTTTTGTTATATCACTAAGAGGAAGGCAACACCTTTGTGTTTTCTGCTTTGCATATACTATTTTATGTTAGTAAAAGACTTTACAGTCATTTGTATAAAATGTACGATTAAAACTTATTTTGAgggactggagagatggctcggtggttaagagcactgactgctcttctgaaggtcctgagttcatatcccagcaaccacatggtggctcacaaccatctgtaatgagctctgacgccctcttctggtgtgtctgaagacagctacagtgtacttacaaaacaaaACACCCCAggggattattagaaggtgaatggttaagggcatgcactgctttcttccaggggacctgagtttggttcccagcatccacactgcacagctcacagctacctttcactgcagttccaggaacagcgccctctagtggccttccagagcactgctctcatgtgctcaaactcacactctaaagcatatacataattattaaaaaataaaTAAATAGAAGGGAcaggagtagtagaatgtgctataatcctagcacttgagaggcagaggcagcctgatctacatagcacattccaggccagccagggctactcagtcttaaaaaataaaCACACAAAAATGAAATTTGGAAAGAACTAACTAAATCCATCATGAATAGGCGAAATCATGTCATCCCCTGCCATCGGTTGCTACAGCAGTGAGAATGGAAGACGACCCCTAAAAGCGCCACAGTTGTTCTGATTGCCACCCAGCCACACATTGACCCAAGAAGAAAGAGAATGGTACGAGTCATTTCTGCTCTACAAGAATACCTTTCAGGTACAGGCAACAGGAGTTAGCATGAATCTTCTGGATGGAGAGAGGGTCAGCATACATGGAAGTTCACAGCATAGTTAAGTGTCAGCGTGTCGGTCTAGCATAACTATATAACATGGTACCAATTTATAGCTGCCAAAATATGTCACGTCAAAGTCAGCTACAGCATAGAGCTGATGCAATGGGGGCTACAGGCACCTAGCGAGTGTCATTGTCAAAGTTGTCACTGGGAGGGAACTGTCTGGTTCCTAGGCAGATAAGATTGTGCGTGAGTAACCTGGAGGGGATGGATGTGTCTGGGGCCAAGGAGATGCTGCAGCCCAGCCAAACAGCAGCAGGGGCCTAGGTTCAAGGGCCAGACAAGAGCGTCACCCTCCTGCATTGGATTACTCAGTGTACAGGGATAATGGGACTTCCAGTGGTGACGCTGGGGAAATCTTAACCTTAACTAAACTAGCCAAGTGGATTGCATTGGGCTGACTCACAGGACTCCCAGACCTCCTGGCCACCGAATGCTGGAAGCAGCAAACGGCCACGTGCTTCCAGCTCACCCACTAATACACAAGTAGATTCCCACATTCAGTTTCTGAACTGTCGCTTTAACAAAATCTATGCCCCTAGAAAGGGGCCTGCCCATATCTGCCCTACAGAATTATTTATTGCCAGTAGCCTCTGGGGACTGCACGAGAGCTGTGTGAGACTCTTATCTTTAAAAAAAAAAAAATCAATACAACAACAATCAGGTGTTTGACCCAACATACGTAAATGCCACCCACTATAGAGGGTATCAGTACAATCTCTGCTTAGGTCGCCAGCACCACATCATAAACCCCCTTAATTAGCTCGCTTGTGTGCTGCAGTTCACTCAAGCCTCTACTGCGTAGATGTTGGTAAAATACACAGCAACCAAGATAAGGGGCAGCTTGCACACAACCAGCCTATCACAGAGCTGGGCTCCCTTTATCACACCTCATGCTTGCATTATATGGCCCAGGATCACGCTCTAAGTGTATCATCTGCGTAAGCGTTATCTCCCTAAGGACAATTCTCACTCTTCAAAGGCAGGCCATGGGAGCTTGGCACACCACGGCCTCACAGATGGCTGAACCTAGGAGAGGATTTTGTGGATCAAACCCAATCAGAGTTCCCAAAAGAAGAATGAAGGCATTGTGTTAGTCATGTGGGAGGTACAAGGAAAATAAAACATGCTGTTGGCGGTGTGCTTCGGGGCTGGGCATTGGTTCGGGCCAGCCCGAGTTGACTGTCTCACCGCCTGCTTGCTACGGGACTGTTCTCTGAAAATTGACTCTCCTTTTCTGTAGAGTATCTACCACTGAGACCCTGAGAGGGCTTTAGCACAGAACTGTCTGTTGTCTGGTACACACGGTCTAGTTTCTGTTTCAATGGCCGTACCTAGTGCAAGGATGCTTGGAGTTTCATTTGTATCCAGTGACTTGGGGGGGTTGTGTCTTTCTCACAACTCATCATGTTGTGAGATCTGTACTTCAGTATGCTTATGTATGTAAGGGGGCATGCAGGACACCTCAGGCAAAGGTACACTTCCAGTATGGCGGCACTGTAAACAGGTGTAGAGCTGAACTGTCATATACACCACTTGTCACCTGTCACACAACCTATACCACCCAGATCTGCATGCACATGCTCACACTCGCACACCTGGTAACTTTGGCATCTTCATgtttgactcctggcctcagcctatcatcagttgtgtgaacttgggcatattatttgaccatcccagcttcatattttaaaaaaggaggataaaaatctctTTAggggctggtgagatgagtctgaggaaaatgtacttgctgctaaacctgacaagctgagttccaccgcccaggactcccatggtaaaaggaggaagctattccagcaagttggcttccagactcattgaagtgcacacatacacacacacaAGGAAAGAAACGGAAACATATACCTCATCAAAGTTCTGTTTAAGTAAATGAATTggagctagagatgtggctcggcactaatgcgttcttactgctcttatttatagaggacccaggttcgattctcagtacccactgtgtgggtcacagtctgtaactccagttcaagtggacccaacgccctcttctgacctctcagagctcctagatacacacggcatacaggcaggcatgcaagcaaaaaaatactcacacataaaaacataaataaaTCTTTTAAGCTTACGAGGTAGGTAGGTGCTCAGGGCAAGCCCTGGCTCCCACAATGCACTTTTTCAACATTTGAGGTGCTTACTTAGTGAGATACAGGCTGCCCTTCAGAAGTTGATACATTTATCAGAATGTCACACTGAAACACAGATGTGGTATGGCTTCCAGTCAGAATGAGACAGAGCCAATCTAGAGGGCACTGCAGAGCTCACAGGGTCCCCTCTCCCCACTTCAGGGGACTCTTTGAAGGTCTGGGAACACACACATTCATGAACAAGGACACATGCTAAGTGCTGGGCCAAACAGGCCACAAGGCTTCAACAAATATTCTTTCTTTAGGAACTGTACAACGTCTTGTTAAACCAGATCCCAGAGACTCTCAAAAGTTGTTCCTCAAGGGCACGCAGGGATCCAAGGTCAGGTTCCAACACAGACCCGCCTTCCACAGCCCCTGGCCGGCTGCTCAGCCCATCCCTCAGGCTCTCAGTCTGCTTCTTGAATCTTCCACTGGCCTGAGCTCACTCTCTCCCTTTCTCCCCAGATAAAGACCTCACACTAGTGCCCCCCACTGCTATGGACCCCACAAATGCCAGTCTAGAACATGGTTGTCGCTGTGGCAAATGAATAGGCTCTACCAATAACAGGTTGCACCTCTACCTCCAGGGATGCTACCAAAGCCACTGTGCAGGTTCAACCTCCTGGCATTCGCTTTGAGAGTTCCCTGTCCATCAAAACccccaccccaccccaccccacTAGGCCATGACTATGTCTCCCATGTACCCAACCTGTGCTGATGAACTCACATGGGTGTGGAGATTCTGTTCATGCCCTCAGACACTTCCATCACCTCCACCATATTAGCGAAAGGTCCACGTGAAAGGCTTTCTGGGATTTAGTCCTCATTTAAGGGAGACCAGGAAGGCACAAATTCAAAAGCCTCTCCCCAAGGCTATGTAAACACTAGCAAATGCTGTGTGTGTGTGGGGGGGGGGGGGTACTCTGCAGCTCTCTGCAGTTTGGGCACCAGGCAGGCTGGATGTTTGTCTTCCCCAAAAAGTCAAATAACACTCTTTCCAATGTCAATCTTTTGCTTGGTGGCATCATAAGAGAGACTATCCAAATAGGAGATTAGACAAACCTCAACCAACAGCATCTTGCTTTAGTCTTAggggctggagagatggctcggcacggaagagtaataatggctcctcttccagaggacctggattcaattcccagcgcccacccacagggcagctcataaccatctgttccagggaatctggtgccttctggcctctttgggcagacacctatgtggtgtacagatatacacgcaggcaaaacactcacacacataagaataaaaCGAATATTTTAAAGAACTGTTACTGGAATGGACACAATGGTTCTTCACATCCTTCCCAGTTTAGTTTAACTTATAGGACTTTTCAACGGAGCCCATGAAACAATCAAAACACTATGAACGGCACTGACCAATATGCCTACAGAACGTGCCCATCAACCATGGTCGACATGGTTAACCACCACTGTGTGTTATCTATGTGGGACTGGGAAACCCGAACTGAGGCAAAAAGCTCTGCTCAGCACAAGAGCAGAAAACAAATGGCTGTCAGCGGTACACAGAGAAGGCTGGCTGACAAAGAAGAGTTTTGACTCTGGCTGCAGTCAGAAGCAGCTGTGGTAGCTTCCAAGCAGTCAGACGGCCATCCCCTCCCATCCCATAGCCTTCCATTCATCTCCCAGTGTTTGCATCAGCTTTCGTTGACTAAGGGTTAAACCTTGCAAGAGGCCTTGACTGAGATTAGTTTTATGTCTCAGTGGACTTTCAGTTCAGAGATTCTTGGGACAGTTAacacacacacacacacacacacacacacacaATACCTGTCCCATCCGGAGTTGACCTCACAAAGGTGGGCAGCATCTTCACAGCTGCTGTAGGGTGTGTGGTAGCTCCTAGCCCCTTCTCCATCTCCTTCCGGAACCGCCTAGAAATCTCCAGAAGGGTCTCATCTGAGAGACGCATGTGGTAGAGATACTGGTCAACCTGAAAGGGGGCAGGGGACAAGAATAGGTTCTATGTTATTAAATATAAcccagccttgcaaaaaactctagtcaggcttgtaaccctagcactcaggtggctgaggcaggaggattgcaagttctaaggcagcctgggcaacaccgtaagaccctgtctttTTTGTGTTCTCTTGAGCCAGCAGTTGAATGTCCCTGTGCTGGAGCACTTCCTACCACGTACAAGATTATCTGAACCCCAGCAGTTCAAGAAACAAGACAGCCCAACAAAACCTGCCAGGCCTGGCACATTCCTAAATTCTAGAACTTTGGAGGCAAAGGCAGGATCAATTCTAAGTCATCCTTCTGACAGGTAAGTCCAATGTAGGTTACATTGGGCTGATATacaaacaaacagacagacagacagacagacagacagacagacaTTTAAGTTAACTACTAGAGAATAGAGAACTGGAGGAAGACAGTGGGGAATAGGAGATTTTCAAAGTTAAATTAAAACGCCCATAAGCATCCTGTAACAGAGGCTCATGGCCTCCCTCCTCTCTTGTTGCCTAAACTGGCCTTCACCTGGCATGGCTAGTCCTGTCAGTGAGGTCACCCTCACCCCAGGGCCAGCAGCCATCACCTCCTCCCCACATTTTACACAATCCCCCGGTCTATTTTTAACTCTTCTATCAGTTTCCCAACCCCCACCCCAGACTCTTGGATTCCTGAGTTTAAAATCTGTTAGTTGTTTCCCACTCtggtagtttgaataagaatggtcctcttagggtcaaatattggaaagcttagtcaccagggagtggaagattaggaagtgcagccttgtaggagcagatgaggtattataggaggaattctgtcactggaggtggcctttgaggcttcaaaagtccacaccaggcccagtgcctcttctctcagccccgccctctctccctccctccctccctccctccctccttccctccctctctctctctctctctctctctctctctctctctctctctctctctctctctctcttctccctccctcctactccagtgccatgcctgtctgattcacatcatgatgatcatggactaaacctctgaaactgtaagccaagtccccaattaaatgctttcttttataagagttgccttggtcaaggactcttcatagcaacagaacagtgactaagacaCCATTAAACATCCTAAGCCTCACATAAGCTAACCTCTGCCCCCTCCTAACCATAGGGCACGTCTCCGGTCTTTGTGACATTCTTTCTCTGGTGTAAGTCCAAGCTGTCCTCCCTAGCATTGAGAATCCTGAATCTATAGGTACATCCCATCCTGCCAGCCCTGAAGACCCATTTCCAGAACCTGCCCTGGCCCTCCTGAAGATAACAAGTTAGGCCTCCCGGCTATTCCAAGGACTCCAGCTTTTTCTTGCCCCATACCTAGTCCACAATTTGGAAATAATGTTACTGGCCTCCCAACTTAGTTACACTCTGATAAACCTACCCAACTGGCATTTAACCCTAACTTCCTGATCACCATAACTAGGAAAAACTAGTCACATTTTGTGACTTGGGGCTGCCTAGGAGCTGACTTCCCAGAAGGGAGAGAATATCAGAATCCCTCCAACACAGACACAAACAGTTGGCCTGGCAGGTCAAAGGGGCAGTTGATCCTTTCCTGGACTTGCCACAAATGTTCAATTAGAGCCCTTACTTTATTAATTCCTAGGAGACGGGAGATCCAGAAATCTCCCAGACACCTCCTCTCCACCATTTGCCAAGGAGGAAGATGAAATGCCAAAGAGGTAGAGGAAAGCCTGCACGGCCCTGTGATGAGTCCCTCTGCCGCTTCCAAATTCTACAGAAGAGCTAATTCCCCAGCAATCTCTCTGCTTGCAGGGGAAGTAAGTGTTTTACCAACCAAGCTGTCCCCAGCGCCATCTTTGAGGTTTTAATTGCTAATTGTTCTCTCTTAACTCCAAGAAAGAAGTTAAAAGGCTGCTCTTTATTGGAGGCCCGTTACCCTTTGCTCAGCGGATCATACCTTTCAGTAGCAGATACAAATGATAAGGGCGATGTAGCCATTAACAGTAGGTAAACATTTTTAGCATAGCTTCACCTCAGGACCCACCTGACTTGCTGGCCATTGTCTTTGGTCTAGGGGTCCACAGACCTGGGTTAGAGATCCTAGGGCCACAACTTCCTAAGCATGCCACAGGTGCCCATTCATTGCTTGGCAGGTGCCCTGCACTGGATAAATGGCAACCAAGAGAAATGGAAATGGGAAACTAGGTGCAGCCTCTAGAAGTGAATGCAGATTCCAGAATCCTTCCACGTGTCCCCCAGCGCTCAGCCACGGGGAAGAAATGCATATCTGCCTGGAGTCATGGAAGGCCTGCTCCCTACCCACACACACACCTAGATTTTACTTTTACACACAGCAGTGAGACTCCAACCAGTCAAAACACATCCTTAGAAAGGGGTGCCCTAGTAGGTTGACCAGTCATTAGACTGTGTCTCTTTTACCCCAAGAGTTCCCTAACCTCACACTGAGTGCTTTCCAGTCTGCCCTGAGCCCGCTATGTCCCTCAGTCCCACTCTGGGTCAGTTTAGAGCCCTGTCTTCAATCACAGCTAATTCCGTCTCTGATTTTCTGCTTAGTCTCCATCCTGCCACTGGAGATAAGAgttcccaaacttggctgcaatccagaatcatgtgagatgctctcagaaGCTTTTTTTTTGGGGGGGGGGGGGCACCAGAGGCAGGGGAGGGAAGGGTTAATTTTTTTTTTTAAACTTATATTAGTTTGGTGGGGTTTTTGTTTTGATTTTGTTTGGGGGAGAGAAAACAAAATGGGTAAGGAGGTGGGGAGGATCTGGGAGtattattattattattattattattaAAGAGGTCATAAGTGCAggtggtgccagcctgtgatccctgcacttgggaggcataggcaggaggaacagacattccaggtcatccttggctacattgcaagtttgagaatggcccaggctCTAAACATTCGTTCCAGGCTGACCAAATGGACACCCTAGGGTTGAGGGTTCCAGTATCCTCTCCCTTCTCGTGGGCGGGTTCCTGCCGCATCAATAAGTGATCATGGCTGGACAGGTTCTTCTAGTCAGTGTTGCTTTAGGTCATTTTCTTCTATTTTGCCAAGTCTTTTtggctcaagcctgtaatcccagcagcacccaggaaactaaagcaggaaaacctgcagcaagctggaagctagccccagctgcagagtaagttctaggccagcctaggctagagagtaagaacttgccttttcaaaagaGGAGCAGGCAAGGGGGGGGTCAgagataactccaccagaaaaagtgctctccttgtaaagagaggacctgaattccacccccaagaacccatattttaaacagccaggggatgttgcacaATTCTGGCTGCAAGGCACACAGATCCTATAACATTGAGACGTGTGACAGCATTTTCACTATAAAATGGGGAGCAGAGACCCCCCGCAGCCAAGACAGAATGGAGGTGCCAAGCATAAAGCTCCCCAACTATCCCCTTTCCTCTTCAAGAAACCTCTGCCAAGCGGCACGCTGAGAGGGCCAAAGGCAATTCTCAGCACTGCTTAACCAAACTCAAGGTTTAAAATAGCAGGGCTTGTTCCCCCAGACTGTCCTCTTCTCCTTCTGTGTTGATAGGCTGGGGGGAGGGGGGAGGACAGAGGGAAGGGGGAGGTGCTTCCCTTTAGGTCTCTATCTAAATAGGAGGCGGAAAACAGAAGGCTTGCTTGCTGATGGGGGAGTCACAGGCAGGCCTGAGCAAAGCTGGCTGGGGTCAGGGAAGAAAAGGTTGCCAGGTTACCCAAGGCAAGGGCGTGTTAGAGATAGCACTTCTAACTGATAGCTGTGCAGCAAGGCTAAAGAGATTAGAACTTCCTCCTCGTTGGGCAATACAGGTCAGTTTCATTTCTCCCACGACAGGAGCCTGACATGACTCTTCCTCCAGAGTTAGAGGTGGAGCCAGGACGAACTCTAAAGAAAAGAAACCCCAACCAAAAAGGGAAGCTGGTATCATCCAACCTTTAAATTAGTCCACATCCCTCCAGAGCCACGCCAACGGAAGAGGAAGCCTGGAGcaggcagggcccttcacatctagaataccagcaccaaggaggctgagccaggagaattacaaattcaaggccagccttggctacctagcgggtctcctcaaaacaaaacagaaATCTTGGGAAGGAAGAAACACAAAGATCGCTGGAGCATTTTAAATTAGCCGCATGGTTAACAGGTCTTCCTTCGGCCACTCGATCCCTGGTGTCAGGGCATTTGGCTGCCAGGGTCACCATATTTTTTGGTATGTGAGATGAAAACCTGATTCCTGAAGGGCTCTGTCCATTTTGCCTTGATTGTCAAGAAGCTCAGAGTTCTGCACTCAACTCCTGCTGATACCTCCTTGGTCAAGAAATCCTTTGACCAAAGCTAACCTAAGTCACCAAATCCTCTGAGGGCACTCCTTCCTGGCAGAGACTTGAGGAGATCAAACAGGATAAGTGACTGCACCATCTCAGGACATCAGAGCAAAGCCAGGAAAGGAGCCAGAAACACAGCCGACGCCCCCCACCCCACTCCACTCTCCAGTCCTTGGCTATTTCCATTAACATCTAGAACATTTTCCTCCAGCGGTCCTAGAAGGACCACTTCAAGTTCAAGGCCTACCTGACTTCTTATCACTTAGGCTCCTCCTGAAGAAAAAACTACATCAGCTATCTCCAACCTCAACTCCAGCCTGGCCTCCTACAGGACAGTTAGGATAGGCCCTGCCTTTTGTGAGTGGCTCGGTCTTGCTTATTTGAGATCAACAACAAAGGGATATGAGGCAGTATCACACAGCATGAGAACGTGCCACCAGCCACCACCTACCTACACAGACACAAATGTGTATCACAAGATGATCAGTGAAAGAAGCAGGCTCAGGAGGGAACAACCCACCCTGGGAGTCCACTCGCCTGAATCCAAAAGCCAGGTCTGTGGTGTTGATACAAGTCATACCGGTCccaggttcagtggtgcctgcctggaatcctggcacttaggagttggaggaggaggagaatcagtgtttaaggtcatccttacttaggtaagcctgggtatatatagtgagaagcttgcctaagaccaaaGACAGTGATAGCCCTTTCAGGGTGGGGGAGGGGAAGGGGTGCCAGACAATGGGGTGATTCTGGTGATGATGAACAGATGGAGGTGTTATCCAGACCATATTTAGTTTCATATGTGTATCATACTTCAGTTTAGAAAGTTAGGAAGACTGAGACTGGTGGCACAGGCCTACAATGGCATCCAATCTCAGAGTCCTAGGTTCGATCCCCAGTACAGTACAAAAATAATTTAAAATACACCATgcttgtaagatagctttagtggataaaggagcttgctggcgtgccgaatggccagagtatgacccgaggaacccatatagtaagtaaggtgtcctgactcccaagtatgcaccacagcatgcatacacacacacacaAGAAAATGTAAAATACATACATGCTATGTACATTAATTTTACATATAATCAATACTGTTTTATAtggcacatatttgtaatcctagcactcaggaagctcagacaggaagagctcaagttcagggccggcctgggctacatagaaagaaagacagtataagacagtaggagaaacAGAAGGATTTGTCCCTAGCCTACCTGCTATGCACAGTCAAAGCACCCAGGAGAATGGAGGGGTCAAGAGGACAGTTGAGAGAGGGTGCCCTACTTTAAACCTAAacacacatacacacacacacacacacacacacacacCATTTGTGTTTGCATTATTCCTTTGGCAATGTTTTATCATCAGGCTTTGACGAGGAAACCACGGCTTGGAGACATTTCCCCAGACCACAAGCCTAGTCCCTGGCAAGGCCCACATCCTCAGGTGGTCAGTTGGTCCACTAGGCACCCCAACCTGGGGCAGGCACAGGTATGTCCACCCTGGAAAAGCTGGCAAATGGTATCCATGTCCTCATTAATTACAGTGCTCACAACTCCTCCAGCAGGACCAAGGCTGTGCACACAGGGAACAGCAAATGTATGCCATGCTGTGGTCGAGGGAAGACCAAGTGTCTAATCGAGGAGGAAGGGGTGAGCCAGGGAAGACGCTGAAAGCGTTAGCTGCATCCCCAGTCTTCTAAACGCAGCTGATGGAAAATAACAGATCTGCTGAACAAACGCTTTCCTCAAACGGGGAAGAGGAGGTTCTTTGCTGGGGCAGCCCTGCCATCTTGGGGAAGACTCCAGCTCTGACTGCTCATGAATGTGGAAGAGTAGGGAGCATGCAGGCCACTCCTCCAAGATCCTGTTGCCCCATTGTGAGTCACAGGCCTTCCTGTTCATCCAGTTCTTACTCTCAACCACGTGGAATGAGACAGACCAGGTGGCAGCACTTGCCAGGAGCCAAGAGATGGGCATTGGGAATACCCAGCCTGAGCCCGCCAGTCCCTGGATTAGATATTTCATCCTGGAGGGAGGGAGTGTTGCAACAAACAACAGGTCCCAGGTGTATCTGACACTGGCTTGGTCCATTTACCCATGAACTGACTGAATAGTAGACCAAGTGTGCCAGGGCTGGGCACAGAAGACACCAGGGTAAAGGAGACGGCAGACTCTGCCAACAAGAAGCTCAGTCCATAGCAAGAGGCCTTACAAACCCAAGGTGACACACACCAGTAGTTTCTCTTCCAAGGACCACTTTTTGTTTGTTTATTCActtgtttgcctgggttttgagatggtagcccaggttacccctcaaaccaaaggcaatccccatgcactggtcactcaagtgctagtatttcgagtgggtggcacATTTAGCTTCTTTAAAAAAAAAAAAAAGTGTATATTAAATCATCTTAAGAAATGTTGggccagaggtagagttcaaggtctcaaccctagaagaattacagcaactagagaacactgaagtgtgggagatagtatctaccctaggaaaagcacaccaaatggttatccaatcccacatggtcagccctgaaaacacacatacagataacaacactatacaggctgagcacgttgtatttgtacatttaggaaaatatatgtatatatctatatacacacataggcacatttacacatacatatatttgcatgcatatataaatatatacaagaaaattcatgtaTAAGAAAAAAGCCATGAATCTGAAAGAAAGGGGAGGTACAAAGGGACTGGAGGGAGAAAAGCAAAGGGGAAAATGAtgtgtgtgtgtgtgtgtgtgtgtgtgtgtgtgtgtgtgtgtgtgtatgATGCTCAGCTTCTATGAGTAGTTCCCGCAATAAAGGTGAATGCACAGACATCTCGGTTCCAAGCTGACCAGACATCTGTCCCTCATTCTCACTAGATCCAGAAGACCATGAGCACCAACTCAGCCTTTGCCATATTTGTGATGTAAGCAGCACTTAGTACTCATGAGCTGGTATTCAAGAAGGCTTGGCTACGGTTACCAGCGTGGGACTATGACATTAGGAAAATGAAGAGCTAGCTACTCCAGACTGACCCTGCTCTTCAGGTCACTACTCACTATTTCCTGACACCATTTCCTACGTGTTCATCTACCTACAATGCTTGGTTCTCTAGGTGACACAGAGGTGACTTTACCCTCTCTGGACAGCCCCAGGCCAGGGACCACAATCTGCCATGCAGCTTGTGACTGGGAAACTGAGTTCCAGATTCGGGGATTTGGGAGAGTTTAGACGTGTCTCCTAGTACGCAGCAGCTGGTGTCTTCTCCATAAAAGGGCTTAGATGATATCATCCCAAAGGTCCCTTTCCAACTAACACCTGACTCATAACCCAATAAAACAGCCAACCATCTCCAGTTGTCCCCGGCTGTGCCGGGAgttaagtgcttggttctggagtgaggctgcctgcctgagtttaagctctgccttggccttgggcaaggtactgacctggtgtcttcacctgaatgacataacagtatctcctcTGCAGTAAAGCTAGAGGGTGGCCTGAGATATTTCAGTGCACTGTGTCCGACATGCCAACTTCTACTAAGTACAAATGACGTTTATCTAGAAGATCTTCATTGGAGAGCAAGCACAggtggctcacacataaaatatcccaggtctccagagggaggctgaggcaaagggattgcgtgcctgaatcacatagtgggttccaggccagcctgggctacagtgtaagaccctgtcccaaCCCTCCTTCCCAATAAAGCTAATAATTAGCTTCCAATAACGAGCTTTATAGGGAAAAGGCACAGTGAGTCTCGATGGCTTTGTATAAAGCCCTGAACCAGGAAAAACTCGGGGAGTGATTAGGAGGCAGCCTCTCAGCTGGGAAAGGAAATTACTCTCAGGTGCTCTCACTGAACCCCCATTAGAGTAGCAGGTGTTTATCTGCCTTTATCCCTTCCTTTATCAGGCAATAAAGATGCTAAAAGGGAAAGTTGGATAGAAGGGCTTTTAAGAACATGGACTGCCCCCCAACCAAACCTCCACAGACAGACAGGGATGCTGGGCAAGAGACCATGAGGGAAAAGGTGGGTGGGTGGGTGGGTCCCTTACTCCAAGGACATCTGGGATAGGCTCCAAGATAAAGTTTCAAGAAAGGAATGTCCAGAGATCTGGAGACAGAAGCACTAAGCAATTATGGCCACCCATAAAATGAGATTCTCTGCTCCTCGGGCCAAAACTCTCGAATCCTAACCAAAGAAGACAAAACCTATTAAAGGCATACAAGGGCTAGGAAGGATGGGGTGGGTCACAcccagcactataatgtcagaaaactgattgtcatgagtttgggattattaacccgggctacatagtgagttccagctcagcctggactgcgtgcagtatgagaccatgACGACCACGCGTGTGCACATGTGTGCACGCACACAAGCAAGGgggactggagatgaagttaggtgatagagcacttgcctaggaccctgaaatttaacccttggcacctcaaaaaGTAATAATGAAATGTAAGGGGAGACTAACTACAACACTATGAGTTGGGGCTCCCCAAGGTTAGTAAGAAGAAAAAAGCACTCAATAGCCCACGGTCTGAATATGAGGGGCAAAATACCCTACTGTGGTTCTAGGAGACCCCACCCTACTGAACCAAAAGAATGAGGGCATGACAGCCACCTCTGGTCCCTAAGGGAAGGCCATGAATCCAGGGCAGCCACAGCTTTCATGGTCTGGTGAGCCCACCTCTAAGGTTTTCCCCAGTGGGAAGCCAGCCCCGGGGACAACGCCAGCTCCCAGAGGTGCACACTTCCCACCTGCCCTGGACATCCTCTGCCCTTTCACTCCTGGGAAAGGGAGTTACTGGAAGTCAGCTACTTCAAAGGATGCTTCAACCTGGGGTGCCCCAGCTGACAGTCAAGTCGAGACCTTTGAGTATGACTGTCACTTCCCCCAGAAAGAACACAAGTGAAAGAACGTTTCCTAATTGGCCCAAACTGCCAAGGCTTACTCTGGACTCCATCTTGCAACCTCTCCCAAGAACCACTGCCTCTGTCCCTGGAAAGGTAGCACCAGCTTccccctcccccacccacgccccacccccactcatccccccatcccccaccccGGGGGGGGGGGGCCAGCTCGAATGTCTACCTTGGATAGAAAGCTGACAGAAAACTCCCAGAAGGAGAGCACCAATCTTCCCCAGTGGCTCCCTGAAACAAAGGCACCTCCCATAGCTTTCTAGTTGGTTCCTCCACCCCCCACCCCCCCCCCCGTGAGTCTGGTAGGACCTTCTCTGAAAGCTGTGGTCCCACAACCTCAAAAGAGGGGGGTAGGAGAGAAAAAAAGGAAGAAAAGAACGAAGAgggtgtggttgtgcacatctttaaccctagcacccaagaggcagaaatatgtggatctctgagttccagaccaacctggtctacatagcagttttaggcaagccaggtatataataaaaAGAGGAAGCggagctggggccgcagctcagttggtaaagtccttgcctatcatacacaaagccatgagtttgatttcaacaccacataaagcaggttccatgtgggctaaggcaggaggatcagaccaagatttcaaggtcatcctctatgtatagagttggaggccagcctgagctatatgtctcaaacagggaaaaGGTAAACAGAATTATTTACTGTGAATATTATTGTAGACTGCAATAGCCCCTCGCTCACAAAATTTTTCTAGCACAAAATGTCTGAAGCTCCAAGGTTGGGAAAACGGTGTGACGCCACCTCTAACCCAGTCCTTGGGACGTGCAGAGCCAGTGAGATAGGAGCACAATGAGGAGGCGTGCAGATGGAGCGGTACACAGAGAAAAGCCAGACTCCATGTTCAGGGCCTGAGAAGTGAAAGTTTCAAATTGCAAGAAAGTTGCAAGAAAAATGAAGCTGGAATGAGGGGGTAGCAGACAGGAGTGTAGAAGAAGGCTCCCGGAATACTCCAGTCTGGGTGAGACTAGAACTTGGGAAACTCTTTGGGTGTCTGCAAGGAGTCCACAGACCAACTGACAGGGTCGTAGCTGTCAGAAGAATGATGATAAAAACGAATTCAGGGCTCTGGTGGCACCGATGCCATTGCTCAAGCCAAGCTGGAAACCGGCTACATGCCTACACCACTGGGTAAAAGTGTTCCCAGCACCGTGAGTGAACACTGCACAGCCCTAATGTTCCCCAGAGGCAGGCAAGCCTGTGAGCTCAAGAACAATTTGTTCTTACCAAGGAGCAAAAGCCATCCATCCGGGTGTTCTTACAAAAATAACCTTAAgtgatcttggacaacttaatcgtgacctcagttccgtgtcaatacaataaagaacactcaaaacaaacgtgcttatcttgggaagttatttgaatattcatgagctaaccttgtctggaacctggaacagggctctgtttgcagccagagcaggtgctctccattaaatTACACAATAGCAATGAGCAAAAGCGAGTAGACACTGCACTTGCCCACTAGGATGCCAAGTGAAGCTAGTTGTAGCTGGTTACTATCTATGACTTCCAAGTGCTAAGACAAGAATAAGATAATCTTAGAGGAGTTCCAGAAAAGTCCCAATCAGAAAGcaggcatggtggccatgcctttcctgcagaggcagaggaaagtgcatctctgagttcaaagctaccctggtctacaggtgagtgtccagcccgccagggataATGAAAGGTggagggtggtaggcagatctctgtgatctggtctacactgtctggtctacaacagcgagttactgaacaaacaggcggactctgtagaggggccttgtctcaaaccaaaaacaaaaTATACCTAGCGTTTCTAAACCAGTTTCATCTTATAATTCTTCCTCAGCACAGAGACACTCCCAATATACTCTGCATCTCTTCTCAAACTTGAGGGGGATCAAACAGCTTTGCACACCTAAGCCAGATCAGGGAGTCTAAACTGGGAATCTAAATCCCTCGTGTGACACCTGGTTTTTTAAGTTACAGGCCTGATTCAATTCTGCCCCTATTGGTAACTTAGAGGAAGTACATCCAAGTGTCCTGGGGTTGGGAGTGTAAAGAAAAACGTAAAGGAACAAAAGGCTCACATCGTGGTGCCCAGTCAGTCACCATAAAATGGGATGGGCACAGTCACCTGCATCTTGTAGGAACAGGCCTCCCAAGAGGCTCTGGGTGGCGAATGAACCACAAGACAGAAGTTAGAACTCACTTTCAGATACTCTGGTTAATTCCCTTTCCATTCCCGGGAACAGACGATTCTACCGCACGTCCCTAGGGGTGTCTACCTGTCGTGCAACCACAGTCCCATCATCGGAGAGAAAGTTCATAAAAAGAATCCAGATGTCCAAGAGGAAAATTCACTCTCCCTAGGACTAGAGAGTCTTCCTCCACTTTGTGTTTTAGGAATTGTCAAGAGTTGGCTGAATCAATAGATAAGCCCAATGGAGCCTGCAAAATCATGCCAGCCTAAAGGCAAATGTCTAAACAAACCGAATTAAAGCGAAGGGCCACGTCCCAAGATATTAGAGAAAAACCAACCGATAAACTCAAATCAGGTACCTCTCTCAAATCTGAGGTTCATCTGCCAGGGCCCTGGATACATCAAAAGCAACCGACCAACCTCTCCCACGCAGTCAGGACCAAGAGAAGAGGGGGGAGGGGGCTGGAACGTGGTCAAGGCACTTTCCCTAAAATTTGTTCCTGTGAGACACTGTAATTTCAAATGTGTAACTGAGCTCTAACACAAACCTCTGGACAGCACCCACCCTCGGCCCCTGCCCCATACTAGACCATTCCCTCTCCTAAATCCTGGACTAGGGTAGGAACCAATCCCGTAATCCTGGGTATTTTAGAAACTCAATTCTCCTGGGAGTTAGAGCGCTCCGGGCCTGGCTCCGGTCCACCCCCACCTCTCTAGCCCGAAGAGGAGAGTGAGGCTCCAAGCAGGGCCGGAGGCGCTTTCTTATGTAAATGGCGCGCCGAGAGAACTGGAGTTTGCCGGGAAGGCAGAGTGAGGTGGGCTCAGGAAGGTCAGAGCGACTAAAGGTTCATTTTCAGAAACCaagaaaacaaaacaaaaacaaaaaagaaaaaaaagaaCTCTGGGGATCGTGGGGTGGGAAGATGAGTCCATTCCGTGTAACTTTTAGTTCCAAATCAGAACATACTATCCAAAATCCGCCCTCAACTCGGCGGCCAGACACGGGGACGAGGCGGGGCGGTCCCCAGGAAGGTCTGCTGTGAGGGGGCGCCCCTTTCGAGCCTCAGTTCCGGGCAGTTCAGTTCCGGGGAGGGGGCGCGGCAGGCTAGTCTTTCTGCGCCCTTTCCCGCTCCTTCCTGGGTGGCTCTCTCCCCCACCCCACCCCCGATCCACCCCCAAGGAGCGCCACGAGGAACCGAAGGCCTCTTCAACACATCCTTAAATTCAAGAGGGCCGGGCCCCAGCTGCTACACGCTTCCACCCCGCCTCCTCCCCTAAGCAGGATTCTTCCCCACGTGCTCCTGCCGGAACTCGGGGCTGCAGAAGGGGGCTCCTCCCACGTGGGCGCAGCAGTCTCAGACTACGTGCAGGGCGGCCGTCCCATCAAGAAATTAGGGGCTCTAAGGGTGGGGAGGGTGCGCGCAGGTCCCGGGTAGGTGGGACTATCGATGGTGAGCGGTCGGGCCACGAGGGTGCCTAGCAGACGTGGCTTCTCGCGCGCAGGACTTTCCCAGCTACCCAGACTGGTTCCACGTCTCCAGCCCCGAAGGAGGAGGAAGCAGGTCCTCTGCGTGCTCCAGATTCCGGGAGAGCACGCAACTTGGTCCGAAGCGAGCCAGAAGCTCCCGCCCGTGCCGACTTACCTTCTGCACTTGGTTTTGGTTGAGCTCCGTGAATAAGCAGGCGATCATATGCGAGGCGATCATCTTGTTGCGGCGAGTCGGGGCCAAATAGAGAAACCGAGGCTCGAGCGCTACCGATGGAGCAGGCAGCGCTGTCCGACCGCTTTGAAACCAGGCCCGGGTGTCCGGCTGGGATCGAAGACTCCAGGAGCCACACAAAGAAGCTTAACCACGATGGCTCACCAGCTGAGGGCCTCAAGTTTCCAGAAACAGGGTTTTTTTTTTTCTTTTTTTCAATTTTTTTAATCACAACTACTAGAAGGTACCCTAAGTCTCACTCCTGCCGACCGGTCTAGCTGCTTAGCGTCCCGTCGCTAACTTCACTCACTGGGTCACTAAGGCTCAACTTCGGAAAAAAATGACAGTTCTGTTCCAATCCGGCGATCGCACCTATCAGAGTCACTGGCGTGATGCTCCTGCCCTCGGATCTCCAAAGCAGAACAACTCCGGGTAGCCTGTGGTGTCCTGAGACTCCAGCACTGGAGTTGGTGCAACAAT
