# Supplementary figures and images for: Activation of HIF-1α C-terminal transactivation domain protects against hypoxia-induced kidney injury through hexokinase 2-mediated mitophagy
Source: Cell Death Dis. 2023 May 24;14(5):339. doi: 10.1038/s41419-023-05854-5 (PMC10209155; doi:10.1038/s41419-023-05854-5)

HK2

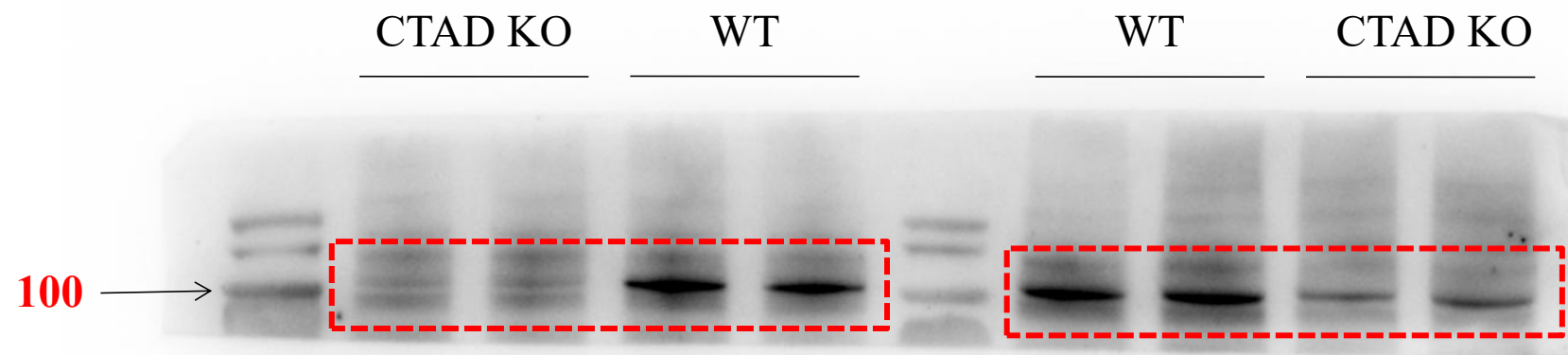

$\beta$ -actin

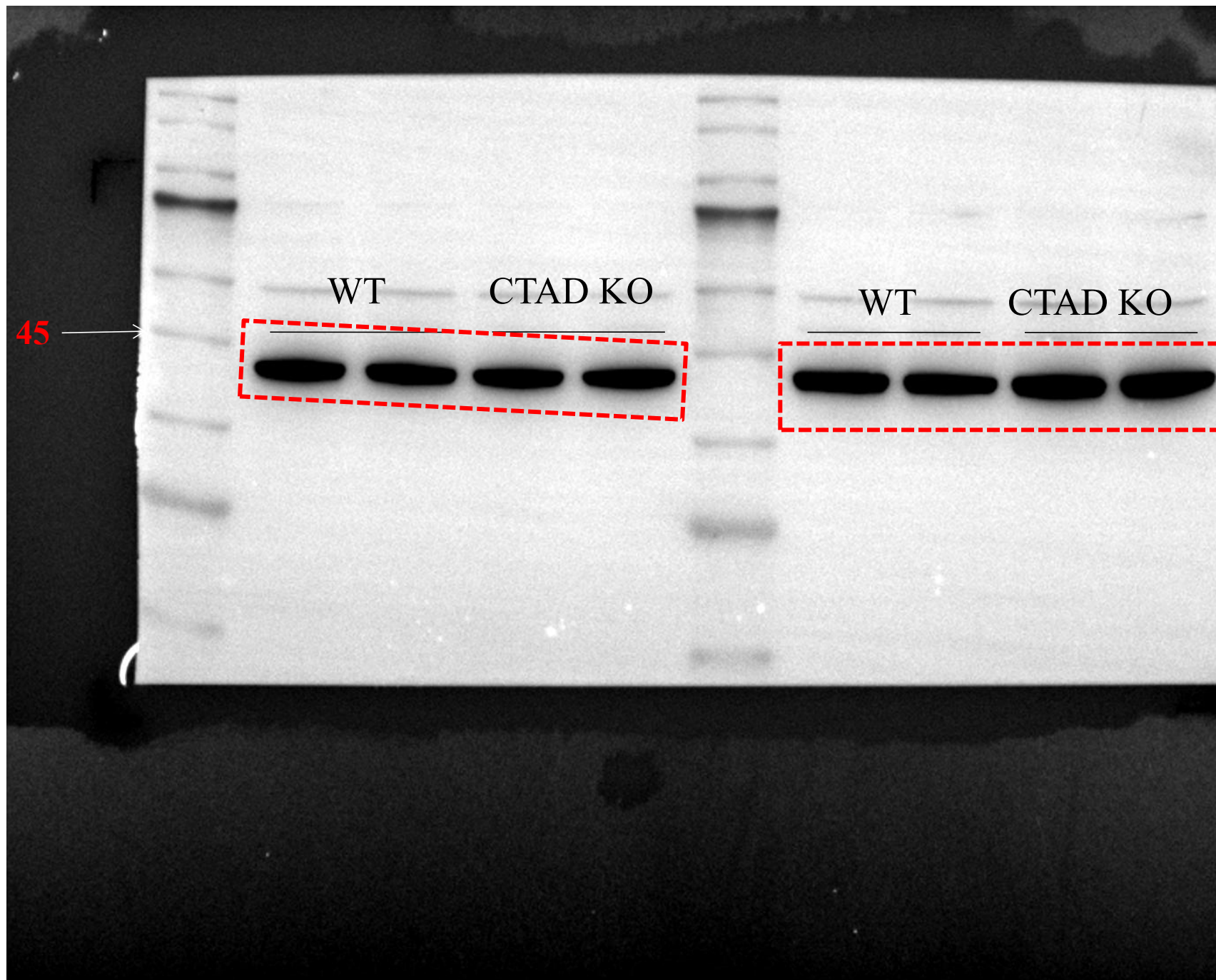

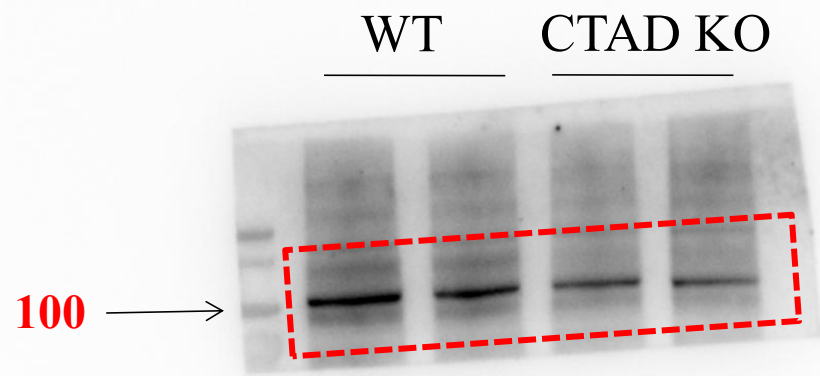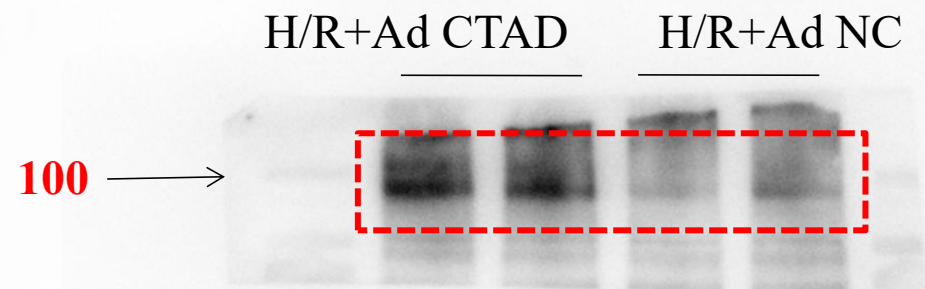

$\beta$ -actin

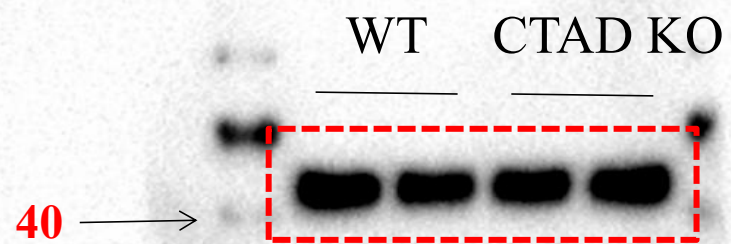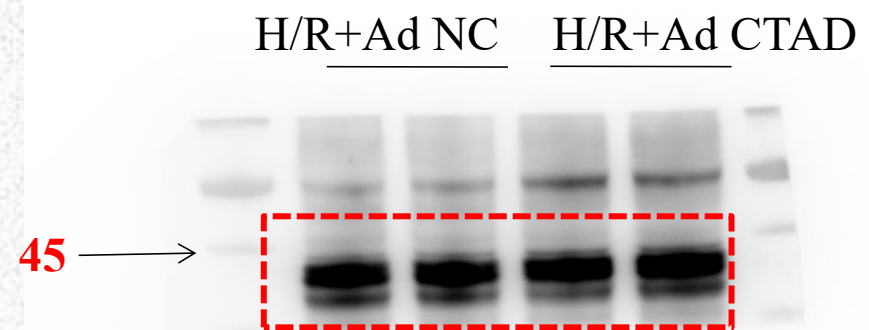

# LC3

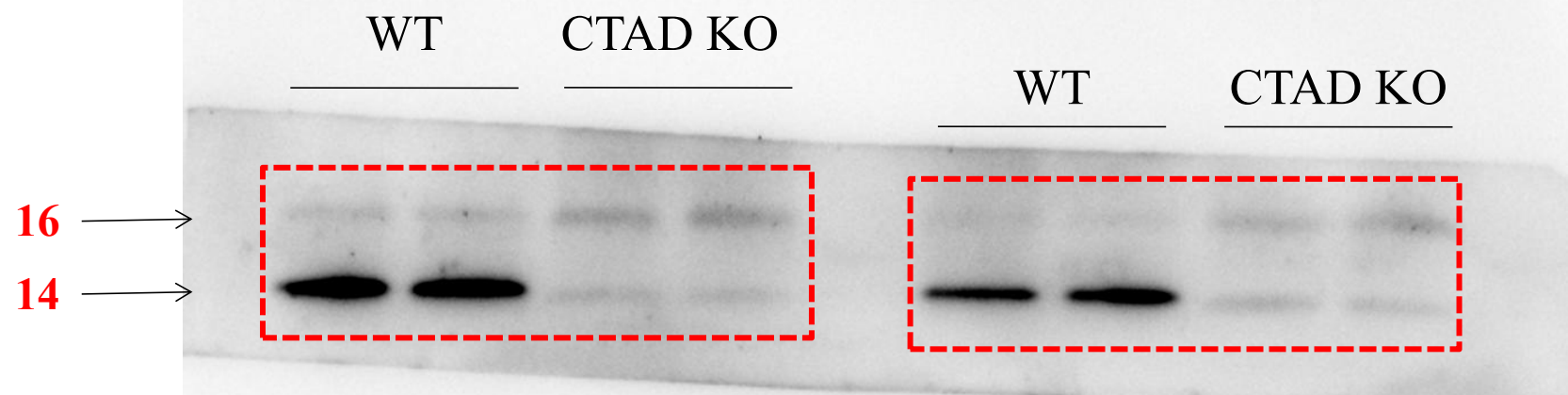

p62

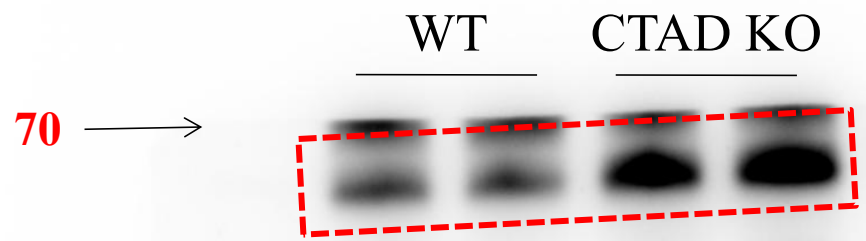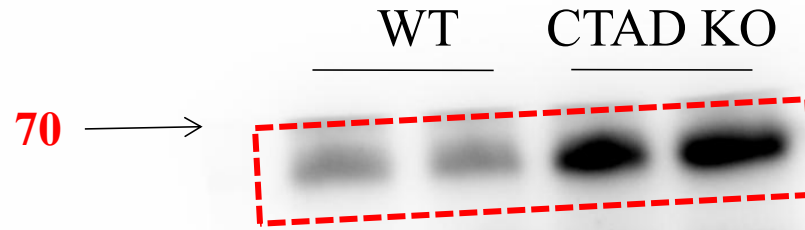

$\beta$ -actin

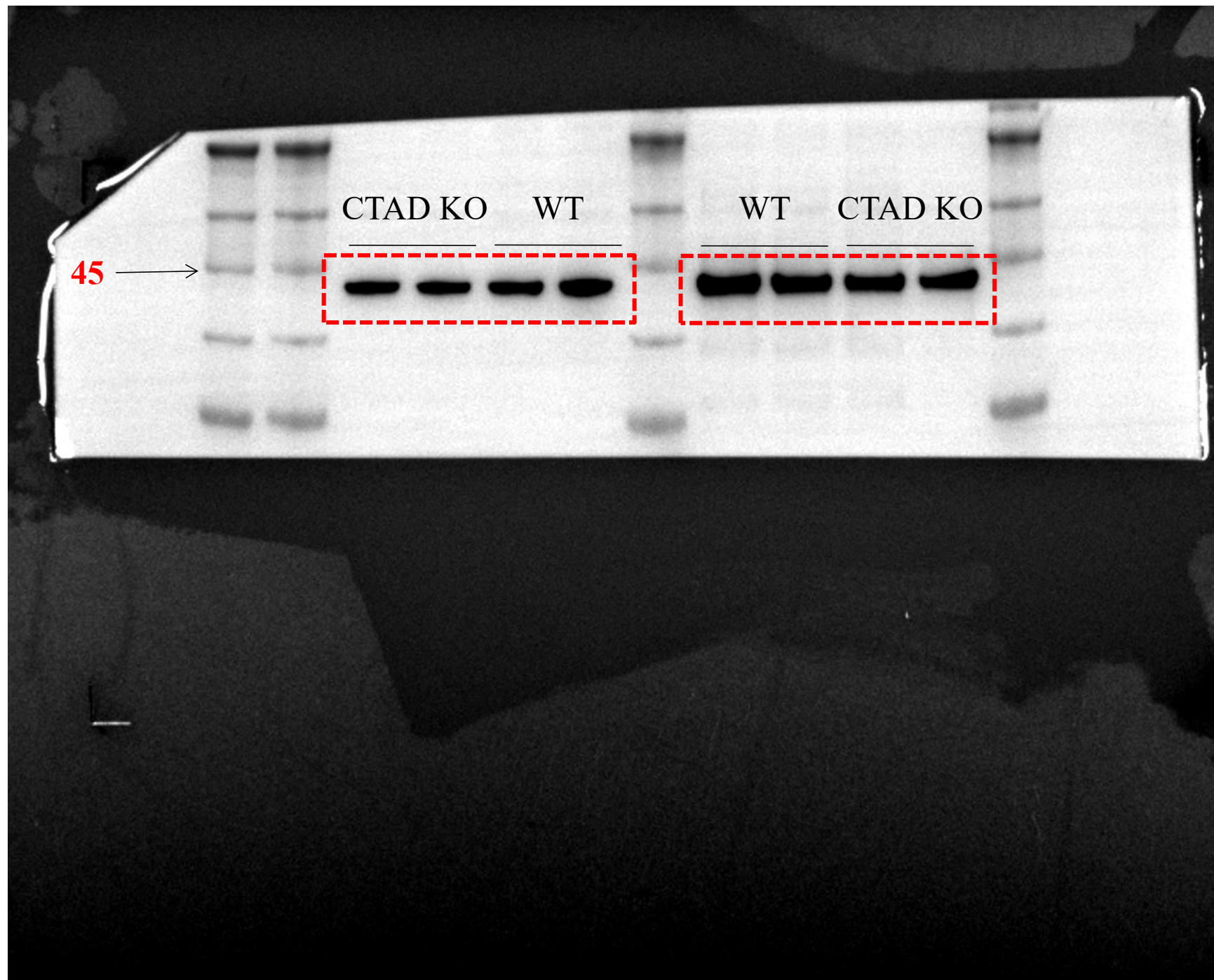

# KIM-1

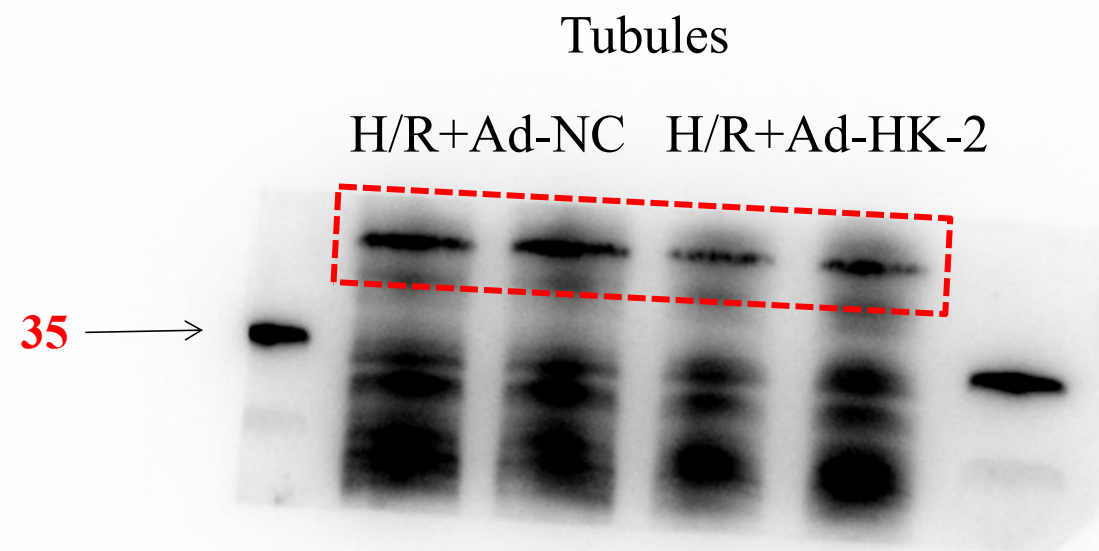

$\beta$ -actin

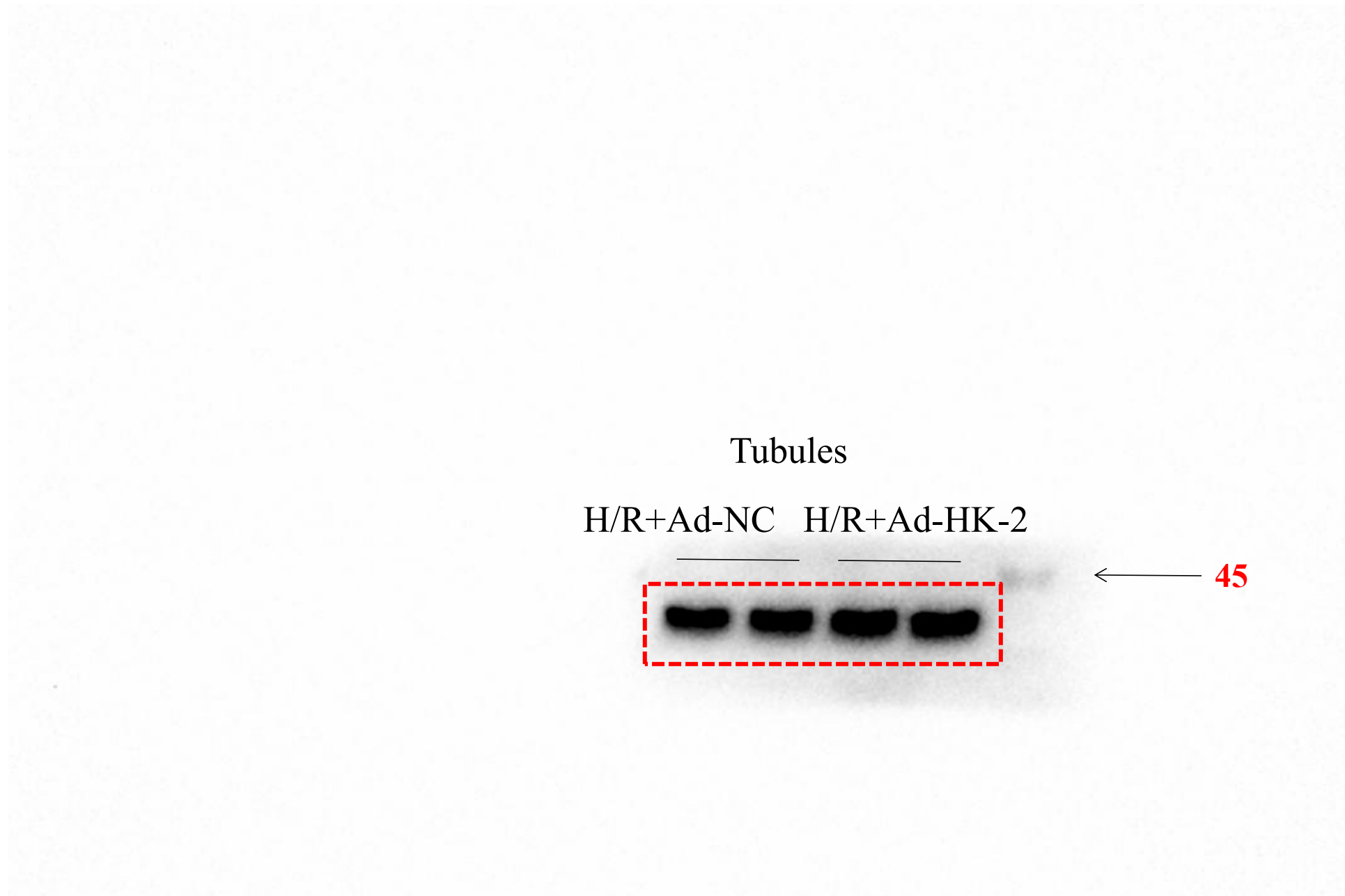

LC3

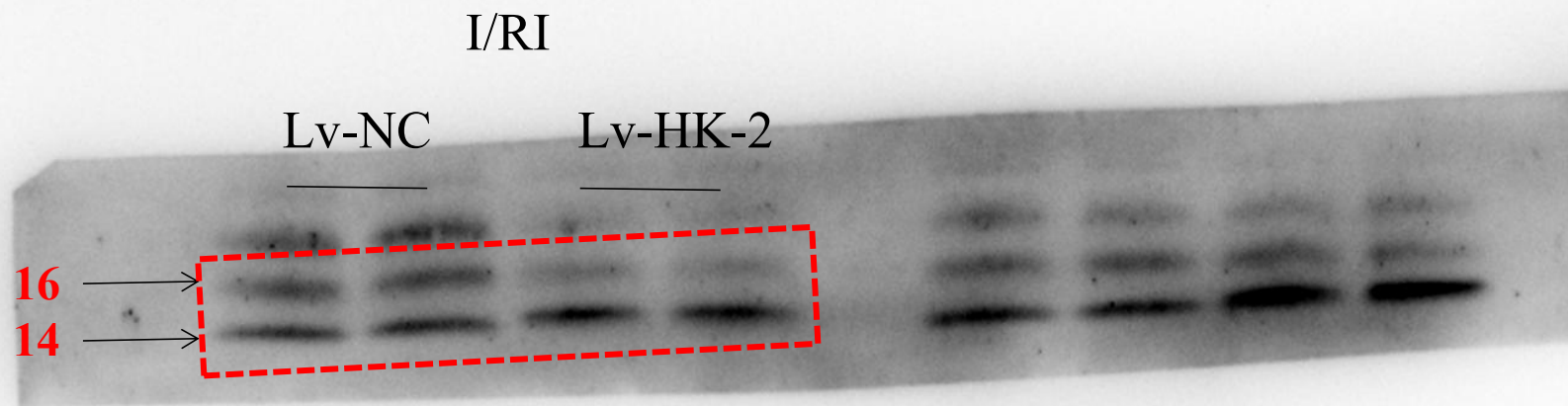

p62

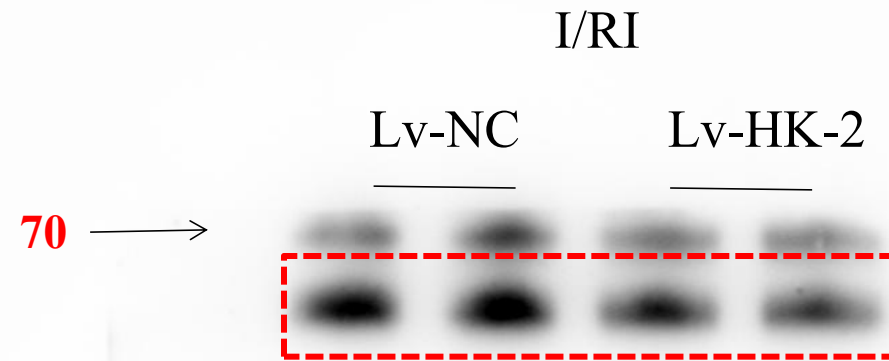

LC3

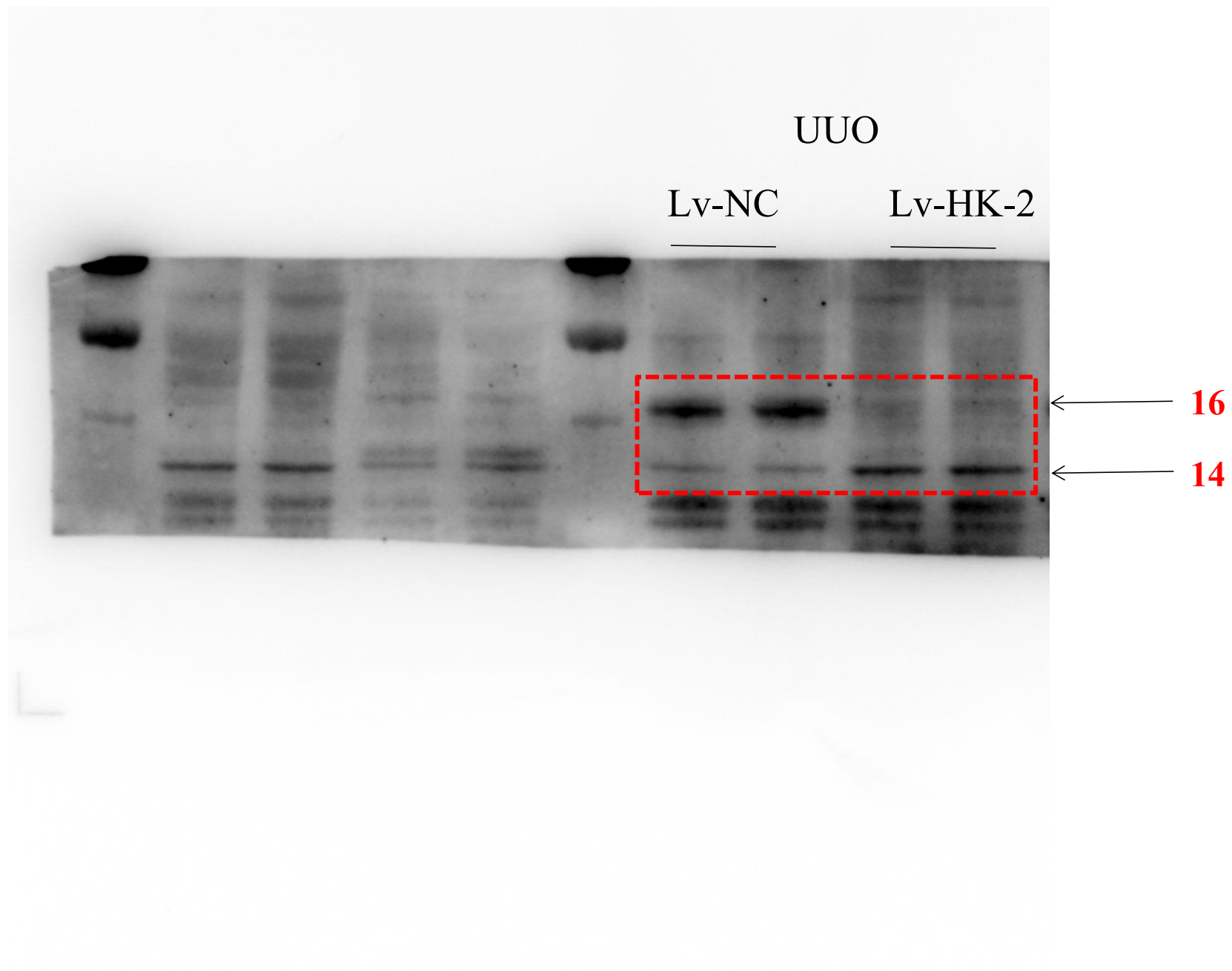

p62

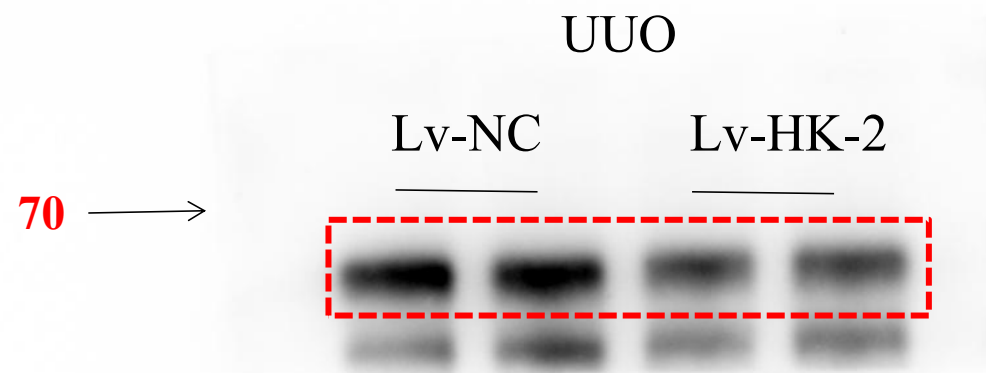

$\beta$ -actin

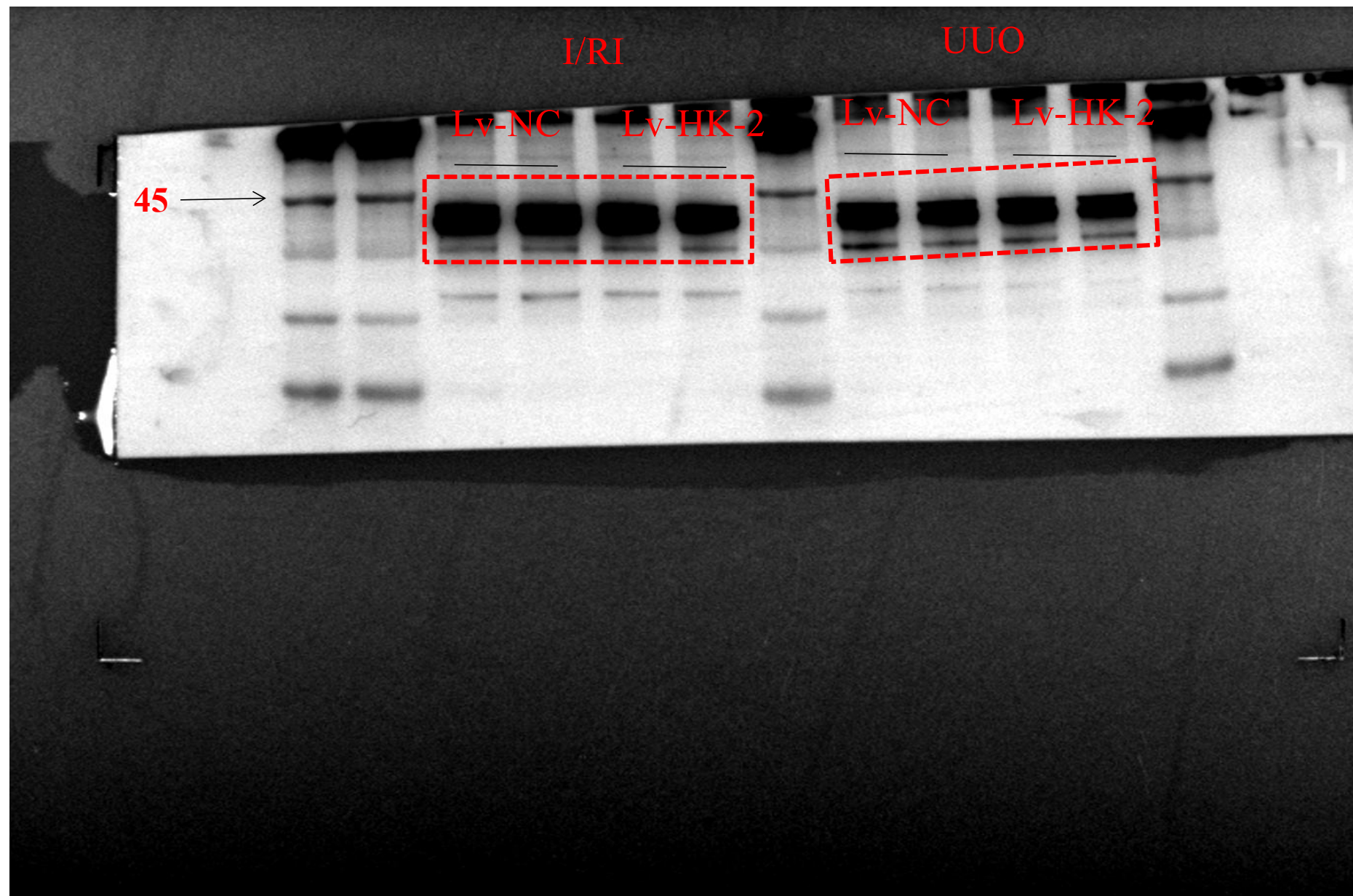

# PINK1

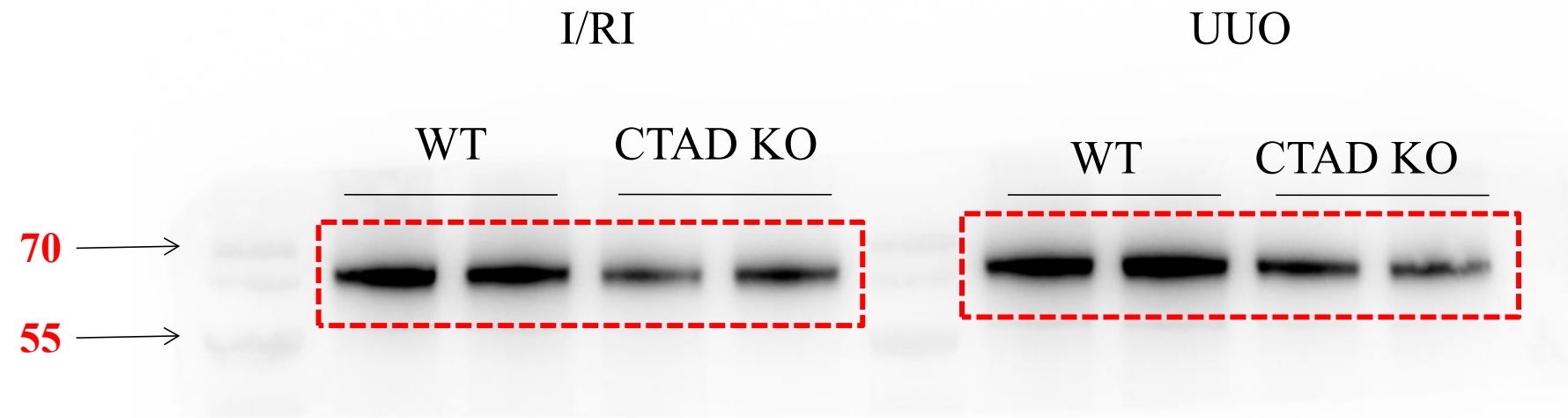

# BNIP3

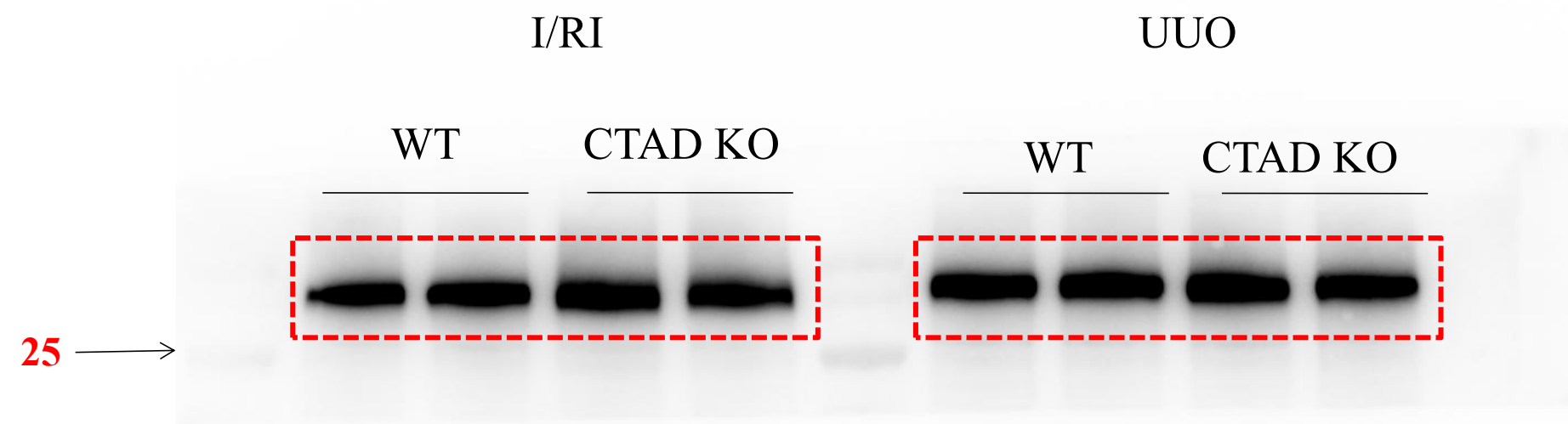

# COX IV

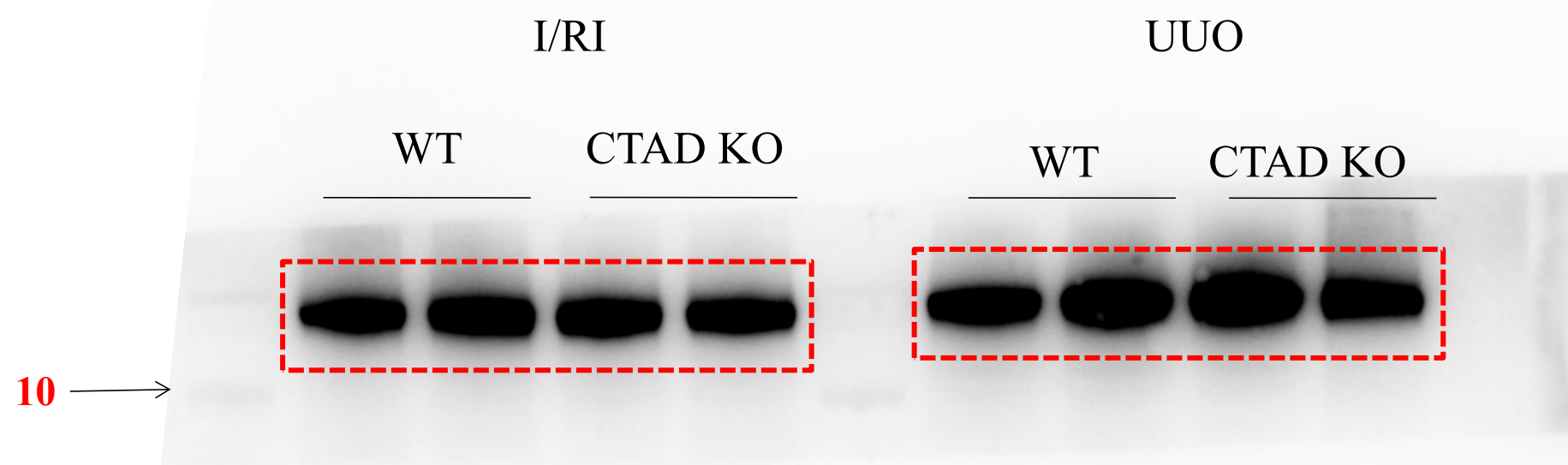

Supplement: Supplementary file 4 — Original Data File [file 41419_2023_5854_MOESM4_ESM.pdf]
